# Lipidomic profiling reveals age-dependent changes in plasma membrane lipids that affect neural stem cell aging

**DOI:** 10.1126/sciadv.aeh9771

**Published:** 2026-07-29

**Authors:** Xiaoai Zhao, Ryan M. Feitzinger, Jeeyoon Na, Xin Yan, Andrew Erickson, Kévin Contrepois, Olivia Y. Zhou, Francesco Vallania, Mathew Ellenberger, Chloe M. Kashiwagi, Stephanie D. Gagnon, Cynthia J. Siebrand, Matias Cabruja, Gavin M. Traber, Andrew McKay, Daniel Hornburg, Purvesh Khatri, Michael P. Snyder, Richard N. Zare, Anne Brunet

**Affiliations:** ^1^ Department of Genetics, Stanford University, Stanford, CA, USA.; ^2^ Department of Comparative Medicine, Yale University, New Haven, CT, USA.; ^3^ Yale Center for Molecular and Systems Metabolism, Yale University, New Haven, CT, USA.; ^4^ Institute for Stem Cell Biology and Regenerative Medicine, Stanford University, Stanford, CA, USA.; ^5^ Department of Chemistry, Stanford University, Stanford, CA, USA.; ^6^ Stanford Biophysics Program, Stanford University, Stanford, CA, USA.; ^7^ Stanford Medical Scientist Training Program, Stanford University, Stanford, CA, USA.; ^8^ Institute for Immunity, Transplantation and Infection, Stanford University, Stanford, CA, USA.; ^9^ Stanford Center for Biomedical Informatics Research, Stanford University, Stanford, CA, USA.; ^10^ Glenn Laboratories for the Biology of Aging, Stanford University, Stanford, CA, USA.; ^11^ Wu Tsai Neurosciences Institute, Stanford University, Stanford, CA, USA.

## Abstract

The aging brain exhibits a decline in the regenerative populations of neural stem cells (NSCs). While mechanisms that restore old NSC function have started to be identified, the role of lipids—especially complex lipids—in NSC aging remains largely unclear. Using lipidomic profiling by mass spectrometry, we identify age-related changes in complex lipids in quiescent NSCs in vitro and in vivo. Moreover, several polyunsaturated fatty acids increase across lipid classes in quiescent NSCs during aging. Using spatial lipidomics, we find that some of the changes in complex lipids are also observed in situ. Several age-related changes in complex lipids and side chain composition are occurring at the plasma membrane, as revealed by lipidomic profiling of isolated plasma membrane vesicles. Experimentally, we show that aging is accompanied by a decrease in plasma membrane order, a key membrane biophysical property, in old quiescent NSCs in vitro and in vivo. To determine the functional role of plasma membrane lipids in aging NSCs, we performed genetic and supplementation studies. Knocking out the phospholipid acyltransferase MBOAT2 exacerbates age-related lipidomic changes in old quiescent NSCs and impedes their ability to activate. *Mboat2* overexpression reverses age-related lipidomic changes in old quiescent NSCs and boosts their ability to activate in vitro and in vivo. Moreover, supplementation of plasma membrane lipids from young NSCs improves the ability of old quiescent NSCs to activate. Our work could lead to lipid-based strategies for restoring the regenerative potential of NSCs, which has important implications for countering brain decline during aging.

## Teaser

Plasma membrane lipids are remodeled with age in neural stem cells, and rejuvenating membrane lipids boosts old cell function.

## INTRODUCTION

Lipids are extremely diverse, yet our understanding of lipid function is lagging. Complex lipids—lipids containing additional chemical moieties linked to fatty acids (e.g., phospholipids)—are key components of plasma and organelle membranes (*1*–*4*). Complex lipids serve critical barrier function and play important roles in organellar homeostasis and signal transduction (*1*–*4*). Lipid dysregulation has been observed during brain aging and in neurodegenerative diseases such as Alzheimer’s disease (*5*–*17*). Age-associated changes in the lipidome have also been identified in several other organs (*18*–*20*), but the role of complex lipids in cells in the brain—and other organs—during aging is largely unknown.

The adult mammalian brain contains populations of neural stem cells (NSCs) that can generate neurons, astrocytes, and oligodendrocytes (*21*–*27*). NSCs are organized in two main niches—the dentate gyrus of the hippocampus and the subventricular zone (SVZ) (*25*, *26*, *28*–*31*). During aging, the ability of NSCs to transition from quiescence to activation declines (*32*–*44*), and this deterioration could underlie defects in aspects of cognitive and sensory function (*22*, *23*, *45*, *46*) as well as impaired injury repair in old individuals (*47*–*50*). Understanding how to maintain NSC regenerative potential during aging could identify strategies to counter age-related decline and facilitate repair after injury in the central nervous system (*51*–*68*). While lipid energy metabolism has been shown to be important for NSCs and other cells (*69*–*81*), the contribution of complex lipids to cell function during aging has not been well studied. More generally, a systematic examination of the global lipidome of mammalian cells during aging is still missing.

## RESULTS

### Lipidomics reveals remodeling of several membrane lipids in old quiescent NSCs in vitro

**Fig. 1.**
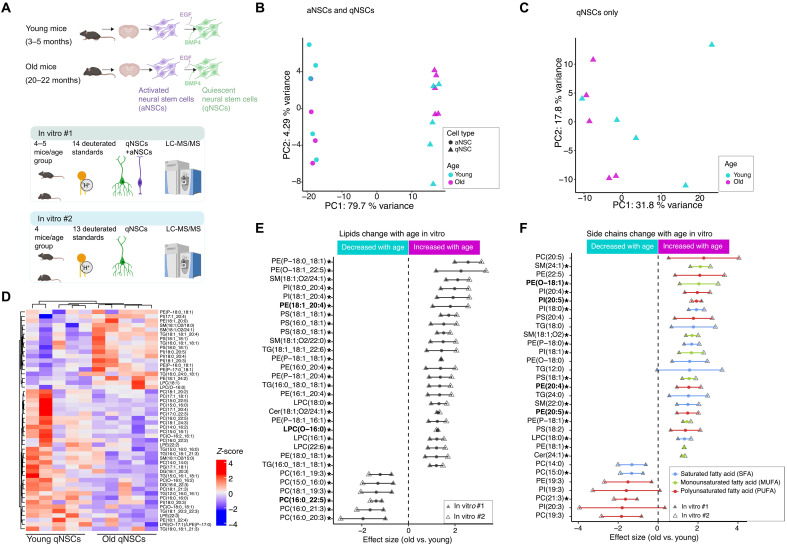
Lipidomics on young and old qNSCs in vitro identifies changes in membrane lipid and side chain composition with age. (**A**) Overview of two lipidomic studies in vitro. Primary NSC cultures were established from young and old mice. Each culture was established from an individual mouse. For each experiment (In vitro #1 and In vitro #2), number of animals used, number of internal deuterated lipidomic standards used, and neural stem cell type (qNSCs and aNSCs) included are indicated. Created in BioRender. lab, Z. (2026) https://BioRender.com/zqn17e8 (upper panel). Created in BioRender. lab, Z. (2026) https://BioRender.com/2ujzkut (lower panel). (**B** and **C**) Principal component analysis (PCA) of In vitro #1 lipidomics. PCA was performed on aNSCs and qNSCs (B) or qNSCs only (C). Each symbol represents an individual primary aNSC culture (dot) or qNSC culture (triangle), established from individual young (cyan) or old (purple) mice. (**D**) Heatmap with clustering on the concentration of 50 lipids with significant change with age (FDR-adjusted *P* < 0.1) in qNSCs from In vitro #1 lipidomics. (**E** and **F**) Lipids and side chain composition change with age in qNSCs of In vitro #1 and In vitro #2. Significant lipids of top 30% overall change (E) and side chain features that exhibit the top 20% overall change (F) in effect size with age in both in vitro studies are plotted. Effect size for each lipid or side chain feature from each study (triangle) is shown together with mean effect size (dot) and SEM (error bar) from 2 studies. Statistical significance was assessed based on the *P* value from test statistics using the 95% confidence interval of the data, and multiple hypothesis correction was done by FDR. Black asterisk indicates significance (FDR < 0.05). Bold labels indicate lipids or side chain features of interest that significantly change with age and with *Mboat2* interventions (see Figs. 6 and 7).

To unbiasedly determine the age-related changes in complex lipids, we performed untargeted lipidomic profiling of NSCs from young and old mice in vitro and in vivo. Using liquid chromatography followed by tandem mass spectrometry (LC-MS/MS), we profiled quiescent and activated NSCs (qNSCs and aNSCs, respectively) cultured from young (3 to 5 months) and old (20 to 22 months) male mice (Fig. 1A, “In vitro #1”). We performed LC-MS/MS experiments on *n* = 4 to 5 primary NSC cultures per age group (each derived from an individual mouse) using 14 internal standards (Fig. 1A and table S1). This LC-MS/MS dataset detected 367 unique lipids in aNSCs and in qNSCs, covering all major lipid classes—phospholipids [e.g., phosphatidylcholine (PC) and phosphatidylethanolamine (PE)], sphingolipids, triglycerides, and cholesterol (fig. S1, A and B, and table S3). Principal component analysis (PCA) on the lipidomes showed good separation between qNSCs and aNSCs (Fig. 1B) as well as some separation between ages for qNSCs (but not aNSCs) (Fig. 1C). We therefore focused the rest of the manuscript on the differences in lipids with age in qNSCs. The lipid composition of young and old qNSCs was generally similar at the lipid class level (including cholesterol) (fig. S1A), but several individual lipids significantly changed with age in qNSCs [false discovery rate (FDR)–adjusted *P* < 0.1]—including many membrane lipids (e.g., PC, PE, and sphingomyelin) (Fig. 1D).

To characterize lipids that consistently change with age across experiments, we generated an independent LC-MS/MS dataset on *n* = 4 young (3 to 5 months) and *n* = 4 old (20 to 22 months) primary qNSC cultures using 13 internal standards (Fig. 1A, “In vitro #2”, and tables S1 and S3). This dataset showed similar lipid class composition (except for cholesterol, due to the lack of quantitative cholesterol standard in this experiment; see Materials and Methods) (fig. S1C). This experiment detected 259 lipids, and 175 of them overlapped with the first in vitro experiment (fig. S1B). More than 90% of the molar content of the samples was represented by lipids measured in both experiments (fig. S1B). Among the lipids that overlapped between both datasets, lipid concentrations were strongly correlated in both young (*R* = 0.91, *P* < 2.2 × 10^−16^) and old (*R* = 0.9, *P* < 2.2 × 10^−16^) qNSCs (fig. S1D and table S3). Moreover, PCA revealed that age-related changes in qNSCs were consistent in direction across both datasets for lipids that overlapped between the two experiments (fig. S1E). We identify 92 lipids that commonly increased with age and 37 lipids that commonly decreased with age in both in vitro experiments (fig. S1B). There was some variability between experiments, likely reflecting differences in primary cell cultures, cohorts of aging mice, and experimental design (see Materials and Methods).

To identify lipids that robustly change with age in qNSCs across both datasets, we calculated effect size (i.e., differences between the group means of young and old samples divided by the pooled standard deviation, with 95% confidence interval and multiple hypothesis correction; see Materials and Methods). This type of approach has been used for comparisons across datasets, including lipidomic and metabolomic datasets (*82*–*84*). Several membrane lipids showed a consistent and significant increase [e.g., PE(P–18:0_18:1) and PE(18:1_20:4)] or decrease with age [e.g., PC(16:0_20:3) and PC(16:0_22:5)] in qNSCs across both in vitro datasets (FDR-adjusted *P* < 0.05) (Fig. 1E, figs. S1F and S3, and table S3). Some of these lipids [e.g., PE(18:1_20:4)] were of high abundance, constituting more than 1% of the total lipid molar concentration (fig. S1G). Thus, several membrane lipids in qNSCs, including relatively abundant ones, are consistently remodeled with age.

### Changes in lipid side chain composition with age in quiescent NSCs in vitro

Complex lipids are composed of one or more fatty acyl side chains, and side chain carbon chain length, degree of unsaturation, and linkage type all contribute to biophysical properties, especially in membranes (*1*, *3*). For each lipid class, we quantified the abundance of each unique fatty acyl side chains in vitro. The molar concentration of lipids containing a given side chain of each lipid class was strongly correlated in young and old qNSCs across in vitro LC-MS/MS datasets (*R* = 0.95, *P* < 2.2 × 10^−16^ in young and *R* = 0.94, *P* < 2.2 × 10^−16^ in old, fig. S4A).

Effect size calculation showed that several highly unsaturated polyunsaturated fatty acid (PUFA)–containing side chains {e.g., PC- and phosphatidylinositol (PI)-containing eicosapentaenoic acid [PC(20:5) and PI(20:5)] or arachidonic acid [PI(20:4) and PE(20:4)]} consistently increased with age in both in vitro datasets, whereas phospholipids containing less unsaturated PUFAs [e.g., PC(19:3) and PC(21:3)] consistently decreased with age (Fig. 1F and fig. S4B). We also detected age-related changes in lipids containing monounsaturated fatty acid (MUFA) and saturated fatty acid (SFA) side chains (Fig. 1F and fig. S4B). Some features that exhibit significant change with age were among side chains that have the highest concentration in cells [e.g., PE(20:4) and PI(20:4)], while others such as PI(20:5) and PE(20:5) were less abundant (fig. S4C). Hence, qNSCs also show several age-related changes in the composition of membrane-lipid fatty acyl side chains in vitro.

### Lipidomic analysis shows remodeling of membrane lipids in old qNSCs in vivo

**Fig. 2.**
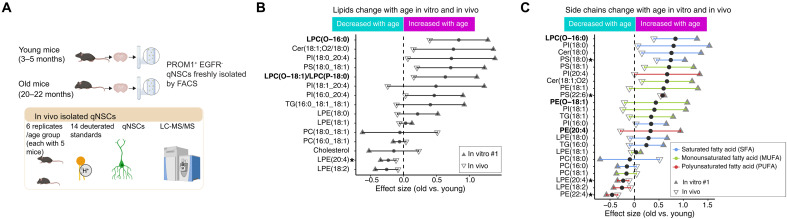
Lipidomic analysis shows remodeling of membrane lipids in old qNSCs in vivo. (**A**) Schematic of lipidomic profiling on qNSCs in vivo. Quiescent NSCs were freshly isolated from the brain of *n* = 30 young and *n* = 30 old mice (*n* = 6 qNSC samples, each from five pooled mice) using fluorescence-activated cell sorting (FACS) based on the positive staining of Prominin-1 (PROM1^+^) and negative staining of epidermal growth factor receptor (EGFR^−^). Number of animals used, number of internal deuterated lipidomic standards used, and neural stem cell type included are indicated. Created in BioRender. lab, Z. (2026) https://BioRender.com/f0gaa5r (upper panel). Created in BioRender. lab, Z. (2026) https://BioRender.com/2ujzkut (lower panel). (**B** and **C**) Lipids and side chain composition change with age in qNSCs in vitro and in vivo. Effect size of lipids from In vitro #1 (filled triangle) and In vivo isolated qNSCs (upside-down triangle) is plotted together with mean effect size (black dot) and SEM (error bar) from two studies (B). Effect size of side chain composition features from In vitro #1 (filled triangle) and In vivo isolated qNSCs (upside-down triangle) is plotted together with mean effect size (solid dot) in blue, green, and red that represent saturated fatty acid (SFA), monounsaturated fatty acid (MUFA), and polyunsaturated fatty acid (PUFA), respectively (C). Error bar represents SEM from two studies. Statistical significance was assessed based on the *P* value from test statistics using the 95% confidence interval of the data, and multiple hypothesis correction was done by FDR. Black asterisk indicates significance (FDR < 0.05). Bold labels indicate lipids or side chain features of interest that significantly change with age and with *Mboat2* interventions (see Figs. 6 and 7) in vitro.

Could some of the lipid changes identified in qNSCs in vitro be detected in vivo? We performed lipidomic profiling on qNSCs freshly isolated by fluorescence-activated cell sorting (FACS) from the SVZ neurogenic niche of young (3 to 5 months) and old (20 to 22 months) mice (*32*, *85*, *86*) (Fig. 2A, fig. S5 for the FACS gating strategy, and tables S1 and S5). As qNSCs are relatively rare in the mouse brain (∼1500 per mouse), we pooled cells from five mice to obtain ∼9000 qNSCs for each of the *n* = 6 biological replicates per age group and we performed untargeted lipidomics using LC-MS/MS with 14 internal standards (Fig. 2A). We identified 29 lipids in qNSCs freshly isolated from the brain, encompassing the major lipid classes identified in qNSC primary cultures [there were more lysophosphatidylethanolamine (LPE) lipids detected in vivo, perhaps due to systemic influences in vivo that are not present in vitro] (figs. S6A and S2B). Only 15 of these detected lipids overlapped between in vivo and in vitro datasets (six increasing with age and two decreasing with age) (fig. S6B). Moreover, in vivo detected lipids only represent ∼30% of the molar content of the in vitro samples (fig. S6B), likely due to the low starting material. Nevertheless, among the lipids that overlapped between in vitro and in vivo datasets, lipid concentrations were strongly correlated for both young and old qNSCs across a few membrane lipid classes (*R* = 0.98, *P* = 1.3 × 10^−5^ in young, *R* = 0.95, *P* = 0.00026 in old) (fig. S6, C and D). By calculating effect sizes, we identified several lipids [e.g., LPC(O-16:0) and LPC(O-18:1)/LPC(P-18:0)] that tended to increase with age both in vitro and in vivo as well as a membrane lipid LPE(20:4) that significantly decreased with age both in vitro and in vivo (Fig. 2B).

Quantification of lipids with unique side chains of each class revealed that side chain composition correlated between in vitro and in vivo datasets in both young (*R* ∼ 0.47, *P* = 0.022) and old (*R* ∼ 0.48, *P* = 0.017) qNSCs (fig. S6E). Notably, phosphatidylserine (PS) with 18:0 or 22:6 side chain significantly increases with age while PE(22:4) and LPE(20:4) decrease with age both in vitro and in vivo (Fig. 2C). Hence, we could detect some age-dependent changes in membrane lipids in qNSCs in vivo, although in vivo detection was limited due to the low starting material.

### Spatial lipidomic profiling shows changes in membrane lipids in qNSCs in situ

**Fig. 3.**
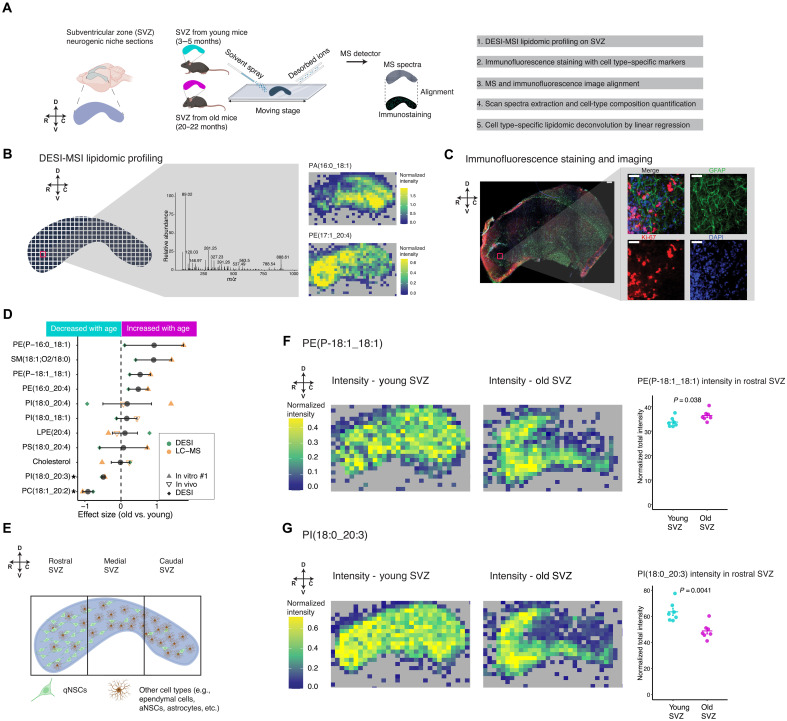
In situ lipidomic profiling by DESI-MSI validates specific complex membrane lipid change with aging. (**A**) Lipidomic study in situ using desorption electrospray ionization mass spectrometry imaging (DESI-MSI). Arrows indicate orientation axes, with R: rostral, C: caudal, D: dorsal, and V: ventral. Created in BioRender. lab, Z. (2026) https://BioRender.com/lb21w0i. (**B**) Left: Representative mass spectra from an individual pixel (individual DESI-MSI scan) showing the abundance of metabolites (including lipids). Right: Representative DESI-MSI image showing the tissue distribution of PA(16:0_18:1) and PE(17:1_20:4) across the SVZ neurogenic niche. (**C**) Left: Immunofluorescence staining of SVZ section, postlipidomic profiling by DESI-MSI with GFAP (NSC and astrocyte marker, green), Ki67 (proliferation marker, red), and DAPI (nuclei, blue). Scale bar, 200 μm. Right: Enlarged images covering the area of one individual DESI-MSI scan. Scale bar, 50 μm. (**D**) Age-associated change in lipids. Effect size of lipids from In vitro qNSCs, In vivo isolated qNSCs, and qNSCs/astrocytes from DESI-MSI is plotted together with mean effect size (black dot) and SEM (error bar) from three studies. Statistical significance was assessed based on the *P* value from test statistics using the 95% confidence interval of the data, and multiple hypothesis correction was done by FDR. Black asterisk indicates significance (FDR < 0.05). (**E**) Schematic of spatial distribution of qNSCs (green) versus other cell types (brown) across the SVZ in three equally sized regions along the rostral-caudal axis. Created in BioRender. lab, Z. (2026) https://BioRender.com/jq8a65b. (**F** and **G**) Lipid abundance in situ. Left and middle: Heatmaps of PE(P-18:1_18:1) (F) and PI(18:0_20:3) (G) in a representative young (left) and old (middle) SVZ are shown. Right: Quantification of PE(P-18:1_18:1) (F) and PI(18:0_20:3) (G) in the rostral region of the SVZ. Dot and whisker plot with each individual dot representing data from an individual animal. *n* = 7, whisker: ±SEM. *P* values from Wilcoxon rank-sum test.

We next asked whether we could also observe age-related changes to complex membrane lipids in qNSCs in the neurogenic niche in situ, without subjecting cells to tissue dissociation and FACS. To this end, we used desorption electrospray ionization mass spectrometry imaging (DESI-MSI) on whole mount sections of SVZ neurogenic niches (Fig. 2A and table S6). DESI-MSI is a spatial lipidomics method that allows simultaneous in situ detection of lipids and metabolites while preserving tissue integrity (*87*–*89*) (Fig. 3A).

We acquired DESI-MSI spectra on SVZ neurogenic niche sections from *n* = 7 young (3 to 5 months) and *n* = 7 old (20 to 22 months) mice (one SVZ whole mount section from each animal). The SVZ neurogenic niche is heterogeneous and comprises several cell types (including qNSCs and aNSCs) (*29*, *90*–*92*). We used sections of SVZ whole mount to assess cell layers that are immediately adjacent to the ventricle, where a high density of NSCs reside. As DESI-MSI has a resolution of 200 μm, the mass spectrum from each individual scan of the SVZ encompasses multiple cell types (Fig. 3, A to C). To obtain cell type–specific lipidomic profiles in situ in the SVZ, we used a deconvolution method we had previously developed and validated on brain sections with heterogeneous composition of neurons and astrocytes (*89*). We first aligned DESI-MSI spectra with immunofluorescence images acquired on the same tissue section stained with cell type–specific markers [GFAP^+^Ki67^−^ for qNSCs/astrocytes, GFAP^+^Ki67^+^ for aNSCs, and GFAP^−^Ki67^+^ for neural progenitor cells (NPCs)] (Fig. 3, A to C). We then applied our deconvolution method, using linear regression across heterogeneous sections (*89*, *93*), on each DESI-MSI spectrum with its associated cell-type composition from immunofluorescence images (see Materials and Methods for details) (Fig. 3A). To test whether our previously developed deconvolution method (*89*) could be applied to the SVZ neurogenic niche, we verified that it yielded accurate estimates of cell type–specific profiles from a reconstituted in silico mixture lipidomic data composed of our own (present study) and published datasets (*6*) (see Materials and Methods for details) (fig. S7, A and B). We also asked whether our deconvolution method could distinguish metabolomic profiles of different cell types (e.g., qNSCs/astrocytes versus aNSCs). PCA could not separate the metabolomic profiles of qNSCs/astrocytes and aNSCs. We therefore used a supervised dimensionality reduction approach—orthogonal partial least squares-discriminant analysis (OPLS-DA)—to distinguish the metabolomic profiles of qNSCs/astrocytes and aNSCs (as well as young and old qNSCs/astrocytes) (fig. S7, C to F). Although supervised and therefore less unbiased than PCA, OPLS-DA is better suited to datasets containing highly correlated features—such as co-regulated metabolites within the same pathway—a pattern that is prevalent in DESI-MSI given its broad metabolic coverage (*94*, *95*). We examined the performance of the OPLS-DA models and assessed potential overfitting—a common concern for a supervised approach. We observed that OPLS-DA separated the metabolomic profiles of qNSCs/astrocytes and aNSCs following deconvolution of DESI-MSI data and that the model performance was good (fig. S7, C and D). OPLS-DA could also distinguish qNSCs/astrocytes from young and old animals, although the model performance was not good in this case (fig. S7, E and F), likely due to a lack of sensitivity.

While the DESI-MSI approach was not sensitive enough to allow a global analysis of the lipidome with age in situ, we used it to identify specific lipids that significantly change with age in qNSCs/astrocytes in the neurogenic niche. We performed an additional tandem mass spectrometry on the same samples and annotated 11 highly abundant membrane lipid species (fig. S8). We then compared the age-associated effect sizes of these lipids from DESI-MSI to those obtained in vitro or in vivo from LC-MS/MS. Several membrane lipids exhibited consistent age-dependent abundance changes in qNSCs/astrocytes by DESI-MSI and in qNSCs across in vitro or in vivo LC-MS/MS datasets, including PE(P-18:1_18:1), which showed a trend toward increased abundance with age, and PI(18:0_20:3), which showed a significant decreased abundance with age (Fig. 3D). Hence, DESI-MSI could detect membrane lipid species that consistently change with age in qNSCs in the context of intact SVZ neurogenic niches.

We next examined the spatial pattern of membrane lipids that show age-related changes. We focused on PE(P-18:1_18:1) and PI(18:0_20:3), which show an increased or decreased abundance with age, respectively, in qNSCs/astrocytes (Fig. 3D). We divided the SVZ into three equally sized regions along the rostral-caudal axis, as it is known that qNSCs are more numerous in the rostral region of the SVZ than niche astrocytes (*90*–*92*) (Fig. 3E). We quantified lipid intensity for these specific lipids in each region overall (which encompasses multiple cell types) (see Materials and Methods for details). Spatially, we found that PE(P-18:1_18:1) abundance increased with age whereas PI(18:0_20:3) decreased with age in the rostral region of the SVZ—the region that contains the highest density of qNSCs (*90*–*92*) (Fig. 3, F and G). Age-related changes of these two lipids were also detected outside of the rostral region [and in a different direction for PE(P-18:1_18:1)], likely due to changes in other cell types (fig. S7, G and H). Thus, spatial lipidomic profiling by DESI-MSI was able to detect complex membrane lipids that change with age in qNSCs in the intact SVZ neurogenic niche.

### Plasma membrane lipids are affected during aging

**Fig. 4.**
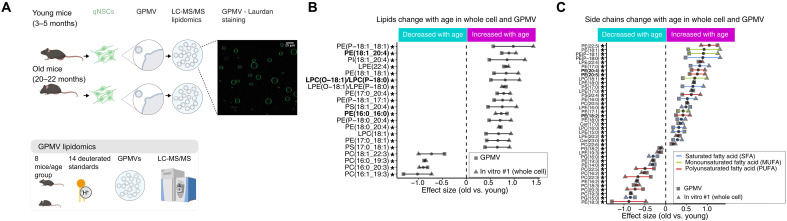
Plasma membrane lipids change with age in qNSCs. (**A**) Giant plasma membrane vesicles (GPMVs) were generated from quiescent neural stem cell (qNSC) primary cultures from young and old mice. Lipids extracted from GPMVs were analyzed by lipidomics using LC-MS/MS. Confocal image showing isolated GPMVs visualized by staining with Laurdan. Number of animals used and number of internal deuterated lipidomic standards used are indicated. Created in BioRender. lab, Z. (2026) https://BioRender.com/kgqhtr8 (upper panel). Created in BioRender. lab, Z. (2026) https://BioRender.com/2ujzkut (lower panel). (**B** and **C**) Lipids and side chain composition change with age in whole cells and GPMVs from qNSC primary cultures. Significant lipids that exhibit the top 30% overall change in effect size (B) and all significant side chain composition change with age (C) in both studies are plotted. Effect size of lipids or side chain compositions from In vitro #1 (filled triangle) and GPMV from qNSCs (square) is plotted together with mean effect size (dot) and SEM (error bar) from both studies. Statistical significance was assessed based on the *P* value from test statistics using the 95% confidence interval of the data, and multiple hypothesis correction was done by FDR. Black asterisk indicates significance (FDR < 0.05). Bold labels indicate lipids or side chain features of interest that significantly change with age and with *Mboat2* interventions (see Figs. 6 and 7) in vitro.

Membrane lipids are major components of the plasma membrane and organelle membrane (endoplasmic reticulum, Golgi apparatus, lysosome, mitochondrion, etc.) (*96*, *97*). As the plasma membrane is one of the largest membrane compartments in the cell, we asked whether some of the lipid changes in old qNSCs could indeed occur at the level of the plasma membrane. We isolated the plasma membrane of qNSCs by generating giant plasma membrane vesicles (GPMVs) (Fig. 4A). GPMVs do not contain organellar lipids and represent mostly plasma membrane lipids in cells (*98*, *99*). We verified that GPMVs from qNSCs were depleted in organelles and other cellular compartments by Western blot (fig. S9A). We then performed untargeted lipidomics using LC-MS/MS with 14 internal standards on GPMVs generated from qNSCs from *n* = 8 young (3 to 5 months) and *n* = 8 old (20 to 22 months) mice (Fig. 4A and tables S1 and S7). Our lipidomic analysis confirmed that GPMVs contained prominent plasma membrane lipid species, including PC, PE, PS, and cholesterol (fig. S9B). Overall, we detected 267 lipids in the GPMV dataset, 186 of which overlapped with the In vitro #1 (whole cell extract) dataset (fig. S9C). Of these overlapping lipids, 78 lipids increased with age and 46 lipids decreased with age (fig. S9C).

To identify consistent changes between GPMVs and in vitro datasets, we calculated effect sizes for these datasets. We found that lipids such as PE(P-18:1_18:1) and PE(18:1_20:4) significantly increased with age whereas PC(16:1_19:3) significantly decreased with age in both whole cell extracts and GPMVs (Fig. 4B and fig. S9D). Some of the lipids that change with age in GPMVs were abundant lipids (fig. S9E).

We also examined the side chain profiles of GPMV lipids. Similar to whole cell in vitro datasets, phospholipids containing highly unsaturated PUFAs significantly increased with age in both whole cell extracts and GPMVs, including PE(22:5), PE(20:4), and PE(20:5) (Fig. 4C and fig. S9F). Lipids with less unsaturated PUFA side chains, including PE(18:3), PC(19:3), and PC(20:3), significantly decreased with age in both whole cell extracts and GPMVs (Fig. 4C and fig. S9F). We also detected age-related changes in lipids containing MUFA and SFA side chains (Fig. 4C and fig. S9F). Some features that exhibit significant change with age were among side chains that have the highest concentration in GPMV and whole cells [e.g., PE(20:4) and PE(18:1)], while others such as PE(18:3) were less abundant (fig. S9G). Together, these data raise the possibility that several of the lipid changes in old qNSCs occur at the level of the plasma membrane (although some lipid changes may also occur in other organelles).

### Plasma membrane order is affected by age

**Fig. 5.**
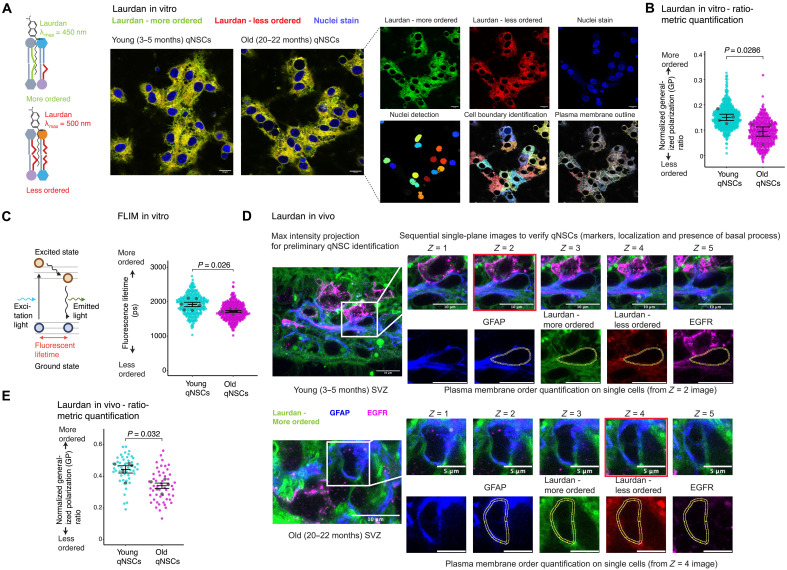
Plasma membrane order changes with age in qNSCs in vitro and in vivo. (**A**) Plasma membrane order assessed in vitro by ratiometric quantification of Laurdan staining on primary qNSC cultures. Left: Schematics of the membrane order assessment by Laurdan. Middle: Image from Laurdan rigid wavelength (green) overlaid with Laurdan fluid wavelength (red) and nuclei staining (blue) is shown from young and old qNSCs. Right: Confocal images from individual wavelength (top) and steps of automated image analysis pipeline for plasma membrane order quantification (bottom). Scale bar, 10 μm. Created in BioRender. lab, Z. (2026) https://BioRender.com/gn3sq1b. (**B**) In vitro qNSC plasma membrane order quantification by Laurdan. SuperPlots showing data from individual cells, as well as the mean of each primary culture ± SEM. *n* = 4 primary cultures. *P* value from Wilcoxon rank-sum test. (**C**) In vitro qNSC plasma membrane order quantification by fluorescence lifetime imaging (FLIM). Left: Schematics of FLIM. Right: Quantification of plasma membrane order by FLIM. *n* = 6 primary cultures from two experiments. *P* value from Wilcoxon rank-sum test. Created in BioRender. lab, Z. (2026) https://BioRender.com/hr7robm. (**D**) In vivo qNSC plasma membrane order assessed by Laurdan. Left: Image from Laurdan rigid wavelength (green) overlaid with GFAP (NSC and astrocyte marker, blue) and EGFR (proliferation marker, purple) staining. Right: Single-plane images from the Z stack enlarged to show verification of qNSCs (top) and images from individual wavelength from a single plane (*Z* = 2) (bottom). qNSCs were identified based on cell-type marker staining, localization, and the presence of basal process. Plasma membrane is outlined in yellow. Scale bar, 10 μm. (**E**) In vivo plasma membrane order quantification. SuperPlots showing data from individual cells, as well as the mean of each mouse brain ± SEM. *n* = 5 mice. *P* value from Wilcoxon rank-sum test. Results from a second independent experiment for (B) and (E) are shown in table S14.

Age-associated changes in plasma membrane lipids could affect several biophysical properties of plasma membranes, including plasma membrane order (i.e., rigidity or fluidity), which is important for barrier function and signaling (*4*, *100*). Unsaturation level, cholesterol, and phospholipid composition all contribute to plasma membrane order (*4*, *100*–*103*). The accumulation of highly unsaturated PUFA side chains in old qNSC plasma membranes (see Fig. 4C and fig. S9F) and the trend toward decreased cholesterol levels in old qNSCs (fig. S9H) suggested that plasma membrane order may decrease during aging in old qNSCs. To experimentally test this prediction and measure plasma membrane order in qNSCs, we used the polarity-sensitive dye Laurdan, which is commonly used to measure plasma membrane order (*104*–*107*) (Fig. 5A). Laurdan can incorporate into membranes and can shift its emission spectrum from ∼450 nm (green, in ordered lipid environment) to ∼500 nm (red, in less ordered lipid environment) (Fig. 5A), and the ratiometric quantification of Laurdan at the plasma membrane provides a measure of membrane order (*107*) (Fig. 5A). We stained young and old qNSCs in vitro with Laurdan and developed an automated image analysis pipeline to segment and outline the plasma membrane of individual qNSCs for unbiased ratiometric quantification (see Materials and Methods for details) (Fig. 5A). Automated ratiometric quantification of Laurdan staining at the plasma membrane revealed that the plasma membranes of old qNSC cultures had a lower normalized generalized polarization ratio (GP ratio), indicative of a lower plasma membrane order, compared to young counterparts (Fig. 5B), consistent with predictions based on lipidomics. We also stratified our automated ratiometric Laurdan quantification based on cell density (fig. S10A) and cell size (fig. S10B), as these factors might influence plasma membrane order. We observed that old qNSCs exhibited a consistent decrease in plasma membrane order, regardless of cell density or size (fig. S10, A and B).

To independently assess plasma membrane order in young and old qNSC cultures, we performed fluorescence lifetime imaging (FLIM) (Fig. 5C). FLIM enables the quantification of fluorescence decay time—a metric that increases when membrane order is higher (*108*, *109*) (Fig. 5C). Our FLIM analysis confirmed that old qNSCs have decreased fluorescence decay time compared to young counterparts (Fig. 5C), regardless of density (fig. S10C), consistent with a lower plasma membrane order. These data corroborate that old qNSCs exhibit lower plasma membrane order in vitro.

We then examined plasma membrane order in qNSCs in vivo using Laurdan staining because this fluorescent dye is compatible with cell marker analysis. To this end, we stained brain sections with the Laurdan fluorescent dye and performed immunostaining with antibodies to cell-type markers to identify qNSCs/astrocytes (GFAP^+^EGFR^−^). For quantification, we only included GFAP^+^EGFR^−^ cells that were immediately adjacent to the ventricular wall and that contained a long basal process that is characteristic to qNSCs to minimize the inclusion of niche astrocytes (Fig. 5D and fig. S10E). We then performed ratiometric quantification of Laurdan fluorescence at the plasma membrane of identified qNSC using an automated pipeline (Fig. 5D and fig. S10D) (see Materials and Methods for details). This analysis of Laurdan staining on brain sections from young and old mice showed that qNSCs from old mice exhibited decreased normalized GP ratio, indicative of a lower plasma membrane order (Fig. 5E), and this decrease in plasma membrane order was observed both around the cell body and at the basal process (fig. S10E). Thus, qNSCs exhibit decreased plasma membrane order with age in vivo, consistent with our in vitro findings.

Together, these results indicate that specific age-related lipidomic changes in qNSCs occur at the level of plasma membrane and are associated with changes in one key biophysical property—a less ordered (more fluid) plasma membrane (though other properties of plasma membranes such as strength, permeability, curvature, membrane potential, and transmembrane protein clustering may also be affected by lipid changes).

### Identification of enzymes that affect the lipidome of young and old qNSCs

**Fig. 6.**
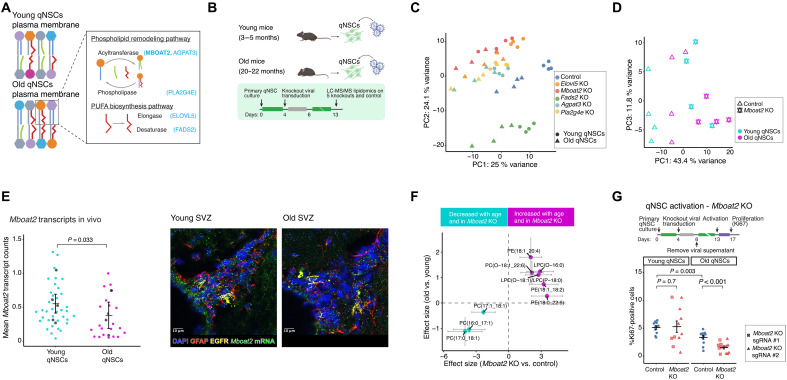
Knocking out *Mboat2* acyltransferase exacerbates impaired old qNSC activation in vitro. (**A**) Overview of enzymes involved in phospholipid remodeling pathway and polyunsaturated fatty acid (PUFA) biosynthesis pathway. Proteins selected for corresponding genetic knockout (KO) are highlighted in blue. Protein selected for overexpression is in bold. (**B**) Experimental setup of in vitro lentiviral-mediated KO followed by lipidomic analysis (same experiment as In vitro #2, Fig. 1A). Created in BioRender. lab, Z. (2026) https://BioRender.com/tmee09y. (**C**) PCA on all lipids. Each symbol represents an individual primary qNSC culture from young or old mice. Cultures were transduced with either a control lentivirus or sgRNAs targeting each gene of interest. (**D**) PCA on all lipids from young and old cultures that are transduced with control lentivirus or sgRNA targeting *Mboat2*. (**E**) *Mboat2* mRNA quantification by in situ hybridization on SVZ qNSCs in vivo. Left: SuperPlots showing quantification from individual tiled images, and the mean of each individual animal ± SEM. *n* = 3 to 5 mice. *P* values from Wilcoxon rank-sum test on quantification of individual tiles. Right: Images showing *Mboat2* transcripts (green) together with GFAP (red), EGFR (yellow), and DAPI staining. Scale bar, 10 μm. (**F**) Lipids change with *Mboat2* KO. Effect size of individual lipids that significantly change between *Mboat2* KO versus control in old qNSCs (*x* axis) and between young and old qNSCs (from Fig. 1E) (*y* axis) is plotted. For each lipid, mean effect size is shown together with SEM (error bar). (**G**) Activation ability of young and old qNSCs with *Mboat2* KO. The percentage of young and old control qNSCs (blue) and qNSCs with *Mboat2* KO (red) that successfully activated was assessed by intracellular FACS analysis for Ki67. Dot and whisker plot from four independent experiments, *n* = 11 to 12 primary cultures. Horizontal bar: mean. Whisker: ± SEM. *P* values from Wilcoxon rank-sum test.

Could enzymes involved in remodeling the lipids that change with age in qNSCs affect the lipidome of these cells, and could they be used to test the functional significance of lipid changes in qNSCs with age? We focused on five lipid metabolic enzymes belonging to two classes known to regulate some of the lipid changes we observed in old qNSCs: (i) phospholipid remodeling enzymes: MBOAT (membrane bound glycerophospholipid O-acyltransferase), AGPAT (1-acylglycerol-3-phosphate O-acyltransferase), and PLA2G4 (phospholipase A2 group IV); and (ii) PUFA biosynthesis enzymes: ELOVL (elongation of very long chain fatty acids-like) and FADS (fatty acid desaturase) (Fig. 6A). Among the different family members for these enzymes, we chose members that satisfy two of these three criteria: (i) high expression in qNSCs/astrocytes based on our single-cell RNA-seq data (*85*), (ii) significant changes in gene expression with age in single-cell RNA-seq, and (iii) substrate specificity of PUFA side chain remodeling in membrane lipid classes (fig. S11) (see Materials and Methods for details). On the basis of these criteria, the five enzyme family members we selected were MBOAT2, AGPAT3, PLA2G4E, ELOVL5, and FADS2.

To determine how manipulating these five enzymes affects the lipidome of young and old qNSCs, we knocked out their respective genes in primary cultures of qNSCs from *n* = 4 young (3 to 5 months) and *n* = 4 old (20 to 22 months) mice using a lentiviral-mediated CRISPR-Cas9 approach (Fig. 6B and table S8). We verified that the knockout of each enzyme gene was efficient, with a frameshift editing of 44 to 69% of the alleles for each gene in both young and old qNSCs (except *Agpat3*) (fig. S12A). We then examined the lipidomic profile of these knockouts by performing LC-MS/MS with 13 standards on young and old qNSCs with respective knockouts and control (same experiment as In vitro #2 used in Fig. 1; see Materials and Methods for details) (tables S1 and S9). We quantified the concentration changes of specific lipids from our lipidomic data and confirmed that the knockouts of *Elovl5*, *Mboat2*, *Fads2*, and *Pla2g4e* in qNSCs led to the expected increase in lipid substrate and/or decrease in product levels (fig. S12, B and C, and table S9) (*Agpat3* knockout did not significantly affect its known substrate and product levels, perhaps due to lower knockout efficiency). Hence, the knockout of most of these enzymes is generally efficient in qNSCs.

We then compared the effects of different enzyme knockouts on the global lipidomic profiles. Each enzyme gene knockout (*Elovl5*, *Fads2*, *Mboat2*, *Agpat3*, or *Pla2g4e* knockout) led to a relatively specific lipidomic profile that could be mostly distinguished in PCA (Fig. 6C). Comparison between different knockouts indicated that more lipid species were uniquely enriched in individual knockouts than shared in two or more knockouts (fig. S12D and table S9). PCA on individual gene knockouts revealed that the cellular lipidome of *Mboat2* and *Agpat3* knockout exhibited a shift toward an “older” lipidome, in both young and old qNSCs (Fig. 6D and fig. S2A). Thus, the knockout of two lipid remodeling enzymes, *Mboat2* and *Agpat3*, shifts the lipidome of qNSCs toward an older lipidome.

### *Mboat2* knockout alters specific lipids and impairs old qNSC function in vitro

We focused on *Mboat2* because *Mboat2* gene expression significantly decreased in old qNSCs/astrocytes in single-cell RNA-seq datasets (fig. S11). In situ RNA hybridization also showed that *Mboat2* expression generally decreased in old brain sections (Fig. 6E).

We determined whether *Mboat2* knockout modulates specific lipids that also change with age. By analyzing our lipidomics datasets, we found that *Mboat2* knockout in old qNSCs led to an increase in several lipid species that normally accumulate with age in qNSCs, notably PUFA-containing PE and ether PC species such as PE(18:0_22:6), PE(18:1_20:4), and PC(O-18:1_22:6) (Fig. 6F). Conversely, *Mboat2* knockout in old qNSCs led to a decrease in several lipid species that normally decrease with age in qNSCs, notably MUFA-containing PC species such as PC(17:0_18:1), PC(16:0_17:1), and PC(17:1_18:1) (Fig. 6F). We also observe that *Mboat2* knockout led to side chain composition changes that mimic age-associated remodeling in old qNSCs. For example, *Mboat2* knockout and aging both led to an increase in PE (20:5) and a decrease in PC(17:1) (fig. S12E).

We asked whether *Mboat2* knockout could have functional consequences on the ability of qNSCs to activate (i.e., transition from quiescence to proliferation)—a main biological function of qNSCs that deteriorates with age (*32*–*34*, *110*, *111*). We knocked out *Mboat2* in young and old qNSCs and measured the proportion of qNSCs that activate in response to growth factors, using FACS analysis with the proliferation marker Ki67 (see fig. S13 for the FACS gating strategy). We verified that qNSC activation ability decreased with age (∼40% decrease, *P =* 0.003) (Fig. 6G). *Mboat2* knockout in old qNSCs led to further impairment in old qNSC activation ability (∼50% decrease, *P* < 0.001) (Fig. 6G). By contrast, *Mboat2* knockout in young qNSCs did not affect their activation level (Fig. 6G), despite affecting the young lipidome (see Fig. 6D)—perhaps because young qNSCs have a better “buffering capacity” for activation than old qNSCs. We next tested whether *Mboat2* knockout affects plasma membrane order. Our Laurdan staining and automated ratiometric quantification pipeline showed that *Mboat2* knockout was not sufficient to affect plasma membrane order in qNSCs (young or old) (fig. S12F). Thus, *Mboat2* deficiency exacerbates the decrease in qNSCs activation with age (but does not overtly affect plasma membrane order).

### *Mboat2* overexpression modulates qNSC lipidome and boosts old qNSC activation in vitro

**Fig. 7.**
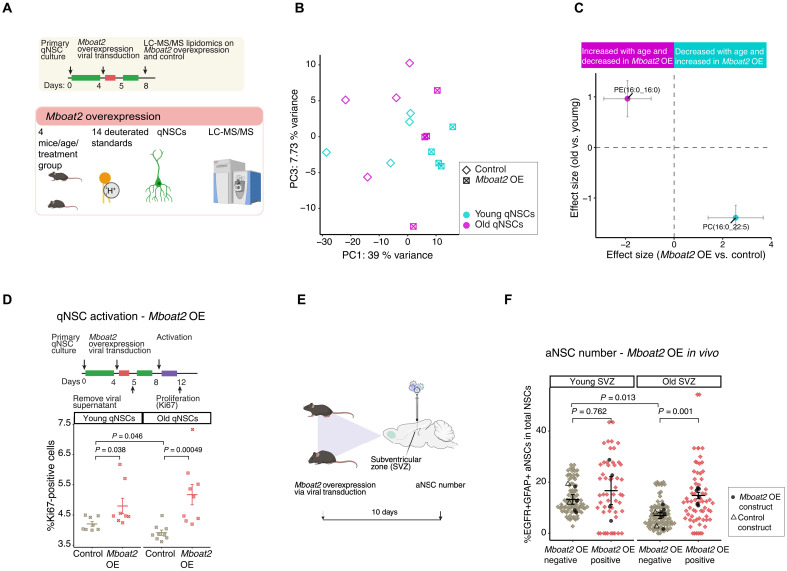
*Mboat2* overexpression boosts the number of activated NSCs in old qNSCs in vitro and in vivo. (**A**) Experimental setup of in vitro *Mboat2* overexpression followed by lipidomic analysis. Number of animals used, number of internal deuterated lipidomic standards used, and neural stem cell type included are indicated. Created in BioRender. lab, Z. (2026) https://BioRender.com/2ujzkut. (**B**) PCA on qNSCs from young and old mice that are transduced with control or *Mboat2* overexpression lentivirus. *n* = 4 primary cultures. (**C**) Lipids change with *Mboat2* overexpression. Effect size of individual lipids that significantly change between *Mboat2* OE versus control in old qNSCs (*x* axis) and between young and old qNSCs (from Fig. 1E) (*y* axis) is plotted. For each lipid, mean effect size is shown together with SEM (error bar). (**D**) Activation ability of young and old qNSCs with *Mboat2* OE. The percentage of young and old control qNSCs (gray) and qNSCs with *Mboat2* OE (red) that successfully activated was assessed by intracellular FACS analysis for Ki67. Dot and whisker plot from two independent experiments, *n* = 8 to 9 primary cultures. Horizontal bar: mean. Whisker: ± SEM. *P* values from Wilcoxon rank-sum test. Data from a third experiment are included in table S14. (**E**) Schematic of *Mboat2* overexpression experiment in vivo. Created in BioRender. lab, Z. (2026) https://BioRender.com/8rrs3bt. (**F**) Activated NSC number with *Mboat2* overexpression in vivo. Percentage of aNSCs in *Mboat2* overexpression negative (gray) and positive cells (red) of young and old mice was quantified. SuperPlots showing data from individual tiled images and the mean of each individual animal ± SEM. *n* = 4 to 8 animals. In the *Mboat2* overexpression negative group, we included two additional animals each in young and old group that were injected with a control lentivirus (see Materials and Methods). *P* values from Wilcoxon rank-sum test on quantification of individual animals.

We next tested the impact of overexpressing *Mboat2* on young and old qNSCs. Lentiviral-mediated overexpression of *Mboat2* led to a fivefold increase in *Mboat2* mRNA level in both young and old qNSCs (with a similar transduction efficiency in young and old of 53 to 55%; fig. S12, G and H). We performed LC-MS/MS with 14 internal standards on *n* = 4 young and *n* = 4 old qNSCs with overexpression of *Mboat2* (see Materials and Methods for details) (Fig. 7A and tables S1 and S12). Analysis of this lipidomic dataset showed the expected decrease and increase in substrate level and product/substrate ratio of MBOAT2 (fig. S12I and table S12). PCA suggests that *Mboat2* overexpression led to a lipidomic shift toward the direction of “young” in both young and old qNSCs (Fig. 7B), opposite to what was observed in cells with *Mboat2* knockout (Fig. 6D).

We asked whether overexpressing *Mboat2* results in changes of lipids that exhibit age-associated differences (Fig. 1E). *Mboat2* overexpression in old qNSCs led to the accumulation of PC(16:0_22:5), a lipid that normally decreases with age. Conversely, *Mboat2* overexpression in old qNSCs led to a decreased level of PE(16:0_16:0), a lipid that normally increases with age (Fig. 7C). At the side chain composition level, *Mboat2* overexpression led to a decrease in three side chain features that contain highly unsaturated fatty acids [PE(20:4), PE(20:5), and PI(20:5)], which normally accumulate with age (fig. S12J). These results suggest that increasing the level of *Mboat2* reverses some of the age-associated lipidomic changes.

We next assessed the effect of *Mboat2* overexpression on the ability of young and old qNSCs to activate in vitro. We verified that qNSC activation ability decreased with age (∼10% decrease, *P* = 0.046) (Fig. 7D). *Mboat2* overexpression significantly boosted the activation of young and old qNSCs (∼9 and 32% increase, *P* = 0.038 and *P* = 0.00049, respectively) (Fig. 7D). These data indicate that *Mboat2* overexpression can boost old NSC function in vitro.

### *Mboat2* overexpression boosts the number of aNSCs in old mice in vivo

What is the impact of *Mboat2* overexpression in vivo? To address this question, we stereotaxically injected lentivirus expressing *Mboat2* in the lateral ventricles of young and old mice, in the vicinity of NSCs (Fig. 7E). We verified that these injections led to increased *Mboat2* transcripts in SVZ cells (including qNSCs) by in situ RNA hybridization on brain sections from young and old mice (fig. S12K). There was a decrease in the number of aNSCs between young and old mice (35% decrease, *P* = 0.013) (Fig. 7F). *Mboat2* overexpression led to a significant increase in the proportion of actively proliferating NSCs (aNSCs) in the SVZ of old mice (but not young mice) in vivo (97% increase, *P* = 0.001) (Fig. 7F). Hence, overexpression of *Mboat2* also boosts the activation potential of old NSCs in vivo.

### Supplementation with plasma membrane lipids from young NSCs

**Fig. 8.**
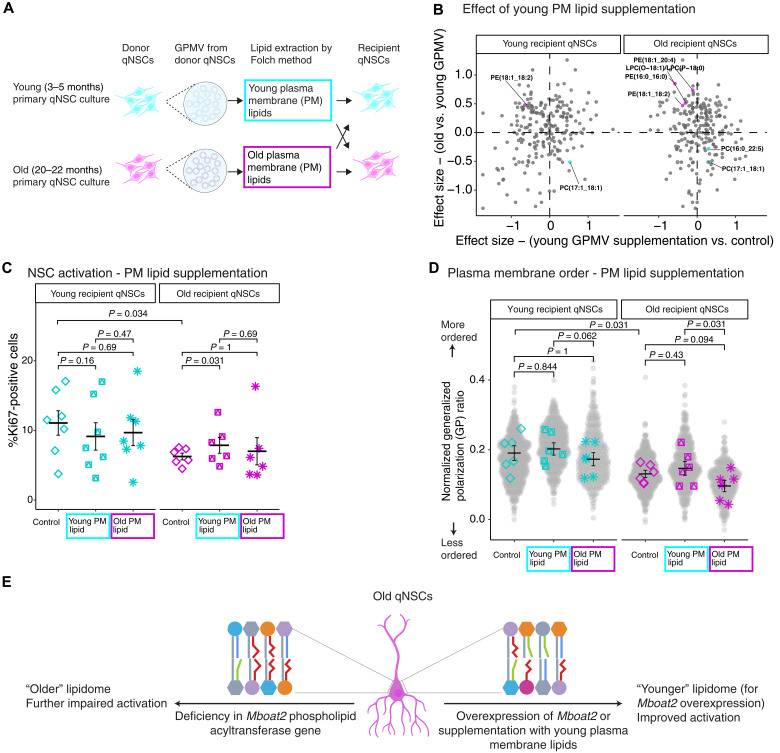
Supplementation with plasma membrane lipids from young cells boosts the activation of old qNSCs. (**A**) Plasma membrane lipids from young or old donor cells were extracted and then supplemented to a different set of recipient cells. Created in BioRender. lab, Z. (2026) https://BioRender.com/q9neb3v. (**B**) Effects of plasma membrane lipid supplementation. Effect size of individual lipids between cells supplemented with young plasma membrane lipids and control recipient cells (*x* axis) is plotted with the effect size between young and old GPMV lipids (from Fig. 4) (*y* axis). Labeled lipids indicate lipids that significantly change with age (Fig. 1) and with *Mboat2* interventions (see Figs. 6 and 7). (**C**) Activation ability of young and old qNSCs with plasma membrane lipid supplementation from young or old donor qNSCs. The percentage of young and old qNSCs that successfully activated was assessed by FACS analysis for Ki67, a proliferation marker. Dot and whisker plot from two independent experiments, *n* = 6 to 7 primary cultures. Horizontal bar: mean. Whisker: ± SEM. Each dot represents a primary culture from an individual mouse. *P* values from Wilcoxon signed-rank test (between paired control and lipid supplementation) and from Wilcoxon rank-sum test between young and old control recipient cells. (**D**) Plasma membrane order of young and old qNSCs with plasma membrane lipid supplementation from young or old donor qNSCs. Plasma membrane order was quantified by Laurdan staining. SuperPlots showing data from individual cells and the mean of each primary culture ± SEM. Results from two independent experiments, *n* = 6 primary cultures. *P* values from Wilcoxon signed-rank test (between paired control and lipid supplementation), and from Wilcoxon rank-sum test between young and old control recipient cells. (**E**) Model for the functional impact of membrane lipids in quiescent neural stem cell aging. Created in BioRender. lab, Z. (2026) https://BioRender.com/ch412we.

To assess the effects of plasma membrane lipids on old qNSC function, we supplemented old qNSCs with lipids extracted from the plasma membrane of young qNSCs. For these supplementation experiments, we first extracted plasma membrane lipids from GPMVs of young or old qNSCs (“donor cells”), and we used these extracted plasma membrane lipids to supplement young and old qNSC cultures (“recipient cells”) (Fig. 8A). Exogenous phospholipids have been shown to incorporate into the plasma membrane via fusion or lipid exchange between lipid vesicles and cells (*112*). Once incorporated, a portion of the exogenous phospholipids can be internalized via the endocytic pathway for subsequent delivery to intracellular compartments (*113*). To verify lipid uptake, we used the lipophilic dye Vybrant DiI to fluorescently label the plasma membrane lipid extracts. We observed that labeled supplemented lipids were found in puncta inside NSCs, suggesting uptake through the endocytic system (fig. S14A, see also below for verification of membrane lipid enrichment).

To examine the global lipidome of young or old qNSCs supplemented with young plasma membrane lipids, we performed LC-MS/MS with 14 internal standards on *n* = 4 young or *n* = 4 old qNSCs supplemented for 72 hours with young plasma membrane lipids (fig. S14B and tables S1 and S13). We verified that the majority of the lipids that increased upon supplementation were indeed plasma membrane lipids (as defined by enrichment in GPMVs versus whole cell extracts) (fig. S14C and table S13), corroborating that the supplementation was successful. We did not observe global changes in recipient qNSCs (young or old) upon young plasma lipid supplementation (fig. S14D). However, old recipient qNSCs supplemented with young plasma membrane lipids exhibited a higher proportion of plasma membrane lipids that are enriched in young GPMVs compared to young recipient counterparts (23.3% in old recipient qNSCs versus 15.1% in young recipient qNSCs) (fig. S14E, yellow slice), including PC(17:1_18:1) and PC(16:0_22:5) (Fig. 8B, right panel, lower right quadrant). Thus, while supplementation with young plasma membrane lipids is not sufficient to affect the global lipidome of old recipient qNSCs, it restores some plasma membrane lipids normally more abundant in young plasma membrane in old qNSCs (and to a greater extent than in young counterparts).

### Supplementation with young plasma membrane lipids boosts old NSC function

We next assessed the effect of plasma membrane lipid supplementation on young and old qNSC activation. As expected, there was a 44% decrease in qNSC activation with age (*P* = 0.034). Notably, supplementation with plasma membrane lipids from young donor qNSCs led to a small, significant increase in the ability of old recipient qNSCs (but not young recipient qNSCs) to activate (23% increase, *P* = 0.031) (Fig. 8C). Supplementation with plasma membrane lipids from old donor qNSCs did not significantly affect the ability of recipient young or old qNSC to activate (Fig. 8C). Hence, supplementation with young plasma membrane lipids can ameliorate the function of old quiescent NSCs (qNSCs).

Last, we determined the effects of plasma membrane lipid supplementation on plasma membrane order by performing Laurdan staining (Fig. 8D). As we have previously found, plasma membrane order was significantly decreased in old qNSCs compared to the young counterpart (*P* = 0.031) (Fig. 8D). Supplementation with young plasma membrane lipids did not significantly affect plasma membrane order in old recipient qNSCs compared to unsupplemented old qNSCs. However, plasma membrane order was significantly higher in old recipient qNSCs supplemented with young plasma membrane lipids than in those supplemented with old plasma membrane lipids (*P* = 0.031) (Fig. 8D). Overall, there was a positive correlation between recipient cells’ ability to activate and their plasma membrane order (fig. S14F). Thus, supplementation with lipids extracted from young plasma membranes improves the function of old qNSCs, and this may be linked to increased membrane order.

## DISCUSSION

Our global lipidomic study shows that several complex lipids change with age in old qNSCs in vitro and in vivo. We find that specific membrane lipids are remodeled with age and exhibit a shift in the side chain composition, including lipids with a higher PUFA content, in qNSCs. Our spatial lipidomic profiling using DESI-MSI indicates that some of these age-related changes in membrane lipids occur in intact neurogenic niches in situ. By performing lipidomic analysis on isolated plasma membrane, we find that plasma membrane lipids contribute to these age-related lipidomic changes. Our study, together with other datasets in different organs (*19*, *20*), highlights lipid changes in cells and organs as a key hallmark of aging.

Prior lipidomics analyses have been conducted in cultured mammalian cells (e.g., oligodendrocytes, neurons, microglia, etc.) (*6*, *74*, *96*, *114*–*119*), but not in the context of aging. In flies, lipidomic analysis indicates lipid changes related to energy metabolism in senescent glia (*81*). Lipidomics studies have also been performed on whole organisms or organs (e.g., whole brains) in aging or Alzheimer’s disease (*8*, *9*, *11*, *12*, *15*, *16*, *19*, *20*, *120*–*131*), but without cellular resolution. Overall, our lipidomics datasets provide a cell-resolved view of the lipidome of aging NSCs in vitro and in vivo.

We observe a reduced plasma membrane order (more fluid) in old qNSCs. Membrane order has been shown to affect a broad range of biological processes, including immune signaling, virus infection, and signal transduction (*1*, *2*, *132*). Reduced membrane order has also been observed in lymphocytes and neutrophils with age (*133*, *134*), whereas increase in membrane order was shown in hippocampal neurons with age (*135*). These different changes in membrane properties with age may be directly linked to the function of the cell, its proliferative status (*136*), and cell-cell contacts (*137*). In old qNSCs, a reduced membrane order may impair activation efficiency through disrupting the localization of transmembrane proteins important for proliferation. Plasma membrane lipids directly interact with a vast number of membrane proteins (*1*, *138*, *139*), including transmembrane receptors, and aging may disproportionally affect membrane proteins (*140*–*143*). Changes in plasma membrane properties may also affect other key aspects of membranes (e.g., strength, permeability, curvature, membrane potential, etc.) that could be important for qNSC function, including endocytosis and phagocytosis.

We identify interventions that affect plasma membrane lipids and find that they regulate the ability of qNSCs to activate during aging (Fig. 8E). For example, deficiency in the phospholipid acyltransferase MBOAT2 exacerbates aging defects in qNSCs and overexpression of MBOAT2 boosts the ability of old qNSCs to activate. As MBOAT2 has been shown to inhibit ferroptosis by remodeling phospholipids in the context of cancer cells (*144*), the beneficial effect of MBOAT2 on old NSC activation could also be due to its ability to decrease ferroptosis. Dysregulation of phospholipid acyltransferases has previously been linked to aberrant intestinal stem cell proliferation (*145*), tumorigenesis (*145*, *146*), and liver disease (*147*–*149*). Our results now implicate a phospholipid acyltransferase in aging. Other enzymes, such as fatty acid elongases (*150*), may also contribute to aging defects. We also show that supplementing young plasma membrane lipids boosts activation in old qNSCs and that plasma membrane order correlates with the ability of qNSCs to activate. Thus, restoring specific lipids to a young lipid profile—either genetically or by affecting lipids themselves—could be an effective strategy to slow cellular aging and counter age-related diseases, including neurodegenerative diseases.

While our study identified specific lipids that changed during NSC aging, we were not able to observe strong changes in lipid class composition. It will be interesting to perform targeted lipidomic studies to determine whether aging is accompanied with changes in subclasses of lipids [e.g., glycolipids (*151*)]. In addition, the total number of lipids we detected from NSCs freshly isolated from the brain was low and the overlap with in vitro NSC cultures was limited. Thus, in addition to increasing sensitivity for lipid detection for in vivo NSCs, it will be important to benchmark and optimize the NSC culture models to better reflect the in vivo conditions. Last, it will be interesting to examine whether overexpression of MBOAT2 or supplementation with lipid membranes in vivo can boost neurogenesis and improve behavior in old mice.

## MATERIALS AND METHODS

### Laboratory animals

Male C57BL/6JN mice (hereafter C57BL/6) obtained from the NIA Aged Rodent colony were used in all experiments. NIA C57BL/6JN mice were habituated for more than 2 weeks at Stanford before use. All mice were housed in the Comparative Medicine and Neuroscience-ChemH building vivarium at Stanford, and their care was monitored by the Veterinary Service Center at Stanford University under IACUC protocol 8661.

### Primary cultures of qNSCs and aNSCs

For primary cultures of mouse NSCs, NSCs were isolated from male C57BL/6 mice as previously described (*32*, *85*, *111*, *152*, *153*). Briefly, SVZs from each brain were microdissected and finely minced. Tissue suspension was then digested for 35 min at 37°C with gentle agitation in Hanks’ balanced salt solution media (Corning, 21-021-CV) containing papain (2 U/ml; Worthington LS003124), Dispase II (1 U/ml; STEMCELL Technologies, 07913), and DNase I (0.1 mg/ml; Sigma-Aldrich, DN25-100mg), and mechanically dissociated. Isolated cells were expanded as neurospheres in culture in “Proliferative NSC media” [NeuroBasal-A medium (Gibco, 10888-022) with penicillin-streptomycin-glutamine diluted 1× (Gibco, 10378-016), 2% B27 minus vitamin A (Gibco, 12587-010), bFGF (20 ng/ml; Peprotech, 100-18B), and EGF (20 ng/ml; Peprotech, AF-100-15)] at 37°C in 5% CO_2_ and 20% O_2_ at 95% humidity.

We then generated qNSCs and aNSCs following published protocol (*154*). The number of plated cells for qNSCs and aNSCs was optimized to ensure similar cell number (1 × 10^6^) at the time of harvesting (see below). We also verified NSC culture purity by immunofluorescence staining with the NSC/astrocyte marker glial fibrillary acidic protein (GFAP).

To generate primary cultures of qNSCs (*154*), tissue culture plates were pretreated with phosphate-buffered saline (PBS; Corning, 21-040-CV) containing poly-d-lysine (50 ng/ml; Sigma-Aldrich, P6407) for 2 hours in a 37°C tissue culture incubator, and then washed three times with PBS before plating cells. Next, 900,000 NSCs (passage 3 to passage 5) were plated in each well of a six-well plate (∼94,000 cells/cm^2^) and were cultured in “Quiescence NSC media” [NeuroBasal-A (Gibco, 10888-022), penicillin-streptomycin-glutamine 1× (Gibco, 10378-016), 2% B27 minus vitamin A (Gibco, 12587-010), bFGF (20 ng/ml; Peprotech, 100-18B), and BMP4 (50 ng/ml; BioLegend, 595302)]. qNSC cultures were incubated in this quiescence media for 7 days, with media change every 2 days before being used for lipidomics experiment. These conditions led to ∼1 × 10^6^ qNSCs at the time of harvesting.

To generate primary cultures of aNSCs, 700,000 NSCs (passage 3 to passage 5) were plated in each well of a six-well plate (∼74,000 cells/cm^2^) pretreated with poly-d-lysine as described above. Cells were then incubated in Proliferative NSC media (see above) for 2 days before being used for lipidomic analysis. These conditions led to ∼1 × 10^6^ aNSCs at the time of harvesting.

### Primary qNSC and aNSC sample preparation for lipidomics

For lipidomics of primary qNSCs and aNSCs, cells were washed twice with PBS before incubating in Quiescence NSC media minus B27 supplement and Proliferative NSC media minus B27 supplement, respectively. Cells were incubated for 3 hours in these media at a 37°C incubator with 5% CO_2_ and 20% oxygen at 95% humidity to remove exogenous lipids contained in B27 supplement. At the end of the incubation period, cells were washed once with PBS (Corning, 21-040-CV) and scraped into 500 μl of ice-cold PBS using cell lifter (Thermo Fisher Scientific 07-200-364). The cell suspension was collected in 2-ml amber glass vials (Thermo Fisher Scientific, 03-FISVA) sealed with polyethylene cap with PTFE/silicone septum (Waters, 186000274). All samples were immediately snap-frozen in liquid nitrogen and stored at −80°C.

### Design of the In vitro #1 lipidomic experiment

The In vitro #1 lipidomic LC-MS/MS experiment was conducted on *n* = 4 to 5 young and *n* = 4 to 5 old primary cultures of qNSCs and aNSCs (each originating from an individual mouse), each at 1 × 10^6^ cells per sample at collection time. A mixture of 14 internal standards was used and added before extraction (see the “Lipid extraction” section below). This mixture consisted of 13 deuterated standards (EquiSPLASH mix, Avanti Polar Lipids, 330731) (1 μl per sample) and Cholesterol (d7) (Avanti Polar Lipids, 700041) (0.2 μl per sample). See table S3 (lipids identified in this lipidomic experiment).

### Lipid extraction

Lipids were extracted from cell suspension thawed on ice using a two-phase, liquid-liquid extraction system—a modified Folch method (*155*). Internal standards were added before extraction. All chemical reagents used were LC-MS grade unless indicated otherwise. Specifically, 300 μl of cold 100% methanol (Thermo Fisher Scientific, A456-500) containing 14 deuterated internal lipid standards was added to the cell suspension. Homogenates were sonicated three times for 30 s each time at room temperature in a water bath sonicator (VWR, 97043-960). Samples were placed on ice for 30 s between each cycle. Following this step, 600 μl of cold chloroform (Acros Organics, AC610281000, stored at −20°C) was added to the homogenates. Samples were then subjected to vigorous vortex at 4°C for 30 min. Biphasic separation was achieved after centrifugation at 3000 rpm for 10 min at 4°C. The lower organic phase containing the lipids was collected and dried down under a nitrogen stream using a TurboVap Classic LV (Biotage) at a flow rate of 0.5 liters/min for 15 min until no visible solution remained and a dried lipid film formed at the bottom. Dried lipids were then resolubilized in 200 μl of 100% methanol at room temperature before moving to −20°C for storage for 1 to 7 days before analysis. On the day of the lipidomic analysis, each sample’s lipid extract was dried down under a nitrogen stream and resolubilized in 200 μl of methanol:toluene (90:10, vol:vol).

### LC-MS/MS lipidomics

Lipidomics workflow, including sample preparation, experimental platform, and detailed lipid identification, and quantification parameters for each lipid class are included in the accompanying reporting checklist (table S1) recommended by the Lipidomics Standard Initiative (LSI) (*156*). Lipid extracts were analyzed by untargeted LC-MS/MS in a randomized order using an Ultimate 3000 RSLC system coupled with a Q Exactive mass spectrometer (Thermo Fisher Scientific) as previously described (*121*). Data from each sample were acquired in positive and negative ionization modes. Lipids were separated using an Accucore C18 column 2.1 × 150 mm, 2.6 μm (Thermo Fisher Scientific, 17126-152130).

A 31-min chromatography gradient with the following mobile phase was used as previously reported (*121*). Specifically, we used a mobile phase of 10 mM ammonium acetate and 0.1% formic acid in 60/40 acetonitrile/water (A) and 10 mM ammonium acetate and 0.1% formic acid in 90/10 isopropanol/acetonitrile (B). The gradient profile used was 30% B for 3 min (0 to 3 min), 30 to 43% in 2 min (3 to 5 min), 43 to 55% B in 0.1 min (5 to 5.1 min), 55 to 65% in 9.9 min (5.1 to 15 min), 65 to 85% B in 6 min (15 to 21 min), 85 to 100% B in 2 min (21 to 23 min), 100% B for 5 min (23 to 28 min), return to 30% B in 0.1 min (28 to 28.1 min), and 30% B for another 2.9 min (28.1 to 31 min). Lipids were eluted from the column at 0.4 ml/min, the oven temperature was set at 45°C, and the injection volume was 5 μl. Autosampler temperature was set at 20°C to prevent lipid aggregation.

Each sample was run in full MS mode. The mass resolution at mass/charge ratio (*m/z*) 200 was 70,000 in full MS mode. Scan range was set at 100 to 1500 *m/*z, with automatic gain control (AGC) target set at 1 × 10^6^ and maximum injection time at 100 ms. MS/MS spectra were acquired on pooled samples on a Q Exactive mass spectrometer equipped with a HESI-II probe and operated in data-dependent acquisition (DDA) mode. DDA tandem mass spectrometry was set with the following parameters: mass resolution at 35,000 and scan range at 200 to 2000 *m/*z, with AGC target set at 1 × 10^5^ and maximum injection time at 50 ms. Minimum AGC target was set at 1 × 10^3^ and intensity threshold was set at 2 × 10^4^. Loop count and TopN were both set at 10. A stepped normalized collision energy was set at 25 and 30%. Dynamic exclusion was set at 12 s. Mass window for precursor ion isolation was set at 1 Da.

### Quality control

Data quality was ensured by (i) injecting pooled sample with deuterated standards six times to equilibrate the LC-MS system before running the sequence; (ii) checking mass accuracy, retention time, and peak shape of deuterated standards in each injection of pooled sample; and (iii) ensuring that pooled samples with deuterated standards were injected after every 10 to 15 sample acquisitions throughout the entire experiment to ensure the stability of the instrument. Precision of the method was evaluated by calculating the coefficient of variation (CV) of all deuterated standards from pooled samples after every 10 to 15 sample acquisitions throughout each experiment. Calculated CVs of all internal standards in reported studies were <10%. Retention time drift was monitored by plotting the retention time of internal standards in pooled samples in the order of acquisition. Calculated retention time drift was between 0.1 and 0.15 min.

### Lipid identification

MS-DIAL version 5.5.250221 software was used for deconvolution, peak picking, alignment, and compound identification (*157*). The parameter settings were as follows: MS1 tolerance, 0.01 Da; MS2 tolerance, 0.025 Da; minimum peak height, 1000 amplitude; mass slice width, 0.05 Da; smoothing method, linear weighted moving average; smoothing level, three scans; minimum peak width, five scans. Positive ion mode adducts included the following: [M + H]^+^, [M + NH_4_]^+^, [M + Na]^+^, and [M + H-H_2_O]^+^. Negative ion mode adducts included the following: [M-H]^−^, [M + HCOO]^−^, and [M + CH_3_COO]^−^. Although both ammonium acetate and formic acid were used in our mobile phase, we predominantly used formate adduct [M + HCOO]^−^ for lipid identification as it is more stable and provides cleaner spectra for identifying complex lipid classes. Similar observations were made in another study using the same mobile phase modifiers (*158*). Only metabolic features present in >^2^/_3_ of all samples were kept for further analysis. Lipids were identified by matching the precursor ion mass and tandem mass spectra to a spectral library included in MS-DIAL (Msp20250218112233_NCDK_conventional_converted_dev) with settings listed above.

All identified lipids were reported at the molecular species level. The most abundant ion adduct was selected for each lipid class for downstream analysis and quantification. Specifically, in positive mode, [M + H]^+^ for lysophosphatidylcholine (LPC), PC, PE, and sphingomyelin (SM); [M + NH_4_]^+^ for monoacylglycerol (MG), diacylglycerol (DG), and triacylglycerol (TG); and [M + H-H_2_O]^+^ for ceramide (Cer) and cholesterol. In negative mode, [M-H]^−^ for LPE, PI, PS, and phosphatidylglycerol (PG). All reported lipids were quantified at level 2 according to the LSI guideline.

To reduce the risk of misidentification, MS/MS spectra from all lipids were manually investigated to validate lipid annotation. See table S2 for spectra ions used in the manual validation of all lipid classes. The manual validation consisted in verifying that (i) both positive and negative mode MS/MS spectra matched the expected fragments, (ii) the main lipid adduct forms detected in positive and negative modes were in an agreement with the lipid class identified, (iii) the retention time was compatible with the lipid class identified, (iv) the peak shape shows symmetrical, Gaussian pattern, and (v) the fragmentation pattern of each lipid class detected was experimentally validated using lipid internal standards. Accurate mass and MS/MS spectra from pooled samples were used for lipid identification and manual validation. Lipid species from individual samples were annotated based on accurate mass and retention time alignment to validated lipids identified from pooled samples.

### Lipid quantification

Endogenous lipids were reported in molar concentrations based on the intensity ratio between endogenous lipids and the deuterated lipid standard within the same lipid class with known concentration. The inclusion of 14 internal standards from the EquiSPLASH deuterated lipid standard mix (see above) allowed us to obtain relative quantification in molar concentration in 14 lipid classes (PC, LPC, PE, LPE, PG, PI, PS, TG, DG, MG, ChE, Cer, SM, and cholesterol). As all samples have a similar number of input cells (1 × 10^6^ cells for both qNSCs and aNSCs at the time of harvesting) (see the “Primary cultures of qNSCs and aNSCs” section), each lipid concentration was normalized using the median lipid molar concentration (instead of total or average concentration to reduce sensitivity to outlier high-abundance lipids) from all lipids of each sample. Before the lipidomic quantification, we conducted an optimization experiment on a mix from young and old qNSC and aNSC samples (to represent sample matrices) with a serial dilution of EquiSPLASH and Cholesterol (d7) deuterated standards spanning five orders of magnitude to ensure that the introduced concentration of all standards were within the linear detection range of endogenous lipids in qNSCs and aNSCs.

### Reporting

Raw lipidomic data were uploaded to Metabolomicsworkbench.org under StudyID ST002258, ST002259, ST002260, ST004116, and ST004117. The metadata and the summary of quantification and identification data are all available for download.

### Nomenclature

Lipid nomenclature used followed the guideline of LIPID MAPS (*159*). The number of C═C bonds was indicated by the number after the carbon number in the acyl chain and was separated by “:”. The separator “_” was used between the two acyl chains if sn-position of the fatty acids was not known. The separator “/” was used when sn-position of fatty acids was proven (e.g., sn-1/sn-2 or sn-2/sn-3 for glycerophospholipids). Using our mass spectrometry setup, alkyl-linked LPE and LPC species [e.g., LPE(O-18:1)] exhibited the same tandem mass spectra as alkenyl-linked LPE and LPC species with one less double bond [e.g., LPE(P-18:0)], as previously reported (*160*). For those species, both annotations are listed and separated by “/” [e.g., LPE(O-18:1)/LPE(P-18:0)]. To distinguish between isomeric lipids, lipids with identical molecular formulas but different structures, an identifier (a, b, or c) was added at the end of each lipid annotation. Only isomer lipids within the same retention time window of (±0.1 min) across different experiments were considered to have the same identification and included in cross-study experiments.

### Design of In vitro #2 lipidomic experiment

The In vitro #2 lipidomic LC-MS/MS experiment was conducted on *n* = 4 young and *n* = 4 old primary cultures of qNSCs, each originating from an individual mouse, for a total of 1 × 10^6^ cells per sample at collection time. In this experiment, qNSCs were transduced with control lentivirus or lentivirus expressing sgRNA targeting genes of interest (*Elovl5*, *Mboat2*, *Agpat3*, *Pla2g4e*, and *Fads2*). See the “Lipidomic dataset of CRISPR-Cas9–mediated knockouts in vitro of *Mboat2*, *Elovl5*, *Agpat3*, *Fads2*, and *Pla2g4e* in young and old qNSCs” section below. This experiment was used in both Figs. 1 (control) and 6 (control versus lipid enzyme gene knockouts). See table S1 (internal standards) and tables S3 and S9 (lipids identified in this lipidomic experiment). This experiment was conducted as described above for “In vitro lipidomic experiment,” with the following modifications:

1. For standards, we included a mixture containing the same 13 deuterated standards (EquiSPLASH mix, Avanti Polar Lipids, 330731), but we also included a deuterated oleic acid standard (Cayman Chemical, 9000432) (1 μl per sample) and no cholesterol standard. We refer to it as “13 lipid classes.”

2. For this experiment, each sample was run in full MS mode and MS/MS spectra were acquired in individual samples (instead of in pooled samples as in all other studies) on a Q Exactive mass spectrometer equipped with a HESI-II probe and operated in data-dependent acquisition mode.

3. Free fatty acids were analyzed in a randomized order using an Ultimate 3000 RSLC system coupled with a Q Exactive mass spectrometer (Thermo Fisher Scientific). The Q Exactive was operated in data-dependent acquisition mode in negative ionization mode. Free fatty acids were separated on a Zorbax StableBond aq column (dimension: 2.1 mm by 50 mm, particle size: 1.8 μm) (Agilent Technologies, 827700-914) at 0.6 ml/min using a mobile phase solvent consisted of 0.06% acetic acid in water (A) and 0.06% acetic acid in acetonitrile (B). The gradient was set with 1 to 90%B in 9 min, the oven temperature was set at 60°C, the autosampler temperature was set at 4°C, and the injection volume was 15 μl. Single-point internal standard calibration with deuterated oleic acid (Cayman Chemical, 9000432) was used to estimate absolute concentrations for all free fatty acids in this experiment.

### Overlapping lipids between in vitro lipidomic datasets

Overlapping detected lipids between in vitro lipidomic datasets were identified using the annotated molecule identification (see the “Lipid identification” section for details). A lipid species was labeled as “overlapping” if it was detected in independent datasets. A Venn diagram was drawn with the size of each area proportional to the number of lipids using the function “euler” with the R package “eulerr” v7.0.2.

To determine the abundance of overlapping detected lipids based on molar concentration, we calculated the percentage of molar concentration of commonly identified lipids (summing the concentration of overlapping lipids and dividing this sum by the total lipid concentration of each sample) as well as of lipids that were uniquely detected in individual datasets. We presented these percentages of molar concentration of lipids using a stacked bar plot.

We also assessed overlapping lipids that show the same direction of age-related changes. Lipids from the different datasets were first divided into “up with age” or “down with age” groups based on the age-related log2 fold change, calculated as the log2-transformed ratio between the mean lipid concentration of old versus young samples. Lipids that are categorized as “up with age” or “down with age” have a positive or negative age-related log2 fold change, respectively. Overlapping lipids were then identified as described above from the “up with age” or “down with age” groups. All overlapping lipids are included in table S4.

There was some variability between in vitro datasets, for detected lipids and for age-related changes. Some of the variability could be biological and originate from the fact that experiments were conducted with primary cells and with different cohorts of aging mice. The variability could also be technical and due to differences in design (i.e., presence or absence of lentiviral infection), the fact that experiments were run at different times, and differences in the LC-MS/MS analysis (see above).

### Comparison of lipid concentrations between in vitro lipidomic datasets

To compare lipid concentrations detected across both in vitro lipidomic datasets, we first identified overlapping lipids that were identified in these datasets (described above). We then plotted the normalized lipid concentration of each individual lipid from each dataset. Correlation coefficients and *P* values were obtained from Pearson correlation analysis in R version 4.4.2.

### PCA of lipidomic datasets

PCA was done on lipids with quantified concentration using log2-transformed normalized lipid concentration. PCA was generated using the function “prcomp()” with R version 4.4.2. All PCA plots were made to visualize samples on principal component 1 (PC1) and principal component 2 (PC2) or principal component 3 (PC3). The differences between young and old qNSCs were smaller than the differences between cell states (quiescent and activated), similar to what has been observed for other molecular features (*32*, *85*, *153*).

PCA on combined samples from In vitro #1 and In vitro #2 was performed on the 175 overlapping lipids that were detected in both datasets (see the “Overlapping lipids between in vitro lipidomic datasets” section). *z*-score—a numerical value that indicates how many standard deviations a data point is away from the mean of a distribution—was calculated for each lipid based on the log2-transformed normalized concentration of both studies.

### Heatmap visualization of lipids with age-related changes in vitro

Lipids with age-related changes in qNSCs were identified by a Wilcoxon rank sum test, followed by multiple hypothesis correction using FDR (Benjamini-Hochberg correction for multiple comparisons). Lipids that change with age with FDR-adjusted *P* < 0.1 were included in the In vitro #1 and In vitro #2 heatmaps. Heatmaps were generated from differential lipids with Pearson correlation using the R package “ComplexHeatmap” v2.22.0.

### Analysis of the magnitude of age-related changes in two independent in vitro lipidomic experiments by calculating “effect size”

To analyze the magnitude of the changes with age across two independent in vitro lipidomic datasets, we used “effect size” to calculate the difference between young and old samples. Effect size is a stringent metric that integrates both fold change (numerator of the calculation) and variability within datasets (denominator of the calculation). Calculating effect size enables comparison across independent experiments, and it has been previously used for lipidomics and metabolomics (*82*–*84*). Hedges’ *g* statistics was used to evaluate the standardized mean difference between two groups (e.g., young versus old) for a given measurement (*161*, *162*). Specifically, if one group is 1 (e.g., old) and the other group is 2 (e.g., young)

$$g={\text{correction factor}}^{*}\;\left[({\text{Mean}}_{1}-{\text{Mean}}_{2}\right)/\text{SD\_weighted\_pooled]}$$

where

$$\text{SD\_weighted\_pooled}=\text{sqrt}\{\left[\left(n_{1}-1\right)*{{\text{SD}}_{1}}^{2}+\left(n_{2}-1\right)*{{\text{SD}}_{2}}^{2}\right]/\left(n_{1}+n_{2}-2)\right\}$$

A correction factor was applied to the experiment with a small sample size (<50 samples).

$$\text{correction factor}=1-3/\left[4^{*}\left(n_{1}+n_{2}\right)-9\right]$$

Throughout our study, positive values in age-related effect size calculation represented features that increased with age, and negative values represented features that decreased with age. We note that differences with age within a cell type are typically not large, and smaller than changes between cell types.

### Plotting effect size means and SEM and confidence intervals of specific lipids

Age-related effect sizes of individual lipids from each experiment (*g*) were used to calculate the mean effect size and the standard error of the mean (SEM). The mean effect sizes across both in vitro lipidomic datasets, individual effect sizes from each dataset, and the SEM were visualized using forest plots. Next, the 95% confidence interval was calculated as the range from (mean effect size − 1.96 * SEM) to (mean effect size +1.96 * SEM). Statistical significance was assessed based on the *P* value from test statistics using the 95% confidence interval of the data, and multiple hypothesis correction was done by FDR (Benjamini-Hochberg correction for multiple comparisons). Lipids that changed with age with FDR-adjusted *P* < 0.05 were noted to be significant across both experiments.

### Abundance of lipids with significant age-associated changes

Lipids were ranked based on the normalized concentration. Lipids that showed significant age-related changes in In vitro #1 and In vitro #2 lipidomic experiments were highlighted. Lipids that significantly changed with age spanned a range of concentration and included high abundance lipids. We note that the changes with age in low-abundance lipids should be taken with caution.

### Side chain composition analysis

We used the lipid class followed by side chain carbon and double bond number to indicate the composition of a given lipid class containing a specific side chain. We considered each lipid class/side chain combination as a side chain composition feature, e.g., PE(20:4). For each individual lipid class, we grouped lipids based on the presence of each unique side chain within a given class and calculated the total concentration (micromolar) of each side chain composition feature. Alkyl-linked LPE/LPC species and the corresponding alkenyl-linked LPE/LPC species with one less double bond were excluded from this analysis, owing to multiple possible side chain annotations (see the “Nomenclature” section). Normalized lipid concentration was used for In vitro #1 (which had 14 internal standards) and In vitro #2 (which had 13 internal standards).

### Comparison of molar percentage of side chain composition between two datasets

To compare side chain composition detected in the two in vitro lipidomic datasets, we first identified overlapping side chain composition features (described above) in both datasets. We then plotted the molar concentration of each side chain feature from each dataset. Correlation coefficients and *P* values were obtained from Pearson correlation analysis in R version 4.4.2.

### Plotting effect size means and SEM and confidence intervals of specific side chain features

Age-related effect sizes of side chain features from each experiment (*g*) were used to calculate the mean effect size and the SEM. The mean effect sizes across both in vitro lipidomic datasets, individual effect sizes from each dataset, and the SEM were visualized using forest plots. Next, 95% confidence interval was determined as the range from (mean effect size − 1.96 * SEM) to (mean effect size + 1.96 * SEM). Statistical significance was assessed based on the *P* value from test statistics using the 95% confidence interval of the data, and multiple hypothesis correction was done by FDR (Benjamini-Hochberg correction for multiple comparisons). Side chain features that changed with age with FDR-adjusted *P* < 0.05 were noted to be significant across both experiments.

### Abundance of side chain composition features with significant age-associated changes

Features were ranked based on molar concentration of each feature across all lipid classes. Features that showed significant age-related changes in In vitro #1 and In vitro #2 lipidomic experiments were highlighted. The side chain composition features that showed significant changes with age included features that spanned a range of molar concentrations within each experiment.

### Design of “in vivo” qNSC lipidomic experiment

To isolate qNSCs directly from mouse brain, we used FACS (*32*, *85*). This experiment was performed on *n* = 6 young and *n* = 6 old qNSC samples. For each of these six samples, we pooled five young mice or five old mice to obtain ∼7500 to 9000 freshly isolated qNSCs for each biological replicate. Briefly, the SVZs of young (3 to 5 months old) and old (20 to 22 months old) C57BL/6 male mice were microdissected following a previously published protocol (*86*). Single-cell suspension was obtained and then stained with 1:50 CD31 (BioLegend, 102405), 1:50 CD45 (BioLegend, 103108), 1:50 CD24 (BioLegend, 101805), 1:300 Prominin-1-biotin (Thermo Fisher Scientific, 13-1331-82), and 1:300 EGF-Alexa 647 (Molecular Probes, E35351) for 30 min on ice. Cells were then stained with 1:1000 Streptavidin-PEcy7 (eBioscience, 25-4317) for 30 min on ice. qNSCs [CD31^−^CD45^−^CD24^−^Prominin-1(CD133)^+^EGFR^−^] were sorted. All FACS was performed at the Stanford FACS facility on a BD Aria sorter, using a 100-μm nozzle at a pressure of 13 psi (0.896 bar). We note that the tandem dye PE-Cy7 we used (conjugated to Prominin-1 antibody) has a broad emission spectrum and extends into the far-red range (∼660 nm), which overlaps with the AF647 (EGF) detection channel even though they are excited by different lasers. Correlated signal between PE-Cy7 and AF647 still persisted even after spectral compensation was applied. Signal correlation between PE-Cy7–conjugated Prominin-1 and AF647-conjugated EGF was also observed, albeit in a less pronounced manner, in previous studies (*32*, *86*). The greater spillover we observed in our experiments may be due to the fact that we used fluorescein isothiocyanate (FITC) for all three lineage-specific markers (CD45, CD31, and CD24), whereas prior studies used a distinct fluorophore for each marker (*32*, *86*). Because FITC and PE-Cy7 are excited by the same 488-nm laser on the BD Aria sorter, FITC emission can spill into the PE-Cy7 detector, which may exacerbate the correlated signal between PE-Cy7 and AF647. We also note that all compensations were done on single-color staining controls of SVZ cells, and we then used FMO (fluorescence minus one) controls to take into account the residual spectral spillover and inform us on the gating of populations of interests. qNSCs of each sample (pooled from five young or old mice) were sorted into 500 μl of ice-cold PBS, and the cell suspension was immediately snap-frozen in liquid nitrogen and then transferred to a −80°C freezer for storage.

As described above, a mixture containing 13 deuterated standards (EquiSPLASH mix, Avanti Polar Lipids, 330731) was added to each sample (0.5 μl per sample), together with 0.1 μl per sample of Cholesterol (d7) (Avanti Polar Lipids, 700041) (table S1). This experiment followed the exact same procedures as outlined for In vitro #1 lipidomics. The low number of cells for the in vivo lipidomic dataset led to a smaller number of total detected lipids, and thereby fewer significant lipids between young and old. For the in vivo lipidomic heatmap, all identified lipids were included (no multiple hypothesis correction).

Overlapping lipids between in vitro and in vivo experiments were evaluated as described above for different in vitro lipidomic experiments (we used “In vitro #1” for the overlap with “In vivo”). We note that the low overlap between in vivo and in vitro lipidomic studies likely originate from the low cell number in in vivo experiments. In addition, in vitro qNSC cultures might not recapitulate all features of freshly isolated qNSCs (especially non–cell-intrinsic or compensatory features).

### Identification of lipid and side chain composition changes with age in qNSCs in vitro and in vivo

Effect sizes with age from lipidomic data of In vitro #1 and In vivo were used to compute the mean overall effect size and the SEM from in vitro and in vivo studies. Next, 95% confidence interval was determined as the range from (mean effect size − 1.96 * SEM) to (mean effect size +1.96 * SEM). Statistical significance was assessed based on the *P* value from test statistics using the 95% confidence interval of the data, and multiple hypothesis correction was done by FDR (Benjamini-Hochberg correction for multiple comparisons). Lipids and side chain features that change with age with FDR-adjusted *P* < 0.05 were noted to be significant across in vitro and in vivo studies.

### SVZ sample preparation for DESI-MSI

For lipidomics in situ (in the context of intact tissue), we used DESI-MSI. For these experiments, SVZ neurogenic niches were microdissected from *n* = 7 young (3 to 5 months old) and *n* = 7 old (20 to 22 months old) C57BL/6 male mice brains. Each microdissected SVZ niche was oriented on a cryomold (Thermo Fisher Scientific, NC9511236) such that the lateral wall of the SVZ faced down to flatten the tissue, and then each sample was immediately snap-frozen on dry ice. For cutting, SVZs were not embedded in optimal cutting temperature (OCT) polymeric compound to avoid background compound peaks from residual OCT during tissue sectioning. Instead, a minimal amount of OCT was applied to attach the caudoputamen, which is adjacent to the lateral wall of the SVZ, to the mounting plate before sectioning. Unfixed SVZs were cut into 16-μm sections parallel to the lateral wall of the SVZ at −20°C on a Leica CM3050S cryostat. Sections were thaw-mounted onto slides (Thermo Fisher Scientific, 12-550-15) without fixation, before transferring to a −80°C freezer for storage. Tissue slides were dried for 20 min at room temperature using a vacuum desiccator (Thermo Fisher Scientific, 5311-0250) before DESI-MSI experiments.

### Desorption electrospray ionization mass spectrometry imaging

We used DESI-MSI to determine whether some of the lipid changes with age were also observed in situ, in intact neurogenic niches. For DESI-MSI, we used a procedure that we previously published (*89*) on sections of SVZ whole mount to assess cell layers that are immediately adjacent to the ventricle, where a high density of NSCs reside. Briefly, a custom-built DESI imaging stage was coupled to a hybrid LTQ-Orbitrap mass spectrometer (Thermo Fisher Scientific) for DESI-MSI. The mass spectra were acquired in the negative ion mode from *m/z* 50 to 1000 using the Orbitrap mass analyzer with the resolving power set at 60,000. The spray voltage was set to −3.5 kV, and the capillary voltage was set to −65 V. The tube lens voltage was set to −120 V. Ion injection time was 100 ms, and one microscan was performed. The solvent system used was *N*,*N*-dimethylformamide/acetonitrile = 1:1 (vol/vol) and was provided at a flow rate of 0.8 μl/min. Assisted by a nebulizing gas (N_2_) at a pressure of 150 psi (10.342 bar), molecules of interest from tissues are desorbed and ionized. DESI spray tip-to-surface distance was 2 mm; spray incident angle was 56°, and spray-to-inlet distance was 6.5 μm. Step size in the moving stage was set to 200 μm, and automatic gain control of mass spectrometer was switched off. These parameters were empirically found to yield the optimal MS signal from brain tissues. Tandem mass spectra were obtained using collision-induced dissociation from the ion trap mass analyzer to validate lipid structures based on their characteristic fragment ions. All experiments were carried out under identical experimental conditions to allow comparison between measurements.

### Immunofluorescence staining and imaging for DESI-MSI

To gain cellular resolution, we performed immunofluorescence staining on the exact same sections used for DESI-MSI. After each DESI scan, tissue sections were fixed with 4% paraformaldehyde (Electron Microscopy Science, 15714) diluted in PBS for 20 min at −20°C. Antibodies against GFAP (Abcam, ab53554, RRID:AB_880202) were used at 1:1000 dilution, and antibodies against Ki67 (eBioscience, SolA15, RRID:AB_10854564) were used at 1:500 dilution at 4°C overnight. Donkey anti-goat antibodies conjugated with Alexa Fluor 647 (Thermo Fisher Scientific, A-21447, RRID:AB_141844) and donkey anti-rat antibodies with Alexa Fluor 488 (Thermo Fisher Scientific, A-21208, RRID:AB_2535794) were used at 1:250 dilution at room temperature for 1.5 hours for a secondary staining. All sections were mounted with 50 μl of ProLong Gold Antifade mounting media containing a fluorescent nucleic acid dye 4′,6-diamidino-2-phenylindole (DAPI) (Thermo Fisher Scientific, P36931) before imaging. Confocal microscopy was carried out using a Nikon Eclipse Ti confocal microscope equipped with a Zyla sCMOS camera (Andor) and NIS-Elements software (AR 4.30.02, 64-bit) using a 10× objective. A tiled image covering the entire area of SVZ was taken for each mouse.

### Image quantification for DESI-MSI

To quantify the immunofluorescence images to obtain cell-type composition for downstream lipidomic deconvolution, we used a Julia language script, as we have previously reported in our previous publication (*89*). Briefly, detection of nuclei was performed for immunofluorescence-stained regions of tissue using DAPI as a nuclear marker and blob detection algorithms provided by the Julia package Images.jl. After nuclei detection, cells were classified as GFAP-positive and Ki67-positive (aNSCs), GFAP-positive and Ki67-negative (qNSCs/astrocytes), GFAP-negative and Ki67-positive (NPCs), and GFAP-negative and Ki67-negative (likely neurons), based on the staining in the region immediately surrounding the detected nuclei. To threshold between these binary designations, we used Otsu’s method (*163*) on the summed staining intensity for a given channel by cell. After each cell was classified and binned into a category based on the calculated threshold, each region’s proportion of cell types was calculated and presented as a percentage of total cells in that region for downstream analysis.

### Cell type–specific deconvolution of DESI-MSI mass spectra

To perform cell type–specific deconvolution of DESI-MSI mass spectra, we used a similar method to that we previously published to obtain astrocyte- and neuron-specific lipidomic profiles in brain sections with cell heterogeneity, validating it with signature metabolites that are known to be characteristic to each cell type (*89*). DESI-MSI scans of the SVZ have a resolution of 200 μm by 200 μm, and these areas in general contain a mixed population of 150 to 250 cells (qNSCs, aNSCs, NPCs, astrocytes, and other cell types). For cell type–specific deconvolution, peak intensity of each individual DESI scan was normalized by dividing each peak by the intensity value of an exogenous standard (4-methoxybenzoic acid) introduced by the solvent system. We then aggregated all normalized peak intensities by rounding their *m/z* value by its first significant digit to reduce data sparsity before deconvolution. We estimated cell type–specific intensity values using the csSAM package (version 1.2.4) (*93*) by pairing normalized DESI data with cellular proportions quantified by immunofluorescent staining on the same sample of the four cell types (qNSCs/astrocytes, aNSCs, NPCs, and likely neurons), as described in the section above. Specifically, we grouped all scans obtained from the same animal to estimate mean and SEM across all peaks for each mouse. We then used cell type–specific mean intensities for statistical comparisons and downstream analyses between SVZ niches, each from an individual mouse. Fold change differences between old and young mice were estimated by computing Hedges’ *g* effect size (described above) using age groups as class labels.

### Evaluation of cell type–specific lipidomic deconvolution accuracy

To validate the accuracy of cell type–specific deconvolution by csSAM, we generated a reconstituted in silico mixture lipidomic dataset. To mimic the four-way deconvolution applied to our DESI-MSI data, we used lipidomic data from four different cell types: qNSCs and aNSCs (from In vitro #1 lipidomic dataset) as well as neurons and oligodendrocytes from an untargeted lipidomic data from a previously published paper (*6*). We then reconstituted mixture lipidomic data to simulate our DESI-MSI data samples. Specifically, we grouped one spectra from each cell type to generate a unique in silico lipidomic profile representing an individual mouse. We then created 10 mixtures for each mouse by combining the spectra of four cell types using a different vector of cell-type proportion for each mixture. We performed deconvolution on these reconstituted mixture lipidomic data and obtained estimated mean levels of cell type–specific metabolite intensity for each individual mouse. Last, we compared mean estimated metabolite intensity after deconvolution to the measured metabolite intensity before mixing by Pearson correlation. As a negative control, we also performed deconvolution using the same set of reconstituted in silico mixture but with mismatched cell-type proportions instead. Specifically, we performed deconvolution with randomly generated cell-type compositions from 100 permutation experiments. We then analyzed the distribution of the Pearson correlation coefficient between the measured and estimated lipid intensity for each cell type of each individual mouse after deconvolution.

### OPLS-DA and assessment of model performance

OPLS-DA is a supervised statistical method used to analyze multivariate data with the goal of identifying differences between predefined groups (*95*). This supervised dimensionality reduction model is often used in metabolomics because it is well suited to handle highly correlated metabolites (e.g., metabolites in the same pathway) (*95*). After obtaining cell type–specific lipidomic data from each individual mouse by DESI-MSI followed by in silico deconvolution, we performed OPLS-DA to identify cell type–specific lipidomic changes between qNSCs/astrocytes and aNSCs, and in a separate analysis, between young and old qNSCs/astrocytes in situ. We used the “opls” function from the ropls package (version 1.22.0) with the following parameters: predI = 1, orthoI = 1, crossvalI = 6, permI = 100, and scaleC = “standard”. The OPLS-DA model was visualized by plotting each individual data point using the predictive score that captures the variation in the data that is correlated with sample classification (cell type or age) and the orthogonal predictive score that captures the variation in the data that is uncorrelated with sample classification (i.e., structured noise).

Model predictability was assessed by performing 100 permutation tests with sample classification randomly assigned. The predictability (*Q*^2^) was plotted, and the *P* value was calculated between the original and permutated models. Low *Q*^2^ in permuted models, in combination with a significant *P* value, indicates that the original model is significantly better than permuted models in identifying features that are predictive in separating samples of different classes. We observed that this was the case for the OPLS-DA model to distinguish qNSCs/astrocytes versus aNSCs (fig. S7D), validating that our data acquired on DESI-MSI and the accompanying analysis pipeline are able to detect cell type–specific changes. However, the OPLS-DA model to distinguish young versus old qNSCs/astrocytes did not have a lower *Q*^2^ for permuted models or a significant *P* value (fig. S7F), suggesting that this model, while able to distinguish young and old qNSCs/astrocytes, was overfitted and therefore not reliable for global analysis (though analysis of specific lipids is still feasible).

### Quantification on specific lipids from DESI-MSI data in a spatially defined manner

The SVZ neurogenic niche exhibits spatial heterogeneity, with qNSCs being localized in the rostral region (*90*–*92*) (Fig. 3E). To gain a spatially defined view of the SVZ, we obtained a large rectangular area containing all scans covering the entire SVZ area (with minimal numbers of scans without tissue coverage) for each individual SVZ (Fig. 3E). We then divided this large rectangular area into three equally sized smaller rectangles along the rostral-caudal axis of the SVZ (Fig. 3E). We then quantified the total normalized intensity of a given lipid from all scans of each smaller rectangle. The lipid intensity represents the metabolite abundance from all cells (not just qNSCs) contained in the scan.

### GPMV generation and design of the GPMV lipidomic experiment

To determine whether plasma membrane lipids contributed to complex lipid changes with age, we performed lipidomics on GPMVs, which are mostly made of plasma membranes (without internal organelles) (*98*, *164*). GPMVs were generated from primary qNSC cultures from *n* = 8 young or *n* = 8 old mice (each culture originating from one mouse), following a previously described protocol (*98*, *164*). Specifically, 13 × 10^6^ qNSCs (passage 5 to passage 6) were plated in a 15-cm plate (∼90,000 cells/cm^2^) and quiescence was induced for 7 days (as described above in the “Primary cultures of qNSCs and aNSCs” section). Cells were washed twice with “GPMV vesiculation buffer” [150 mM NaCl, 2 mM CaCl_2_, and 20 mM Hepes (Sigma-Aldrich, 54457) in water, pH 7.4] at 37°C. GPMV generation was then induced by adding 2 mM of *N*-ethylmaleimide (Sigma-Aldrich, 04259) for 1.5 hours in a 37°C incubator with 5% CO_2_ and 20% O_2_, with gentle agitation every 30 min. At the end of the incubation, the cell supernatants (containing GPMVs) were first subjected to a low-speed centrifugation at 200*g* for 5 min at room temperature to remove cellular debris. Supernatants (containing GPMVs) were then carefully collected, followed by ultracentrifugation at 30,000*g* for 30 min at 4°C using a Beckman Coulter, Optima XE-90 ultracentrifuge. After removing supernatant, pelleted GPMVs and ∼500 μl of residual buffer were snap-frozen in liquid nitrogen and transferred to a −80°C freezer for storage.

We extracted lipids using the modified Folch method, exactly as described above for the In vitro #1 lipidomic experiment. Before extraction, we mixed lipid extracts with internal standards mixture containing 13 deuterated standards (EquiSPLASH mix, Avanti Polar Lipids, 330731) (0.5 μl per sample), together with 0.1 μl of Cholesterol (d7) (Avanti Polar Lipids, 700041) for quantification and normalization (table S1). This experiment followed identical procedures as those outlined for the In vitro #1 lipidomic experiment.

### Western blot analysis

We verified the enrichment of GPMVs in plasma membrane (and depletion in internal organelle) by Western blot analysis. For whole cell lysate, 13 × 10^6^ qNSCs (passage 5 to passage 6) were plated in a 15-cm plate (∼90,000 cells/cm^2^) and quiescence was induced for 7 days (see above in the “Primary cultures of qNSCs and aNSCs” section). Cells were then lysed directly in the culture plates using 3 ml of ice-cold 1× radioimmunoprecipitation assay (RIPA) buffer diluted from 5× RIPA buffer (Thermo Fisher Scientific, J62524AD) with ultrapure water (Invitrogen, 10977-015) and supplemented with 1× protease inhibitor cocktail (Roche, 04693159001). Cell lysates were then scraped and transferred to Eppendorf tubes. GPMVs were generated from 13 × 10^6^ qNSCs (passage 5 to passage 6) as described above in the “GPMV generation and design of the “GPMV” lipidomic experiment” section. GPMV protein lysates were obtained by lysing GPMV pellet with 50 μl of 1× RIPA buffer with 1× protease inhibitor cocktail. Following the addition of Laemmli sample buffer (Alfa, Aesar, J61337), all lysates were resolved on NuPAGE 4 to 12% gradient bis-tris gel (Invitrogen, NP0321BOX), transferred onto nitrocellulose membranes (Bio-Rad, 162-0115), and immunoblotted. For each sample, equal amounts of total protein was loaded based on protein quantification using Ponceau S staining (see below). Primary antibodies used were as follows: 1:25,000 Beta-actin (Abcam, Ab6276, RRID:AB_2223210), 1:5000 Calnexin (Abcam, Ab22595, RRID:AB_2069006), and 1:5000 Cox4 (Abcam, Ab16056, RRID:AB_443304). Membranes were then incubated with secondary antibody IRDye 680RD donkey anti-mouse IgG (LI-COR, 926-68072, RRID:AB_10953628), IRDye 800CW goat anti-rabbit IgG (LI-COR, 926-32211, RRID:AB_621843), or IRDye 680RD goat anti-rat IgG (LI-COR, 926-68076, RRID:AB_10956590). All secondary antibodies were used at 1:5000 dilution. Blots were imaged on Odyssey XF Imager (LI-COR).

### Ponceau S staining and total protein quantification

Following protein gel transfer onto nitrocellulose membranes, the membranes were incubated in Ponceau S solution (Sigma-Aldrich, P7170-1L) for 5 min at room temperature and then washed three times (1 min each time) with Milli-Q water. The membrane was immediately scanned on an Epson perfection V300 photo scanner (model no. J232B) for densitometry quantification. Using ImageJ (version 2.1.0/1.53c), the images were split into red, blue, and green channels. Equal sized rectangles were drawn around each lane, and the mean integrated density from each lane of each channel was measured. The sum of the mean integrated density from all three channels was compared to verify that the same amount of protein was loaded into each lane.

### Overlap, effect size, and abundance between lipids with significant age-associated changes in whole cell extracts and GPMVs

We identified the overlap between lipids in our GPMV lipidomic dataset (enriched for plasma membrane) and the In vitro #1 lipidomic dataset (whole cell extract) using the same method as described above in the “Overlapping lipids between in vitro lipidomic datasets” section. We also compared the effect size of lipids and side chain features that change with age between GPMV and In vitro #1 lipidomic datasets as described above for the comparison across in vitro lipidomic datasets.

For abundance, lipids were ranked based on the normalized concentration. Significant lipids in both GPMV and whole cell lipidomic data were highlighted. Many of these lipids were of high abundance, with concentration in the top 50 percentile of all lipids.

### Laurdan staining and quantification of plasma membrane order in vitro

Membrane order was assessed by staining with the polarity-sensitive membrane probe Laurdan (6-dodecanoyl-2-dimethylaminonaphthalene) (Thermo Fisher Scientific, D250) (*104*–*107*). Ratiometric quantification of Laurdan at the plasma membrane provides a measure of membrane order (*107*). The Laurdan probe is excited at 405 nm. The Laurdan dye fluoresces with a peak emission wavelength around 450 nm (visualized in green throughout the study) when present in the ordered phase of membrane lipids and ∼500 nm (visualized in red throughout the study) when present in the disordered phase of membrane lipids (*107*) (Fig. 5A).

Specifically, 90,000 qNSCs from primary culture of young and old mice were plated in each well of an eight-well chamber coverslip (90,000 cells/cm^2^) (Ibidi, 80826). After 7 days of induced quiescence in quiescence media, cells were stained with Laurdan at a final concentration of 30 μg/ml in culture media for 30 min in a 37°C tissue culture incubator. Cells were then washed once with media. DNA stain DRAQ5 (Thermo Fisher Scientific, 62251) was added at a final concentration of 5 μM in media, immediately before imaging. Live-cell imaging was carried out in a 37°C imaging chamber with 5% CO_2_ using a Zeiss LSM980 microscope. When capturing the intensity of membrane lipids in the ordered phase, BP (bandpass) 420 to 480 was used. Signal obtained is referred to as *I*_420–480_ below. When capturing the intensity of membrane lipid in the disordered phase, BP 465 to 505 and LP (longpass) 525 was used. Signal obtained is referred to as *I*_465–505_ below. Next, five images from each condition were taken using 60× objective with Airyscan 2 in Multiplex mode.

We generated an automated pipeline to perform ratiometric quantification of the Laurdan data in an unbiased manner. Image analysis was performed using CellProfiler 4.1.3. Briefly, nuclei were identified with the “IdentifyPrimaryObjects” module using global Otsu thresholding. Cellular boundaries were then identified with labeled nuclei as seeds, using the propagation method within the “IdentifySecondaryObjects” module. The outermost 10 pixels of each cellular boundary were saved as plasma membrane–specific masks. Plasma membrane masks were then applied to each individual cells in Fiji (v2.1.0/1.53c).

Normalized GP ratio of plasma membrane was quantified as previously described (*105*).

Normalized GP ratio = (*I*_420–480_ – *G* * *I*_465–505_)/(*I*_420–480_ + *G* * *I*_465–505_), where

$$G=\left({\text{GP}}_{\text{ref}}+{\text{GP}}_{\text{ref}}*{\text{GP}}_{\text{mes}}-{\text{GP}}_{\text{mes}}-1\right)/\left({\text{GP}}_{\text{mes}}+{\text{GP}}_{\text{ref}}*{\text{GP}}_{\text{mes}}-{\text{GP}}_{\text{ref}}-1\right)$$

GP_ref_ = 0.207 (*165*).

GP_mes_ was obtained from a reference image with media containing the identical concentration of Laurdan (30 μg/ml) without any cells and represents the background signal intensity in both imaging channels (*I*_420–480_ and *I*_465–505_). Reference images were taken in the same imaging session with identical parameters in all samples of the same experiment.

### FLIM in vitro

Membrane order was also assessed by an orthogonal approach, FLIM, by staining of primary NSC cultures with the polarity-sensitive membrane probe di-4-ANEPPDHQ (Invitrogen, D36802) (*108*, *109*). Specifically, 90,000 qNSCs (90,000 cells/cm^2^) from primary culture of young and old mice were plated in each well of eight-well chamber coverslip (Ibidi, 80826). After 7 days of induced quiescence, cells were stained with di-4-ANEPPDHQ at a final concentration of 5 μM in culture media for 1 hour in a 37°C tissue culture incubator. FLIM imaging was carried out using a Zeiss LSM780 microscope using 60× objective. Excitation was set at 475 nm by a frequency-doubled, Mai Tai DeepSee laser oscillator (Spectra-Physics). Fluorescence lifetime is the average time a fluorophore remains in the excited state before emitting a photon and returning to the ground state. It was measured using a time-correlated single photon counting (TCSPC) card (Becker & Hickl GmbH) and fitted using an incomplete multiexponential model on the SPCM software (Becker & Hickl GmbH). A longer lifetime is associated with a more ordered (rigid) membrane, whereas a shorter lifetime is associated with a more disordered (fluid) membrane (*166*–*168*).

### Stratification of plasma membrane order quantification in vitro by cell density

As plasma membrane order assessment could be affected by cell density, we examined this parameter in more depth. We ensured that equal numbers of qNSCs (90,000 cells per well of eight-well chamber coverslips, 90,000 cells/cm^2^) for each condition were seeded at the beginning of all Laurdan and FLIM experiments in vitro. For plasma membrane order quantification by Laurdan staining and by FLIM: 60× images were taken from different areas on the plate with varying cell density. Confocal images were then stratified based on the number of cells per image into low-density (5 to 10 cells) and high-density (15 to 20 cells) images. Plasma membrane order quantification on each single cell from low- and high-density images was assessed between young and old qNSCs.

### Stratification of plasma membrane order quantification in vitro by cell size

As plasma membrane order assessment could be affected by cell size, we also examined this parameter in more depth. Individual cell size was obtained using the “Area” output from CellProfiler on previously identified cell boundary (see the “Laurdan staining and quantification of plasma membrane order in vitro” above). The median cell size from all quantified cells was identified. Each individual cell was then stratified into “small” or “large” based on whether the measured cell size is smaller or bigger than the median of all cells. Plasma membrane order quantification of individual cells from each stratified group was assessed between young and old qNSCs.

### In vivo Laurdan staining and quantification

For assessment of plasma membrane order in the SVZ neurogenic niche in vivo, we also used Laurdan—a standard approach to measure plasma membrane order (*107*). Although other imaging technologies (e.g., electron microscopy) might provide higher spatial resolution, the ability to simultaneously detect cell types with antibody staining and to perform Laurdan quantification by confocal microscopy is critical in assessing NSC-specific membrane order in the SVZ neurogenic niche. For Laurdan staining and quantification, *n* = 5 young (3 to 5 months old) and *n* = 5 old (20 to 22 months old) C57BL/6 male mice were subjected to transcardiac perfusion with 4% paraformaldehyde (Electron Microscopy Sciences, 15714) in PBS (Corning, 21-040-CV). Brains were postfixed overnight in 4% paraformaldehyde (Electron Microscopy Sciences, 15714) and then dehydrated in 30% sucrose (Sigma-Aldrich, S3929) for 72 hours. Brains were embedded in Tissue-Tek OCT compound (Electron Microscopy Sciences, 62550), sectioned into 16-μm coronal slices using a cryostat (Leica CM3050S), and then mounted on glass slides (Thermo Fisher Scientific, 12-550-15). Coronal sections between Bregma 0.5 to 1 mm were generated for each mouse. We used Laurdan staining in combination with cell-type markers GFAP and EGFR to quantify plasma membrane order in qNSCs/astrocytes in vivo and we used localization in the SVZ and morphology to focus on qNSCs (compared to niche astrocytes). Antibodies against GFAP (Abcam, ab53554, RRID:AB_880202) were used at 1:1000 dilution, and antibodies against epidermal growth factor receptor (EGFR) (Millipore, 06-847, RRID:AB_2096607) were used at 1:200 dilution at 4°C overnight. Donkey anti-goat antibodies conjugated with Alexa Fluor 647 (Thermo Fisher Scientific, A-21447, RRID:AB_141844) and donkey anti-rabbit antibodies with Alexa Fluor 568 (Thermo Fisher Scientific, A-10042, RRID:AB_2534017) were used at 1:250 dilution at room temperature for 1.5 hours. Sections were subsequently stained with Laurdan at a final concentration of 30 μg/ml for 30 min at room temperature. Sections were then washed with PBS before being mounted with 50 μl of Vectashield anti-fade mounting media (Vector Laboratories, H-1000-10) before being imaged. We performed single-antibody staining to verify that other stains did not interfere with Laurdan spectra. We could not evaluate the potential interference from other stains on Laurdan by performing in vivo Laurdan staining alone without antibody staining because cell-type markers (GFAP and EGFR) were required for qNSC (and astrocyte) identification in vivo. Nevertheless, when we changed GFAP and EGFR antibody staining to different fluorescence channels, the quantification results on Laurdan remained the same.

Confocal imaging was carried out using a Zeiss LSM980 microscope using 60× objective with Airyscan 2 in Multiplex mode. When capturing the intensity of membrane lipids in the ordered phase, BP 420 to 480 and BP 495 to 550 was used. When capturing the intensity of membrane lipids in the disordered phase, BP 465 to 505 and LP 525 was used. For quantification, five to seven z-stacks were taken for each individual animal with 10 to 15 images in each stack using 0.3-μm intervals between images. We first used maximum intensity projection for preliminary identification of qNSCs based on cell-type markers (GFAP^+^ EGFR^−^) as well as spatial localization in the SVZ niche and morphology. qNSCs were identified manually using the following criteria: (i) positive GFAP staining surrounding the entire cell body in at least two consecutive single-plane images in the stack, (ii) positive GFAP staining partially detected throughout the rest of the z-stack for the same cell, (iii) negative EGFR staining throughout all images for the same cell, (iv) localization immediately adjacent to ependymal cells that line the ventricular wall, and (v) presence of long basal process that is characteristic to qNSCs (Fig. 5D and fig. S10, D and E). Once qNSCs were identified, plasma membrane–specific masks were hand traced based on the GFAP staining signal immediately surrounding the cell body and apical process using Fiji (v2.1.0/1.53c). Ratiometric quantification of Laurdan staining was performed using our automated pipeline (see the “Laurdan staining and quantification of plasma membrane order in vitro” section). A normalized GP ratio of plasma membrane was then generated as described in the “Laurdan staining and quantification of plasma membrane order in vitro” section.

### Choice of lipid-modifying enzymes for CRISPR-Cas9–based functional perturbations

For CRISPR-Cas9–based functional perturbations, we focused on five lipid metabolic enzymes (MBOAT2, AGPAT3, PLA2G4E, ELOVL5, and FADS2) because they are involved in the two age-dependent lipid changes we observed in our lipidomic data in qNSCs: remodeling of specific phospholipids (MBOAT2, AGPAT3, and PLA2G4E) and accumulation of PUFA-containing lipids (ELOVL5 and FADS2). All five enzymes satisfy two of the three following criteria: (i) highly expressed in qNSCs based on our single-cell RNA-seq data (*85*), (ii) significant change in expression with age (fig. S11), or (iii) substrate specificity in support of PUFA side chain remodeling in membrane lipid classes.

Specifically:

We chose MBOAT2 because it is a phospholipid acyl transferase that remodels phospholipids, several of which significantly changed with age. In addition, among the 3 *Mboat* genes, *Mboat2* was highly expressed in qNSCs/astrocytes and showed a significant age-related decrease in gene expression with age in qNSCs/astrocytes (fig. S11), and we have validated this decrease with age by in situ hybridization (Fig. 6E).

We chose AGPAT3 because it plays a crucial role in the synthesis of phospholipids, several of which significantly changed with age. In addition, *Agpat3* is the only isoform in the family that satisfies the combined criteria of (i) relatively high expression level in qNSCs/astrocytes (though it did not change with age) (fig. S11) and (ii) substrate specificity for PUFA-containing lipids.

We chose PLA2G4E because it has phospholipase activity, corresponding to several phospholipids that significantly changed with age in qNSCs. In addition, *Pla2g4e*, while not highly expressed in qNSCs/astrocytes (fig. S11), has an *N*-acyltransferase activity that produces *N*-acyl-ethanolamine phospholipids, and those lipids have been shown to accumulate in brain aging (*169*).

We chose ELOVL5 because, together with FADS2, it works sequentially in PUFA biosynthesis pathway through stepwise elongation (ELOVL5) and desaturation (FADS2). Several PUFAs were remodeled with age in qNSCs/astrocytes (Figs. 1E, 2B, and 4B). Moreover, ELOVL5 (together with FADS2) is involved in generating the most abundant PUFA species in the brain (e.g., 18:3, 20:3, 20:4, and 20:5), based on studies on different regions of the brain (*170*). In addition, *Elovl5* had the highest expression level in qNSCs/astrocytes compared to other family members (*Elovl1*, *Elovl2*, *Elovl3*, *Elovl4*, *Elovl6*, and *Elovl7*) and showed decreased expression with age (fig. S11).

We chose FADS2 because, together with ELOVL5, it works sequentially in PUFA biosynthesis pathway through stepwise elongation (ELOVL5) and desaturation (FADS2). Several PUFAs were remodeled with age in qNSCs (Figs. 1E, 2B, and 4B). Moreover, FADS2 (together with ELOVL5) is involved in generating the most abundant PUFA species in the brain (e.g., 18:3, 20:3, 20:4, and 20:5), based on studies on different regions of the brain (*170*). FADS2 is also required for the synthesis of PUFA 22:6, another major PUFA species in the brain. In addition, *Fads2* exhibited a slight but significant decrease in gene expression in qNSCs/astrocytes with age (fig. S11).

### Analysis of gene expression from single-cell RNA-seq

Normalized gene counts from our previously published single-cell RNA-seq data (*85*) were used to plot the expression level of the five genes of interest and genes encoding the same family of enzymes:

1) *Elovl1*, *Elovl2*, *Elovl3*, *Elovl4*, *Elovl5*, *Elovl6*, and *Elovl7*

2) *Mboat1*, *Mboat2*, *Lpcat3* (*Mboat5*), and *Mboat7*

3) *Fads1* and *Fads2*

4) *Agpat1*, *Agpat2*, *Agpat3*, *Agpat4*, and *Agpat5*

5) *Pla2g4a*, *Pla2g4b*, *Pla2g4c*, *Pla2g4d*, *Pla2g4e*, and *Pla2g4f*

### sgRNA cloning for in vitro gene knockouts of *Mboat2*, *Elovl5*, *Agpat3*, *Fads2*, and *Pla2g4e*

LentiCRISPR v2 was a gift from F. Zhang (Addgene plasmid no. 52961; http://n2t.net/addgene:52961; RRID:Addgene_52961). psPAX2 was a gift from D. Trono (Addgene plasmid no. 12260; http://n2t.net/addgene:12260; RRID:Addgene_12260). pCMV-VSV-G was a gift from R. Weinberg (Addgene plasmid no. 8454; http://n2t.net/addgene:8454; RRID:Addgene_8454). sgRNAs targeting *Mboat2*, *Elovl5*, *Agpat3*, *Fads2*, and *Pla2g4e* together with non-targeting and safe-targeting sgRNAs were selected from previously published genome-wide sgRNA library (*171*). For a list of sgRNAs used for in vitro gene knockouts, see table S8. sgRNA oligos were ordered from Integrated DNA Technologies and subcloned into lentiCRISPR v2 plasmid using the BsmB I restriction site as previously described (*172*). Sanger sequencing was performed to verify the constructs. One sequencing primer verifying the insertion of sgRNA and the 5′ end of the Cas9 sequence was used in conjunction with a second sequencing primer verifying the 3′ end of the Cas9 sequence.

### Lentiviral production and transduction for in vitro CRISPR-Cas9–mediated knockouts of *Mboat2*, *Elovl5*, *Agpat3*, *Fads2*, and *Pla2g4e*

For lentiviral production, human embryonic kidney (HEK) 293T cells (ATCC, CRL-3216) were seeded at a density of 13 × 10^6^ cells per 15-cm plate in Dulbecco’s modified Eagle’s medium (DMEM) (Thermo Fisher Scientific, 11965092) with 10% fetal bovine serum (Gibco, 10099-141, lot 1751896) and penicillin-streptomycin-glutamine 1× (Gibco, 10378-016). Two days after plating, HEK293T media was changed and the cells were transfected using polyethylenimine (1 mg/ml) (Polysciences, 23966-2).

LentiCRISPR v2 (45 μg) was transfected together with the lentiviral packaging plasmids psPAX2 (28.35 μg) and pCMV-VSV-G (8.325 μg) per 15-cm plate. One day (20 to 24 hours) after transfection, the media was changed to NeuroBasal-A with penicillin-streptomycin-glutamine. After another 20 to 24 hours, lentivirus-containing supernatant was collected and stored at 4°C and fresh media was added to the HEK293T cells for another collection after 24 hours. Both supernatants were then combined, filtered through a 0.45-μm polyvinylidene fluoride (PVDF) filter (Millipore Sigma, SE1M003M00), and frozen at −80°C in 3-ml aliquots.

For lentiviral transduction, 900,000 NSCs (passage 3 to passage 5) were plated in each well of a six-well plate (∼94,000 cells/cm^2^) maintained in Quiescence NSC media for 4 days (with media change every other day) before transduction. Next, 750 μl of lentiCRISPR v2 supernatant was thawed on ice and mixed with 250 μl of NeuroBasal-A + penicillin-streptomycin-glutamine with 8% of B27 minus vitamin A, bFGF (80 ng/ml), and BMP4 (200 ng/ml) and added to each well of a six-well plate. qNSC cultures were incubated with viral media for 24 hours. The viral media was removed and a second lentiviral transduction was repeated for another 24 hours. Next, cells were washed once with NeuroBasal-A media and cells were kept in Quiescence NSC media for 7 days to allow CRISPR editing. Fresh Quiescence NSC media was replaced every 2 days. This 7-day period ensures a time window for both target gene editing and effect on the phenotype of interest (lipidomics, qNSC activation). To assess the efficiency of viral transduction and reproducibility, a positive control lentiviral construct pLJM1-EGFP (Addgene, 19319) was used in parallel. The fraction of transduced cells was quantified as EGFP^+^ cells using an LSR-II flow cytometer (BD Biosciences) 48 hours after transduction. The lentiviral construct we used did not include any tag or reporter that could be readily used as a readout for transduction. We therefore used the knockout score generated by Inference of CRISPR Edits (ICE) on harvested genomic DNA as a proxy to assess transduction efficiency (see the “CRISPR editing efficiency of individual knockouts in vitro of *Mboat2*, *Elovl5*, *Agpat3*, *Fads2*, and Pla2g4e by ICE analysis” section below). We verified that the knockout of each enzyme gene was efficient in both young and old qNSCs, with a frameshift editing of 44 to 69% of the alleles for each gene in both young and old qNSCs (except *Agpat3*) (fig. S12A).

### CRISPR editing efficiency of individual knockouts in vitro of *Mboat2*, *Elovl5*, *Agpat3*, *Fads2*, and *Pla2g4e* by ICE analysis

CRISPR editing efficiency of individual knockouts was assessed by analyzing genomic DNA. Briefly, genomic DNA was collected from qNSCs 7 days following lentiviral transduction. Genomic DNA was extracted after lysing qNSCs with 200 μl of DirectPCR lysis reagent (Viagen biotech, 102-T) for 15 min at room temperature. Lysates were then incubated with Protease K (0.2 mg/ml; Invitrogen, 25530049) for 25 min at 65°C, followed by protease deactivation for 15 min at 95°C. We then amplified genomic DNA with primer pairs roughly 150 to 250 bp upstream and 450 to 500 bp downstream of sgRNA editing site, with 30 s of annealing step at 55°C and 1 min of extending step at 72°C for 40 cycles total. See table S10 for a complete list of primers.

We used the knockout score generated by Inference of CRISPR Edits (ICE) (Synthego Performance Analysis, ICE Analysis. 2019. v2.0. Synthego) to assess editing efficiency, according to the vendor’s instruction. Knockout score was calculated by ICE based on the fraction of alleles from the genomic DNA of edited samples with either a frameshift or 21+ bp indel, using an unedited sample as control. All knockouts had an editing efficiency between 55 and 70%, except the *Agpat3* knockout, which had an efficiency at 27%.

### Lipidomic dataset of CRISPR-Cas9–mediated knockouts in vitro of *Mboat2*, *Elovl5*, *Agpat3*, *Fads2*, and *Pla2g4e* in young and old qNSCs

We generated a lipidomic LC-MS/MS dataset on *n* = 4 primary qNSC cultures (each with 1 × 10^6^ qNSCs) from young and old mice, each with either control lentivirus or lentivirus expressing sgRNAs to the gene of interests (*Mboat2*, *Agpat3*, *Pla2g4e*, *Elovl5*, and *Fads2*). This dataset was the same as In vitro #2 already described above (tables S1 and S9).

### Quantification of substrate and product level in *Mboat2*, *Elovl5*, *Agpat3*, *Fads2*, and *Pla2g4e* knockouts in vitro

To evaluate the effectiveness of each gene knockout at the metabolite level, we quantified the lipid concentration of known substrates and products of each enzyme in cells with knockout for each gene with high efficiency knockouts (*Elovl5*, *Mboat2*, *Fads2*, *Pla2g4e* knockouts). Specifically, we quantified the concentration of free fatty acids 18:2 and 18:3, and the concentration of free fatty acids 20:2 and 20:3, as substrates and products for ELOVL5, respectively. We quantified the concentration of LPE(16:0), and the summary concentration of all PE containing 16:0, as substrate and product for MBOAT2, respectively. We quantified the concentration of free fatty acids 18:2 and 20:2, and the concentration of free fatty acids 18:3 and 20:3, as substrates and products for FADS2, respectively. We quantified the summary concentration of all PC containing 16:0, and the concentration of LPC(16:0), as substrate and product for PLA2G4E, respectively.

### Lipidomic similarity and differences between *Mboat2*, *Elovl5*, *Agpat3*, *Fads2*, and *Pla2g4e* knockouts in vitro

To evaluate the similarity and differences in lipidomic profile as a result of knockouts, we first quantified the effect size on all lipids between each knockout compared to control samples (table S9). We separated lipids into “Lipids enriched in gene knockout (effect size > 0 when compared to control)” or “Lipids depleted in gene knockout (effect size < 0 when compared to control)” groups. We then took lipids from the top 20 percentile of each group and compared the overlap between different knockout conditions using an upset plot with the R package “UpSetR” v1.4.0.

### NSC activation assay in vitro

To determine the effect of different interventions (e.g., genetic perturbations and plasma membrane lipid supplementation) on qNSC function, we examined the ability of qNSCs to activate out of quiescence in vitro. We assessed qNSC activation efficiency by FACS to quantify the proportion of cells expressing proliferation marker Ki67, upon reintroduction of growth factors (*111*). qNSCs were washed twice with PBS (Corning, 21-040-CV) at 37°C before switching to Proliferative NSC media for 4 days. Media was replaced every 2 days. Cells were dissociated with Accutase (STEMCELL Technologies, 07920) for 5 min, collected into conical tubes, and centrifuged at 300*g* for 5 min. Next, 1 × 10^6^ cells were resuspended in 500 μl of FACS buffer [PBS with 2% fetal bovine serum (Gibco, 10099-141, lot 1751896)]. Following this step, 500 μl of 3.2% formaldehyde, diluted from 16% formaldehyde (Thermo Fisher Scientific, 28906), was added to the suspension dropwise under agitation to achieve a final concentration of 1.6% formaldehyde. Cells were fixed in 1.6% formaldehyde for 10 min at room temperature before spinning down at 700*g* for 5 min. Cells were washed once with FACS buffer before permeabilizing with 1 ml of ice-cold 100% methanol (Thermo Fisher Scientific, A4121). Following a period of 20 min of incubation on ice, cells were then washed again and resuspended in 200 μl of FACS buffer for staining. Ki67-APC (eBioscience, 17-5698-82, RRID:AB_2688057) antibody was used at 1:300 dilution. Samples were incubated in the dark for 30 min at room temperature with gentle agitation. Cells were washed again before adding DAPI (Thermo Fisher Scientific, 62248) at a final concentration of 0.2 μg/ml in FACS buffer. Each sample was filtered with FACS-strainer cap tubes (Falcon, 352235), just before FACS analysis. Cells were analyzed on a BD LSR II flow cytometer, and Flowjo (v10) software was used for data analysis.

For samples with *Mboat2* overexpression, an anti-FLAG antibody was also used to identify cells with overexpression construct. 3×-FLAG antibody (Cell Signaling Technology, 87537S, lot 1, RRID not available) was used at 1:200 dilution. Cells were washed again, followed by incubation with a secondary antibody of donkey anti-rabbit 568 (Invitrogen, A-10042, lot 2306809, RRID:AB_2534017) used at 1:250 dilution.

### Generation of an *Mboat2* overexpression construct

To overexpress *Mboat2* in qNSCs, we generated an overexpression construct containing the full-length coding sequence of mouse *Mboat2* transcript variant 1(NM_026037.3) tagged with a 3× FLAG tag (DYKDDDDK) and 3× GGGGS linker at the N terminus of the MBOAT2. The overexpression construct was driven by a human *EF1*α promoter in a lentiviral vector (pLV). The lentiviral vector used to overexpress *Mboat2* in our study, pLV[Exp]-EF1A>3×FLAG/3×GGGGS/mMboat2[NM_026037.3], was synthesized by VectorBuilder. The vector ID is VB230119-1418wdg, which can be used to retrieve detailed information about the vector on vectorbuilder.com. See table S11 for the complete sequence of the construct. A control construct with the same lentiviral backbone expressing enhanced green fluorescent protein (EGFP) under the same *EF1*α promoter was used (VectorBuilder #VB010000-9483amc).

### Lentiviral production and transduction for *Mboat2* overexpression in vitro

To generate lentiviruses overexpressing *Mboat2*, we transfected 45 μg of *Mboat2* overexpression (*Mboat2* OE) construct or the EGFP control construct, together with the lentiviral packaging plasmids psPAX2 (Addgene plasmid no. 12260) (28.35 μg), and pCMV-VSV-G (Addgene plasmid no. 8454) (8.325 μg) in HEK293T cells in a 15-cm plate, using the same method as described in the “Lentiviral production and transduction for in vitro CRISPR-Cas9–mediated knockouts of *Mboat2*, *Elovl5*, *Agpat3*, *Fads2*, and *Pla2g4e*” section*.*

For lentiviral transduction, qNSCs (900,000 seeded in each well of six-well plates, ∼94,000 cells/cm^2^) were maintained in Quiescence NSC media for 4 days (with media change every 2 days) before transduction. For transduction, 750 μl of *Mboat2* OE or EGFP control supernatant was thawed on ice and mixed with 250 μl of NeuroBasal-A + penicillin-streptomycin-glutamine with 8% of B27 minus vitamin A, bFGF (80 ng/ml), and BMP4 (200 ng/ml) (“Viral media”). qNSC cultures were incubated with viral media for 24 hours. Cells were then washed once with NeuroBasal-A media and then kept in Quiescence NSC media for 3 additional days before being harvested for lipidomic analysis or used for NSC activation assay (see the “NSC activation assay in vitro” section). We used the GFP reporter and the FLAG-tag to assess transduction of the control (GFP) and *Mboat2* overexpression (FLAG) lentivirus, respectively. We verified that the transduction efficiency by the *Mboat2* overexpression lentivirus was similar in both young and old qNSCs (53 to 55%) (fig. S12H).We validated that this experimental timeline (4 days in quiescence, one-time viral transduction, and 3-day incubation in quiescent media for a total of 8 days in quiescence) led to a fivefold increase in *Mboat2* mRNA level in cells transduced with *Mboat2* overexpression virus (fig. S12G).

### Design of “*Mboat2* overexpression” lipidomic experiments

To assess the effects of *Mboat2* overexpression on the lipidome of qNSCs, we generated a lipidomic LC-MS/MS dataset on *n* = 4 primary qNSC cultures (1 × 10^6^ qNSCs) from young and old mice, each with either control lentivirus or lentivirus overexpressing *Mboat2* as described above in the “Lentiviral production and transduction for *Mboat2* overexpression in vitro” section. This experiment and analysis were done as outlined for our In vitro #1 lipidomic experiment, with the only difference being the use of a new column with the same catalog number.

### Lentiviral production for in vivo *Mboat2* overexpression

For producing lentivirus used in in vivo *Mboat2* overexpression experiments, 45 μg of *Mboat2* overexpression (*Mboat2* OE) or EGFP control plasmid was transfected together with the lentiviral packaging plasmids psPAX2 (28.35 μg) and pCMV-VSV-G (8.325 μg), identical to *Mboat2* overexpression experiments in vitro. All transfections were done in HEK293T cells (ATCC, CRL-3216) plated in 15-cm plates. We plated four 15-cm plates of 293T cells (13 × 10^6^ cells per 15-cm plate) for a total of 200 ml of collected virus after 3 days of collecting at 4°C, followed by ultracentrifugation to concentrate the virus. For ultracentrifugation, we sterilized 30-ml ultraclear tubes (Beckman Coulter 344058) under UV (TC room biosafety cabinet) for 15 min. We placed these tubes on ice, allowing them to cool for 15 min. We then added 30 ml of virus and centrifuged at 16,500 rpm for 1 hour at 4°C. We carefully decanted the supernatant using serological pipettes, leaving 1 ml of medium in the bottom of the tube, adding 30 ml more virus-containing medium and centrifuging again. We repeated the decanting, refilling, and centrifugation of the same tube, concentrating a total of 180 ml of virus supernatant into a single tube. After the last ultracentrifugation, we removed most of the supernatant with a serological pipette, and the last 1 ml with a P1000 pipet tip from the side of the tilted tube, so as not to disturb the viral pellet. The viral pellet was usually visible in the center of all of the tubes. We resuspended in 60 μl of ice-cold PBS (1/3000th original volume) by pipetting up and down about 60 times, being careful not to produce air bubbles. The concentrated resuspended virus was then aliquoted into PCR strip tubes in 5-μl aliquots and placed onto dry ice. After 15 min, the concentrated virus preparation was transferred to a −80°C freezer for storage. For each experiment, the concentrated virus preparation was thawed on ice and injected into the brain within 30 min of thawing. We assessed virus infectivity of each batch by performing serial dilution (3, 1, and 0.5 μl) infections of 2 × 10^5^ HEK293T cells in 24-well culture plates for 16 hours of infection and then performing FACS analysis 48 hours later to detect the percentage of cells expressing FLAG tag and EGFP. For each experiment, we normalized virus infectivity (viral titer) across treatments by adjusting the concentrations of virus added in PBS.

### Surgeries for genetic perturbations in vivo

To perform genetic overexpression in vivo, stereotaxic surgeries were performed to inject lentivirus into the lateral ventricle of mice. Male C57BL/6JN mice were used for in vivo *Mboat2* overexpression experiment, and assigned to receive either EGFP control or *Mboat2* overexpression lentiviral particles in a randomized manner. Surgeries were performed on heating pads with isoflurane-induced anesthesia, with a Kopf (Model 940) stereotaxic frame, World Precision Instruments (UMP3T-1) UltraMicroPump3, a Hamilton 1710RN 100-μl syringe with a 30-gauge Small Hub RN needle with a point 2 beveled end. Injections were made at the following coordinates, relative to bregma: lateral 0.8 mm, anterior 0.3 mm, and ventral depth 2.5 mm from the skull surface. After drilling the skull and inserting the needle into position, we waited 5 min before injecting the virus. We injected 3 μl of equal titer virus at a rate of 10 nl/s. We waited 7 min after injection before removing the needle and suturing the skin. Animals were administered a single dose of buprenorphine SR (0.5 mg/kg) for postoperative pain management and monitored for 1 week after surgery until full recovery. For these experiments, *n* = 3 to 5 young or old animals were injected with either *Mboat2* overexpression virus or control virus. Brains were harvested 10 days after surgery.

### Brain section preparation for in vivo immunofluorescent staining and RNA in situ hybridization

Young and old anesthetized mice were first subjected to intracardiac perfusion with 4 ml of heparin (Sigma-Aldrich, H3149-50KU) and then 25 ml of 4% paraformaldehyde (PFA) (Electron Microscopy Science, 15714) in PBS. Brain tissue was dissected, and postfixed overnight in 4% PFA at 4°C. Brains were then washed three times for 10 min each in 1× PBS at 4°C, followed by dehydration in 30% sucrose (Sigma-Aldrich, S3929-1KG) in PBS for 2 to 3 days at 4°C or until brains sink to the bottom of the tube. Before embedding, all remaining liquid from brain tissue was removed with a Kimwipe. Samples were then embedded in OCT compound (Thermo Fisher Scientific no. 23-730-571), flash frozen in a dry ice and ethanol bath, and were stored at −80°C until sectioning. Brains were sectioned at 16 μm thickness using a LEICA cryostat (CM3050S). Sections were immobilized on Superfrost plus slides (Thermo Fisher Scientific, no. 12-550-15) in sets of five slides with four sections each alternating slide such that neighboring sections on each slide were 80 μm apart from each other in the sample tissue (e.g., slide 1 has the 1st, 6th, 11th, and 16th section of that set, slide 2 has the 2nd, 7th, 12th, and 17th section of that set, etc.) to capture a broader range of the SVZ within each slide. Slides were stored at −20°C until staining. Slides covering a range of roughly 320 to 640 μm deep into the SVZ for each animal were selected for staining and imaging.

### In vivo immunofluorescent staining on brain section

Slides were warmed to room temperature and washed for 5 min in 1× PBS and permeabilized in cold (−20°C) methanol with 0.1% Triton X-100 (Thermo Fisher Scientific, no. BP151-500) for 15 min. Slides were then washed three times in 1× PBS for 5 min. Sections were then segmented from each other using a hydrophobic marker (Thermo Fisher Scientific, NC1490738). Sections were blocked in a solution of 5% normal donkey serum (ImmunoReagents, SP-072-VX10) and 1% bovine serum albumin (Sigma-Aldrich, A7888-100G) in 1× PBS for 30 min at room temperature. Primary antibodies were diluted in blocking solution and used as follows: mCherry (Invitrogen, M11217, lot XJ359389, 1:500, RRID:AB_2536611), EGFR (Sigma-Aldrich, 06-847, lot 3173794, 1:200, RRID:AB_2096607), and GFAP (Abcam, ab53554, lot 1091347-1,1:5000, RRID:AB_880202). We added 75 μl of primary antibody/blocking solution per section and incubated overnight at 4°C in a humidified chamber. Slides were washed 3 times for 10 min each in PBS with 0.2% Tween 20 (Sigma-Aldrich, no. P1379-1L) followed by 1× PBS washes for 15 min repeated twice. Secondary antibodies were diluted in blocking solution and used as follows: anti-Goat Alexa Fluor 647 (Invitrogen, A-21447, lot 2273668, 1:500, RRID:AB_141844), anti-Rat Alexa Fluor 594 (Invitrogen, A-21209, lot 2400917, 1:500, RRID:AB_2535795), and anti-Rabbit Alexa Fluor 488 (Invitrogen, A-21206, lot 2668665, 1:500, RRID:AB_2535792). Next, 75 μl of secondary antibody/blocking solution was added to each section and then incubated at room temperature for 2 hours. Slides were washed three times in PBS with 0.2% Tween 20 for 10 min followed by three 1× PBS washes for 5 min. Slides were then mounted with ProLong Gold with DAPI (Invitrogen, no. P36934) and allowed to dry overnight at room temperature. Slides were sealed with clear nail polish and allowed to dry and then transferred to 4°C for storage before imaging.

### Hybridization chain reaction probe design

We performed hybridization chain reaction (HCR) to detect *Mboat2* transcript level in situ following the split-initiator probes design of HCR v3.0 (*173*). Specifically, 22 pairs (44 probes) of nonoverlapping probe sets were tiled across the entire consensus region of all isoforms of mouse *Mboat2* gene. Each probe was 22 nucleotides long and carried half of the HCR initiator sequence (gTCCCTgCCTCTATATCTTT and TTCCACTCAACTTTAACCCg). See table S10 for the complete list of HCR probe sets.

### HCR staining of brain sections

Slides were warmed to room temperature and washed for 5 min in PBS. Samples were permeabilized in cold (−20°C) methanol with 0.1% Triton X-100 for 15 min followed by a 5-min wash in PBS. Slides were then blot dried with a Kimwipe and 200 μl of hybridization buffer (Molecular Instruments, HCR Probe Hybridization Buffer, lot BPH02223) was added directly to each slide to cover samples and incubated for 30 min at 37°C in a humid chamber. After incubation, the hybridization buffer was removed. Pooled *Mboat2* targeting probes at 0.5 pmol/μl were diluted in hybridization buffer to a final concentration of 0.01 pmol/μl (1:50). Next, 100 μl probe/hybridization buffer solution was added to each slide and covered with a glass coverslip to prevent drying. The samples were then incubated at 37°C in a humid chamber overnight. After overnight incubation, the coverslip and probe/hybridization buffer solution was removed followed by five 15-min washes of 100% wash buffer (Molecular Instruments, HCR Probe Wash Buffer, lot BPW02323), 75% probe wash buffer/25% 5× SSCT [20× SSC (Invitrogen, AM9763) diluted to 5× with 0.1% Tween-20)], 50% probe wash buffer/50% 5× SSCT, 25% probe wash buffer/75% 5× SSCT, and 100% 5× SSCT in succession. After washes, slides were incubated in amplification buffer (Molecular Instruments, HCR Amplifier Buffer, lot BAM02223) for 30 min at room temperature in a humid chamber. Alexa Fluor 546–labeled h1 and h2 hairpins (Molecular Instruments, B3-h1-546, lot S073826, and B3-h2-546, lot S073926) at 3 μM were then snap cooled by heating to 95°C for 90 s in a thermal cycler and then allowed to cool to room temperature. Each hairpin was then diluted in amplification buffer to a final concentration of 60 nM (1:50). Hairpin/amplification buffer solution was then added to the slides, covered with a glass coverslip, and incubated overnight at 37°C in a humidified chamber. After overnight incubation, hairpin/amplification buffer solution was removed, and slides were washed twice for 30 min in 5× SSCT and washed again for 5 min in 5× SSCT at room temperature. For subsequent immunofluorescent staining, sections were blocked in a solution of 5% normal donkey serum and 1% bovine serum albumin in PBS. Primary antibodies were diluted in blocking solution: EGFR (Sigma-Aldrich, 06-847, lot 3173794, 1:200, RRID:AB_2096607) and GFAP (Abcam, ab53554, lot 1091347-1, 1:5000, RRID:AB_880202). Next, 75 μl of primary antibody/blocking solution was added to each section and incubated overnight at 4°C in a humid chamber. Slides were washed three times for 10 min in PBS with 0.2% Tween 20 followed by two washes in PBS for 15 min each. Secondary antibodies were diluted in blocking solution: anti-Rabbit Alexa Fluor 647 (Invitrogen, A-31573, lot 2420695, 1:500, RRID:AB_2536183) and anti-Goat Alexa Fluor 488 (Invitrogen, A-11055, lot 2301114, 1:500, RRID:AB_2534102). Next, 75 μl of secondary antibody/blocking solution was added to each section and incubated at room temperature for 2 hours in a humidified chamber. Slides were washed three times in PBS with 0.2% Tween 20 for 10 min each followed by three washes in PBS for 5 min. Slides were mounted with ProLong Gold with DAPI and allowed to dry overnight at room temperature. Slides were sealed with clear nail polish, allowed to dry, and transferred to 4°C for storage until imaging.

### Imaging of in vivo immunofluorescent staining or HCR of brain section

To image in vivo immunofluorescent staining of brain sections, we used confocal microscopy. For each section, we captured two single-plane tile images, one from the SVZ of each hemisphere, using a Zeiss LSM980 Airyscan 2 with a 40× glycerin-immersion lens. Using the polygon tool in Zeiss image capture software, we traced the outside of the SVZ to set the capture area. We used anchor points to set focal depth based on the DAPI signal to capture the middle of section while accounting for possible unevenness across tissue section.

### Image quantification of in vivo immunofluorescent staining and HCR on brain section

Raw images were analyzed using the open-source image analysis software QuPath. During analysis, sample IDs and image names were blinded using the “mask image names” feature. Using the polygon annotation tool, we annotated the SVZ to include the areas of highest DAPI signal along the ventricle as well as an additional cell layer. This annotation was used as the ROI for further analysis. Cell segmentation was performed using the “Cell Detection” function with DAPI as the detection channel using the default settings except for the following settings: background radius = 10 μm, minimum area = 15 μm^2^, maximum area = 100 μm^2^, and threshold = 35. Nuclei were expanded by 2 μm. For in vivo *Mboat2* overexpression samples, single measurement classifiers for GFAP and EGFR status were made by using the cell mean intensity for the respective channels and adjusting the threshold to detect positive cells as assessed by eye. Subsequently, single measurement classifiers for GFAP and EGFR were combined into a composite classifier to assign GFAP and EGFR status to the SVZ ROI. For HCR quantification, *Mboat2* transcript signals of each individual cell (presented as puncta in HCR images) were quantified using the “Subcellular Detection” function with the following settings: detection threshold = 1250, expected spot size = 0.35 μm^2^, min spot size = 0.15 μm^2^, and max spot size = 0.6 μm^2^ in all SVZ cells.

### *Mboat2* mRNA level in qNSC/astrocytes in vivo

We identified qNSCs/astrocytes (GFAP^+^ and EGFR^−^) on coronal sections of young and old mouse brains based on the single measurement classifier of GFAP and EGFR (described above). We then quantified the mean *Mboat2* transcript signals (described above) in qNSCs/astrocytes of each imaging tile to assess the *Mboat2* mRNA level in qNSC/astrocytes of young and old mice in vivo.

### Validation of *Mboat2* overexpression in vivo by HCR quantification

We use the *Mboat2* transcript signals (described above) from HCR to assess *Mboat2* overexpression in vivo*.* We use the number of identified puncta on HCR staining to determine a threshold value in identifying *Mboat2*-expressing cells. By visualizing the distribution of *Mboat2* transcript number on a histogram, we observe a distinct peak in cells with two or more detected *Mboat2* puncta. We then quantified the percentage of cells in each imaging tile at or above the threshold transcript level of *Mboat2* to assess the effectiveness of *Mboat2* overexpression in vivo.

### Image quantification on aNSC numbers following *Mboat2* overexpression in vivo

GFAP^+^ and EGFR^+^ (aNSCs) cells and GFAP^+^ and EGFR^−^ (qNSCs/astrocytes) cells were first identified using the GFAP and EGFR classifier described above (see the “Image quantification of in vivo immunofluorescent staining and HCR on brain section” section) in animals injected with *Mboat2* overexpression virus. Next, the percentage of aNSCs relative to the total number of NSCs/astrocytes (aNSCs + qNSCs/astrocytes) were quantified in control cells and *Mboat2*-overexpressing cells of each animal. Control and *Mboat2*-overexpressing cells were identified based on whether *Mboat2* transcript level was below or above the threshold transcript level of *Mboat2*, respectively, see the “Validation of *Mboat2* overexpression in vivo by HCR quantification” section. Note that one young animal died during the experiment, prompting the addition of two young control animals; two additional old control animals were also included to balance group sizes across age groups. All additional animals were injected with a control lentivirus expressing EGFP (see the “Lentiviral production for in vivo *Mboat2* overexpression” section) and are indicated by a distinct shape in the figure.

### Plasma membrane lipid supplementation in recipient qNSC cultures

For plasma membrane lipid supplementation of recipient qNSC cultures, GPMVs from young and old donor qNSCs (13 × 10^6^ qNSCs seeded in a 15-cm plate, 90,000 cells/cm^2^) were generated using the identical protocol described above. In addition to plasma membrane lipids, GPMVs also contain proteins. We therefore used the Folch method described above to isolate the lipid constituent of the GPMV before supplementing it into cell culture. Plasma membrane lipids extracted from GPMVs were resolubilized in 100 μl of 100% methanol and stored at −20°C before use. Young and old recipient qNSCs were incubated in quiescent media for 4 days before being supplemented with GPMV lipids or control solvent (100% methanol), both diluted 1:250 into Quiescence NSC media for 48 hours. Quiescence media was then replaced and cells were incubated in Quiescence NSC media for another 24 hours before being harvested for the different assays.

### Imaging of plasma membrane lipid uptake by qNSCs

To image plasma membrane lipid uptake by qNSCs, the lipophilic dye Vybrant DiI was introduced (at a dilution of 1:2000) to fluorescently label plasma membrane lipid extracts. Labeled lipid extracts were supplemented to qNSCs for 72 hours. Live-cell confocal imaging was done to capture cellular uptake of Vybrant DiI-labeled plasma membrane lipids, together with nuclear dye DAPI and lipophilipic dye Laurdan to visualize all membrane compartments of cells. Live-cell imaging was carried out in a 37°C imaging chamber with 5% CO_2_ using a Zeiss LSM980 microscope. Images were taken using 60× objective with Airyscan 2 in Multiplex mode.

### Design of lipid supplementation lipidomic experiment

To assess the effects of plasma membrane lipid supplementation on the lipidome, we generated a lipidomic LC-MS/MS dataset on *n* = 4 to 8 primary qNSC cultures from young and old mice, each with either control solvent or plasma membrane lipid extract as described in the “Plasma membrane lipid supplementation in recipient qNSC cultures” section. The lipidomic experiment and analysis were performed exactly as described above for our in vitro #1 lipidomic dataset, with the only difference being the use of a new column with the same catalog number.

### Effect size ranking of lipids that change with young plasma membrane supplementation

To determine whether lipids that showed a higher abundance following plasma membrane lipid supplementation were enriched at the plasma membrane, we first calculated lipid effect size between recipient qNSCs supplemented with young plasma membrane lipids and controls. We distinguished lipids that were “Higher in qNSCs with plasma membrane lipid supplementation (effect size > 0 when compared to control qNSCs)” and “Lower in qNSCs with plasma membrane lipid supplementation (effect size < 0 when compared to control qNSCs).” We then quantified the effect size between lipid from GPMV (of GPMV lipidomics) and from whole cell extract (from control samples of In vitro #1). We then identified overlapping lipids from both effect size analyses and ranked lipids based on the effect size comparison between whole cell extract and GPMV lipids in young and old samples. Lipids of the “Higher with plasma membrane lipid supplementation” and “Lower with plasma membrane lipid supplementation” groups were color coded on the plot.

### Comparison between the effect of young plasma membrane lipid supplementation on recipient qNSCs and age-related changes in GPMV lipids

To evaluate the effect of young plasma membrane lipid supplementation on recipient qNSCs, we calculated the effect size for individual lipids between recipient qNSCs (young or old) that were supplemented with young plasma membrane lipids and control recipient qNSCs. We then calculated effect size for individual lipids between GPMV samples from young and old qNSCs (see “GPMV generation and design of the GPMV lipidomic experiment”). We identified overlapping lipids from both analyses and plotted the result in a scatter plot for either young and old recipient qNSCs. On the basis of quadrants of the scatter plot, lipids were separated into four groups—Higher in recipient qNSCs with young plasma lipid supplementation and higher in young GPMVs, Higher in recipient qNSCs with young plasma lipid supplementation and higher in old GPMVs, Lower in recipient qNSCs with young plasma lipid supplementation and higher in young GPMVs, and Lower in recipient qNSCs with young plasma lipid supplementation and higher in old GPMVs. We then quantified the distribution of lipids in each of these categories as a percentage of the total number of lipids and presented them in a pie chart.

### Correlation between plasma membrane order and qNSC activation following plasma membrane lipid supplementation

To examine whether qNSC activation is correlated with plasma membrane order, we performed qNSC activation assay by FACS (see the “NSC activation assay in vitro” section) and plasma membrane order quantification (see “Laurdan staining and quantification of plasma membrane order in vitro” section) on the same set of samples following plasma membrane lipid supplementation. Percentage of cells with positive Ki67 staining and the normalized GP ratio were plotted for each sample. Correlation coefficient and *P* value were obtained from Pearson correlation analysis in R version 4.4.2.

### Statistical analyses

While we did not perform power analyses, we did use previous experiments to inform the experimental design with respect to sample size. To calculate statistical significance for experiments, all tests were two-sided Wilcoxon rank-sum tests unless otherwise indicated. We performed multiple hypothesis correction using FDR (Benjamini-Hochberg correction for multiple comparisons). For effect size, our statistical analysis is described in detail above and also includes multiple hypothesis correction using FDR (Benjamini-Hochberg correction for multiple comparisons). To test the effect of knockout in paired samples, we performed normality test to establish that data are normally distributed before using paired Welch’s *t* test before control and knockout samples. To test age difference within knockout condition, unpaired Welch’s *t* test was used. All tests were performed by R version 4.0.2. All results from individual experiments and all statistical analyses are included in table S14.
