# Supplementary material for: Lipidomic profiling reveals age-dependent changes in plasma membrane lipids that affect neural stem cell aging
Source: Sci Adv. 2026 Jul 29;12(31):eaeh9771. doi: 10.1126/sciadv.aeh9771 (PMC13418745; doi:10.1126/sciadv.aeh9771)
Supplement: Supplementary file 1 — Figs. S1 to S14 Table S1 Legends for tables S2 to S14 [file sciadv.aeh9771_sm.pdf]

Science Advances
AAAS

Supplementary Materials for

Lipidomic profiling reveals age-dependent changes in plasma membrane lipids that affect neural stem cell aging

Xiaoai Zhao *et al.*

Corresponding author: Xiaoai Zhao, xiaoai.zhao@yale.edu; Anne Brunet, abrunet1@stanford.edu

Sci. Adv. **12**, eaeh9771 (2026)
DOI: 10.1126/sciadv.aeh9771

The PDF file includes:

Figs. S1 to S14
Table S1
Legends for tables S2 to S14

Other Supplementary Material for this manuscript includes the following:

Tables S2 to S14

Figure S1

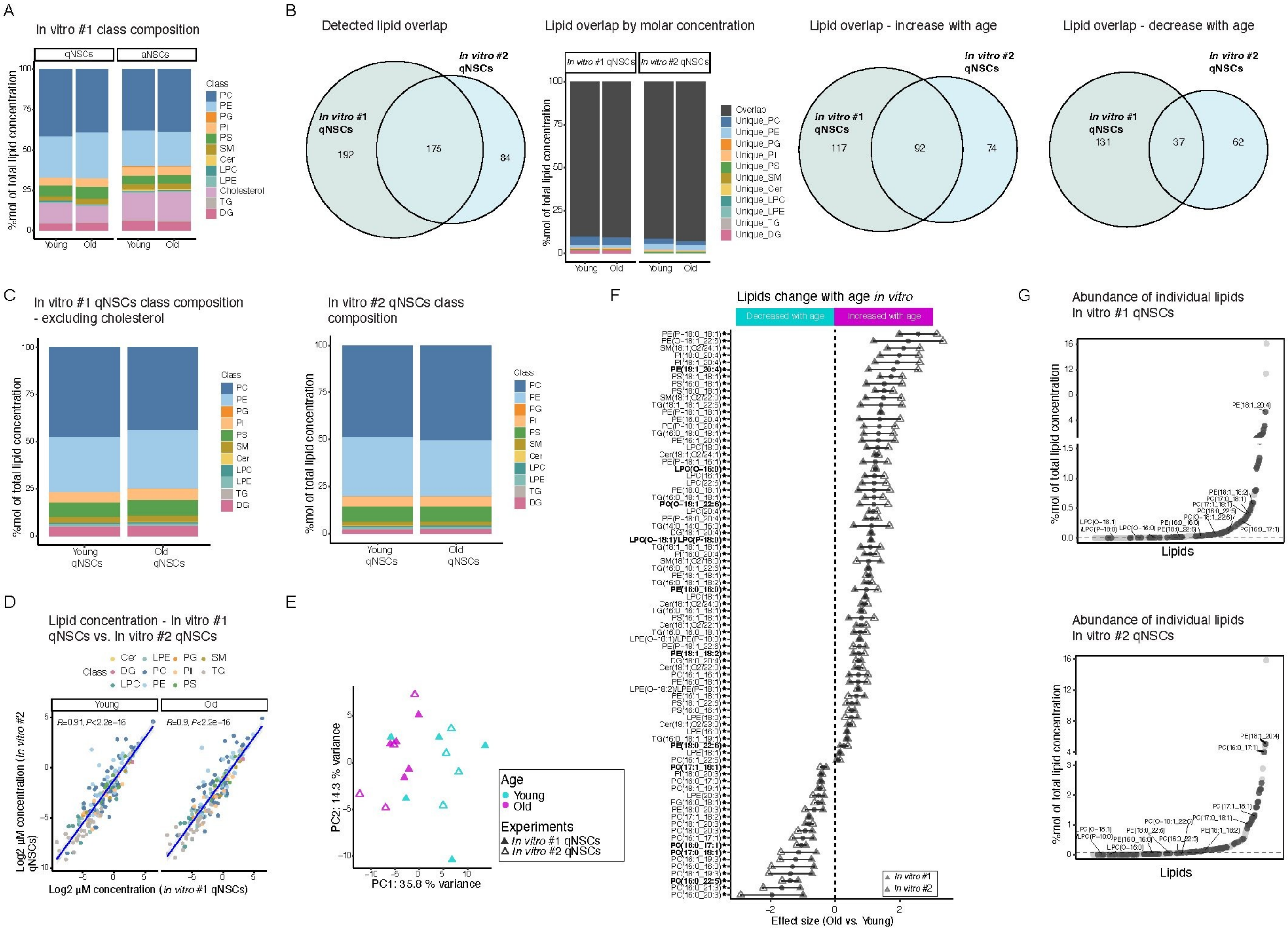

A
In vitro #1 class composition
qNSCs
aNSCs
%mol of total lipid concentration
Young
Old
Class
PC
PE
PG
PI
PS
SM
Cer
LPC
LPE
Cholesterol
TG
DG
B
Detected lipid overlap
In vitro #1 qNSCs
In vitro #2 qNSCs
192
175
84
Lipid overlap by molar concentration
In vitro #1 qNSCs
In vitro #2 qNSCs
Overlap
Unique_PC
Unique_PE
Unique_PG
Unique_PI
Unique_PS
Unique_SM
Unique_Cer
Unique_LPC
Unique_LPE
Unique_TG
Unique_DG
Lipid overlap - increase with age
117
92
74
Lipid overlap - decrease with age
131
37
62
C
In vitro #1 qNSCs class composition - excluding cholesterol
In vitro #2 qNSCs class composition
Young qNSCs
Old qNSCs
D
Lipid concentration - In vitro #1 qNSCs vs. In vitro #2 qNSCs
R=0.91, P<2.2e−16
R=0.9, P<2.2e−16
Log2 µM concentration (In vitro #2 qNSCs)
Log2 µM concentration (In vitro #1 qNSCs)
E
PC1: 35.8 % variance
PC2: 14.3 % variance
Age
Young
Old
Experiments
In vitro #1 qNSCs
In vitro #2 qNSCs
F
Lipids change with age in vitro
Decreased with age
Increased with age
Effect size (Old vs. Young)
In vitro #1
In vitro #2
G
Abundance of individual lipids In vitro #1 qNSCs
Abundance of individual lipids In vitro #2 qNSCs
%mol of total lipid concentration
Lipids

Fig. S1. Reproducibility of lipidomic changes at individual lipid level in primary neural stem cell cultures *in vitro* from independent lipidomic experiments.
(A) Concentration composition (%mol) of each lipid class on young and old qNSCs (left) and aNSCs (right) from *In vitro* #1 lipidomics. **(B)** Shared lipids from qNSCs of *In vitro* #1and *In vitro* #2. Venn diagram showing all overlapping lipids (left panel), lipids that increase with age (right-middle panel) and decrease with age (right panel) across 2 experiments. Lipid overlap by molar concentration is presented (left-middle panel) in a stacked bar plot, with individual block corresponding to the concentration composition (%mol) of commonly detected lipids across both datasets (dark gray block) and uniquely detected lipids from each dataset (colored blocks). **(C)** Concentration composition (%mol) of each lipid class on young and old qNSCs from *In vitro* #1 lipidomics excluding cholesterol (left) and *In vitro* #2 (right). **(D)** Correlation between lipid concentration (μM) in young (left panel) and old (right panel) qNSCs from *In vitro* #1 and *In vitro* #2. Pearson correlation was performed on concentration of individual lipids (solid dots colored by lipid classes) from each dataset. *R* and *P*-values from Pearson correlation are shown. Linear regression line (blue line) with 95% confidence interval (gray shade) is shown. **(E)** Principal component analysis (PCA) on log2 transformed lipid molar concentration of overlapping lipids from *In vitro* #1 (filled triangle) and *In vitro* #2 (empty triangle). PCA was performed on qNSCs only. Each symbol represents an individual primary qNSC culture established from individual young (cyan) or old (purple) mice. **(F)** Lipids change with age in qNSCs of *In vitro* #1 and *In vitro* #2. Significant lipids that change with age by effect size in both *in vitro* studies are plotted. Effect size for each lipid from *In vitro* #1 (filled triangle) and *In vitro* #2 (empty triangle) is shown together with mean effect size (black dot) and s.e.m. (error bar) from 2 studies. Statistical significance was assessed based on the *P*-value from test statistics using the 95% confidence interval of the data, and multiple hypothesis correction was done by FDR. Significance is identified when FDR-adjusted $P<0.05$ (see Methods for details). Black asterisk indicates significance. Bold labels indicate lipids of interest that significantly change with age and with *Mboat2* interventions (see **Fig. 4**). **(G)** Abundance of individual lipids across *in vitro* datasets. Dot plot of all detected lipids in *In vitro* #1 and *In vitro* #2 is ordered in %mol of total lipid concentration. Each dot represents an individual lipid species (light gray). Significant lipids with age from both studies (as listed in **Fig. 1E**) are highlighted in dark gray. Labeled lipids indicate lipids that significantly change with age (**Fig. 1**) and with *Mboat2* interventions (see **Fig. 6**, **Fig. 7**). Dashed line indicates median lipid abundance of each dataset.

Figure S2

A

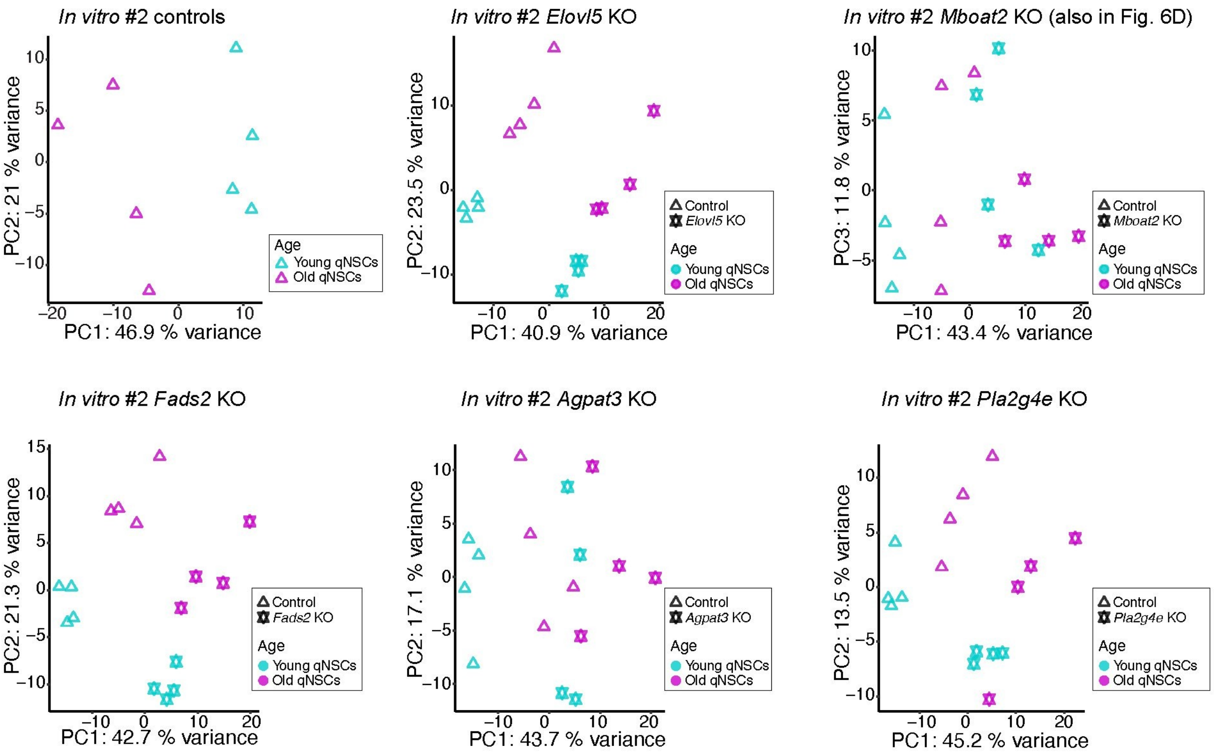

In vitro #2 controls
PC1: 46.9 % variance
PC2: 21 % variance
Age
Young qNSCs
Old qNSCs
In vitro #2 Elovl5 KO
PC1: 40.9 % variance
PC2: 23.5 % variance
Control
Elovl5 KO
In vitro #2 Mboat2 KO (also in Fig. 6D)
PC1: 43.4 % variance
PC3: 11.8 % variance
Mboat2 KO
In vitro #2 Fads2 KO
PC1: 42.7 % variance
PC2: 21.3 % variance
Fads2 KO
In vitro #2 Agpat3 KO
PC1: 43.7 % variance
PC2: 17.1 % variance
Agpat3 KO
In vitro #2 Pla2g4e KO
PC1: 45.2 % variance
PC2: 13.5 % variance
Pla2g4e KO

B

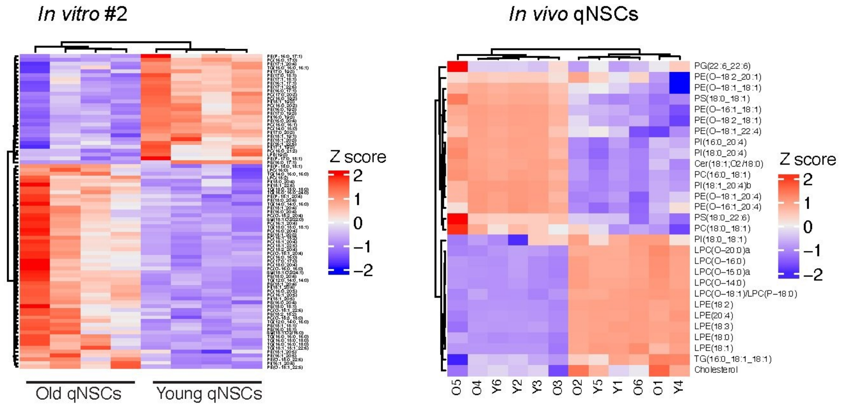

In vitro #2
Z score
Old qNSCs
Young qNSCs
In vivo qNSCs
PG(22:6_22:6)
PE(O-18:2_20:1)
PE(O-18:1_18:1)
PS(18:0_18:1)
PE(O-16:1_18:1)
PE(O-18:2_18:1)
PE(O-18:1_22:4)
PI(16:0_20:4)
PI(18:0_20:4)
Cer(18:1;O2/18:0)
PC(16:0_18:1)
PI(18:1_20:4)b
PE(O-18:1_20:4)
PE(O-16:1_20:4)
PS(18:0_22:6)
PC(18:0_18:1)
PI(18:0_18:1)
LPC(O-20:0)a
LPC(O-16:0)
LPC(O-15:0)a
LPC(O-14:0)
LPC(O-18:1)/LPC(P-18:0)
LPE(18:2)
LPE(20:4)
LPE(18:3)
LPE(18:0)
LPE(18:1)
TG(16:0_18:1_18:1)
Cholesterol
O5 O4 Y6 Y2 Y3 O3 O2 Y5 Y1 O6 O1 Y4

Fig. S2. Principal component analysis and heatmaps on lipids with significant change with age *in vitro* and all lipids from *in vivo* lipidomics experiments.

(A) Principal component analysis (PCA) on log2 transformed lipid concentration (*In vitro* #2). PCA on *Mboat2* KO from *In vitro* #2 is also shown in **Fig. 6D**. **(B)** Left: Heatmap with clustering on the Z score of lipids with significant change (FDR-adjusted $P<0.1$) with age in qNSCs of *In vitro* #2. Right: Heatmap with clustering on the Z score of all identified lipids from *in vivo* isolated qNSC lipidomics.

Figure S3

Odd chain lipids with the top 30% largest effect size in Fig. 1E and fig. S1F

PC(15:0_16:0) positive mode
In vitro #1

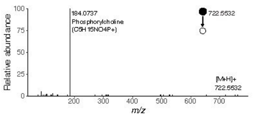

PC(15:0_16:0) negative mode
In vitro #1

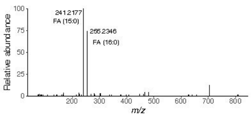

PC(15:0_16:0) positive mode
In vitro #2

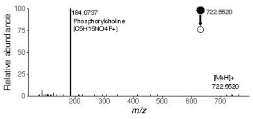

PC(15:0_16:0) negative mode
In vitro #2

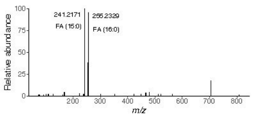

PC(18:1_19:3) positive mode
In vitro #1

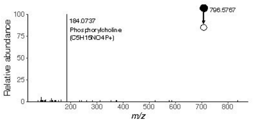

PC(18:1_19:3) negative mode
In vitro #1

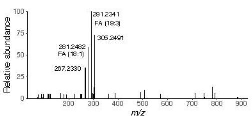

PC(18:1_19:3) positive mode
In vitro #2

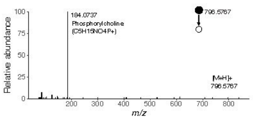

PC(18:1_19:3) negative mode
In vitro #2

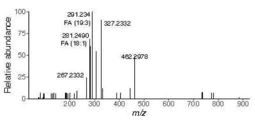

Odd chain lipids in Fig. 4B

PE(17:0_20:4) positive mode
In vitro #1

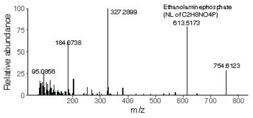

PE(17:0_20:4) negative mode
In vitro #1

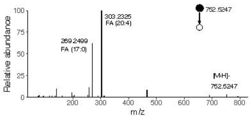

PE(17:0_20:4) positive mode
GPMV

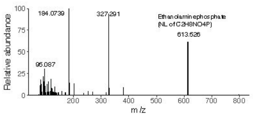

PE(17:0_20:4) negative mode
GPMV

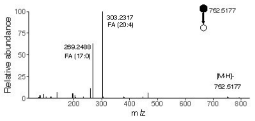

PE(P-18:1_17:1) positive mode
In vitro #1

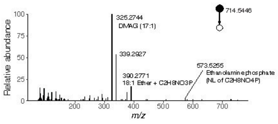

PE(P-18:1_17:1) positive mode
GPMV

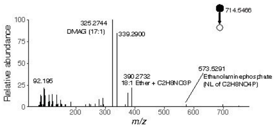

PS(17:0_18:1) positive mode
In vitro #1

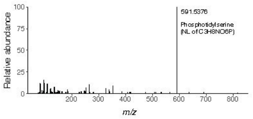

PS(17:0_18:1) negative mode
In vitro #1

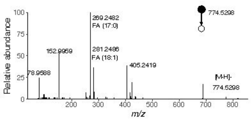

PS(17:0_18:1) positive mode
GPMV

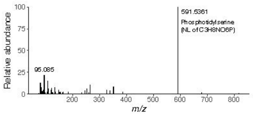

PS(17:0_18:1) negative mode
GPMV

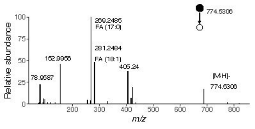

PE(17:0_18:1) positive mode
In vitro #1

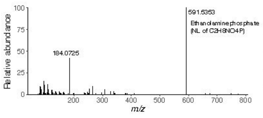

PE(17:0_18:1) negative mode
In vitro #1

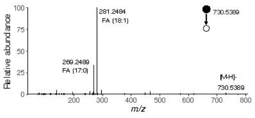

PE(17:0_18:1) positive mode
GPMV

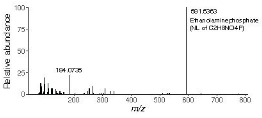

PE(17:0_18:1) negative mode
GPMV

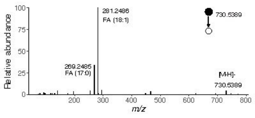

PC(16:0_19:3) positive mode
In vitro #1

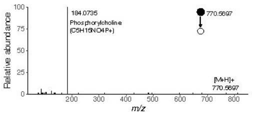

PC(16:0_19:3) negative mode
In vitro #1

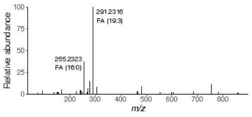

PC(16:0_19:3) positive mode
GPMV

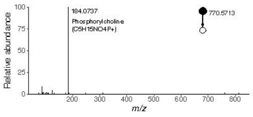

PC(16:0_19:3) negative mode
GPMV

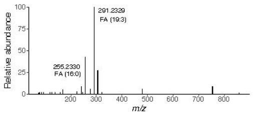

PC(16:1_19:3) positive mode
In vitro #1

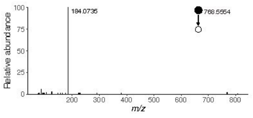

PC(16:1_19:3) negative mode
In vitro #1

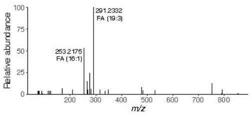

PC(16:1_19:3) positive mode
GPMV

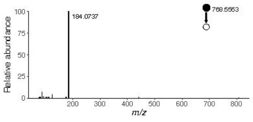

PC(16:1_19:3) negative mode
GPMV

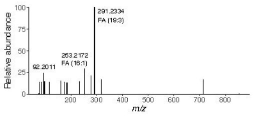

Fig. S3. Spectra validation on identified odd chain-containing lipids from 2 *in vitro* lipidomic studies and GPMV lipidomics.

Top: Tandem mass spectra of odd chain-containing lipids with the top 30% largest effect size in 2 *in vitro* lipidomic studies - PC(15:0_16:0) and PC(18:1_19:3). Bottom: Tandem mass spectra of odd chain-containing lipids in *In vitro* #1 and GPMV lipidomic studies - PE(17:0_20:4) and PE(P-18:1_17:1), PS(17:0_18:1), PE(17:0_18:1), PC(16:0_19:3), PC(16:1_19:3). Solid dot in all spectra represents molecular mass used to identify each molecule (precursor ion).

Figure S4

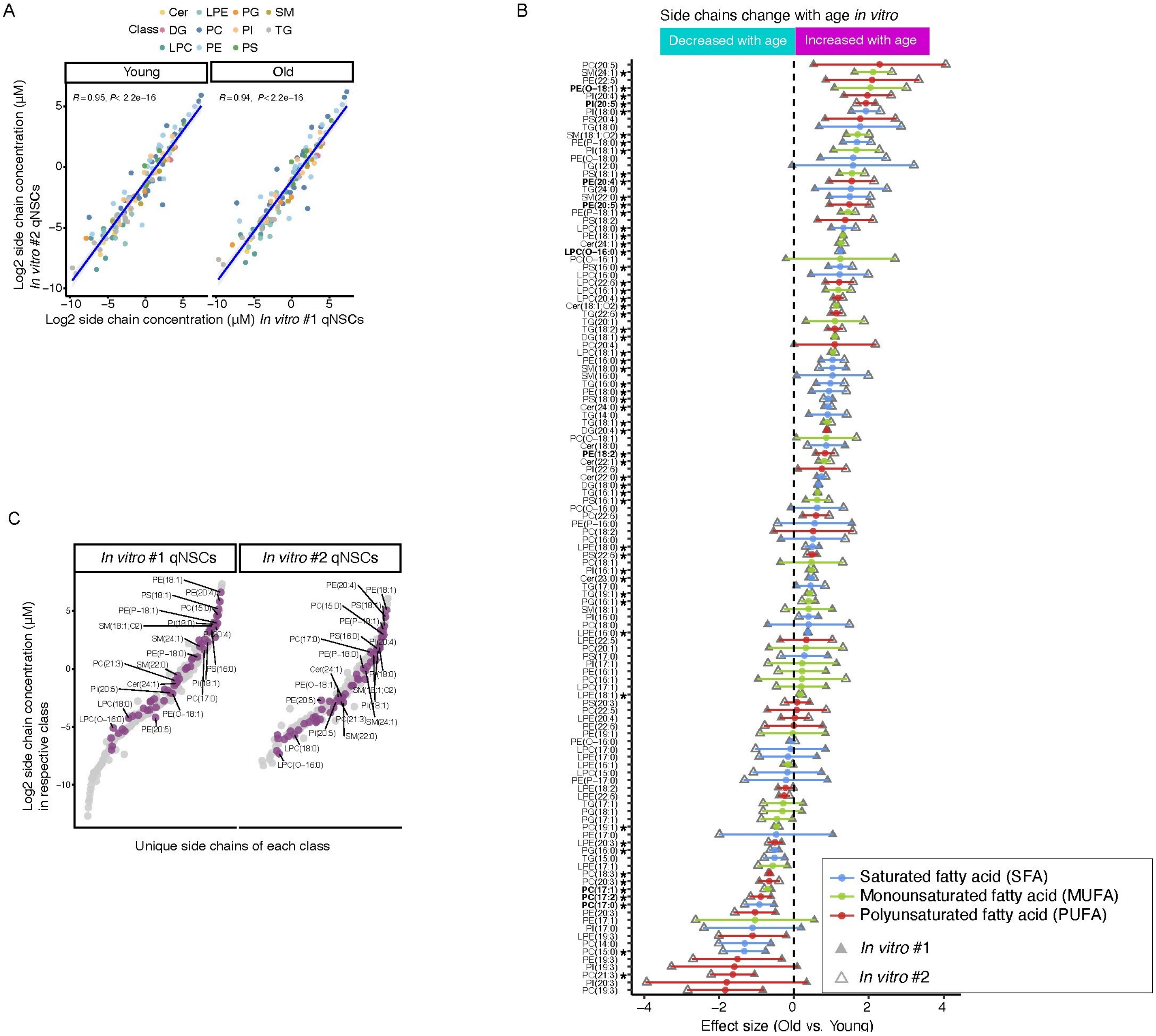
A
Class
Cer
LPE
PG
SM
DG
PC
PI
TG
LPC
PE
PS
Young
Old
R = 0.95, P < 2.2e−16
R = 0.94, P < 2.2e−16
Log2 side chain concentration (μM)
In vitro #2 qNSCs
Log2 side chain concentration (μM) In vitro #1 qNSCs
B
Side chains change with age in vitro
Decreased with age
Increased with age
Saturated fatty acid (SFA)
Monounsaturated fatty acid (MUFA)
Polyunsaturated fatty acid (PUFA)
In vitro #1
In vitro #2
Effect size (Old vs. Young)
C
In vitro #1 qNSCs
In vitro #2 qNSCs
Log2 side chain concentration (μM)
in respective class
Unique side chains of each class

Fig. S4. Reproducibility of lipidomic changes at side chain composition level in primary neural stem cell cultures *in vitro* from independent lipidomic experiments.

(A) Correlation between side chain composition in young (left panel) and old (right panel) qNSCs between *In vitro* #1 and *In vitro* #2. Pearson correlation was performed on side chain abundance in log2-transformed molar concentration of each lipid class/side chain (solid dots colored by lipid classes) from each dataset. *R* and *P*-values from Pearson correlation are shown. Linear regression line (blue line) with 95% confidence interval (gray shade) is shown. **(B)** Side chain composition changes with age in qNSCs from *In vitro* #1 and *In vitro* #2. All side chain composition features from both studies are plotted. Effect size for changes with age of each feature from *In vitro* #1 (filled triangle) and *In vitro* #2 (empty triangle) is shown together with mean effect size (solid dot) in blue, green and red that represents saturated fatty acid (SFA), monounsaturated fatty acid (MUFA), and polyunsaturated fatty acid (PUFA), respectively. Error bar represents s.e.m. from 2 studies. Statistical significance was assessed based on the *P*-value from test statistics using the 95% confidence interval of the data, and multiple hypothesis correction was done by FDR. Significance is identified when FDR-adjusted $P<0.05$ (see Methods for details). Black asterisk indicates significance. **(C)** Abundance of side chain features with significant change across *in vitro* datasets. Dot plot of side chain features detected in *In vitro* #1 and *In vitro* #2 ordered in molar concentration. Each dot represents a unique lipid class/side chain combination. Significant side chain composition features from both *in vitro* studies are highlighted in purple. Labeled features indicate side chain features that exhibit the top 30% most significant effect size (either increase or decrease with age).

Figure S5

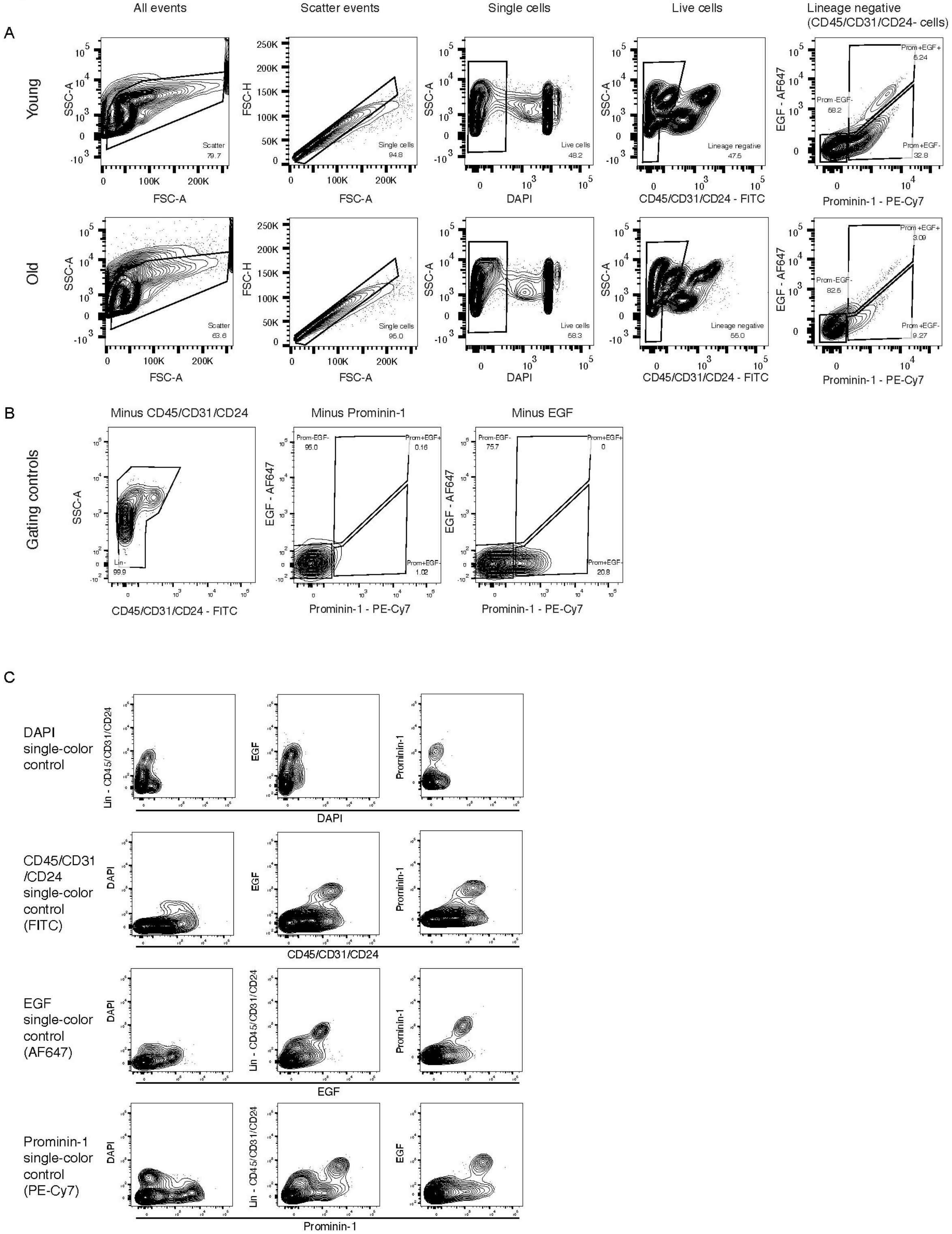

A
All events
Scatter events
Single cells
Live cells
Lineage negative (CD45/CD31/CD24- cells)
Young
Old
SSC-A
FSC-A
FSC-H
DAPI
CD45/CD31/CD24 - FITC
EGF - AF647
Prominin-1 - PE-Cy7
Scatter 79.7
Single cells 94.8
Live cells 48.2
Lineage negative 47.5
Prom-EGF- 58.2
Prom+EGF+ 5.24
Prom+EGF- 32.8
Scatter 63.6
Single cells 95.0
Live cells 58.3
Lineage negative 55.0
Prom-EGF- 82.5
Prom+EGF+ 3.09
Prom+EGF- 9.27
B
Gating controls
Minus CD45/CD31/CD24
Minus Prominin-1
Minus EGF
Lin- 99.9
Prom-EGF- 95.0
Prom+EGF+ 0.16
Prom+EGF- 1.02
Prom-EGF- 75.7
Prom+EGF+ 0
Prom+EGF- 20.8
C
DAPI single-color control
CD45/CD31/CD24 single-color control (FITC)
EGF single-color control (AF647)
Prominin-1 single-color control (PE-Cy7)
Lin - CD45/CD31/CD24
EGF
Prominin-1
DAPI
CD45/CD31/CD24

Fig. S5. FACS gating scheme of freshly isolated qNSCs.
(A) FACS gating strategy used to freshly isolate quiescent NSCs from the SVZ neurogenic niches of young and old mice. **(B)** Negative gating controls for each staining are indicated. For gating controls, cells were stained with all antibodies except the one for which it is a negative control. **(C)** Single-color staining controls of DAPI, CD45/CD31/CD24 (FITC), EGF (AF647) and Prominin-1 (PE-Cy7) are shown after spectral compensation.

Figure S6

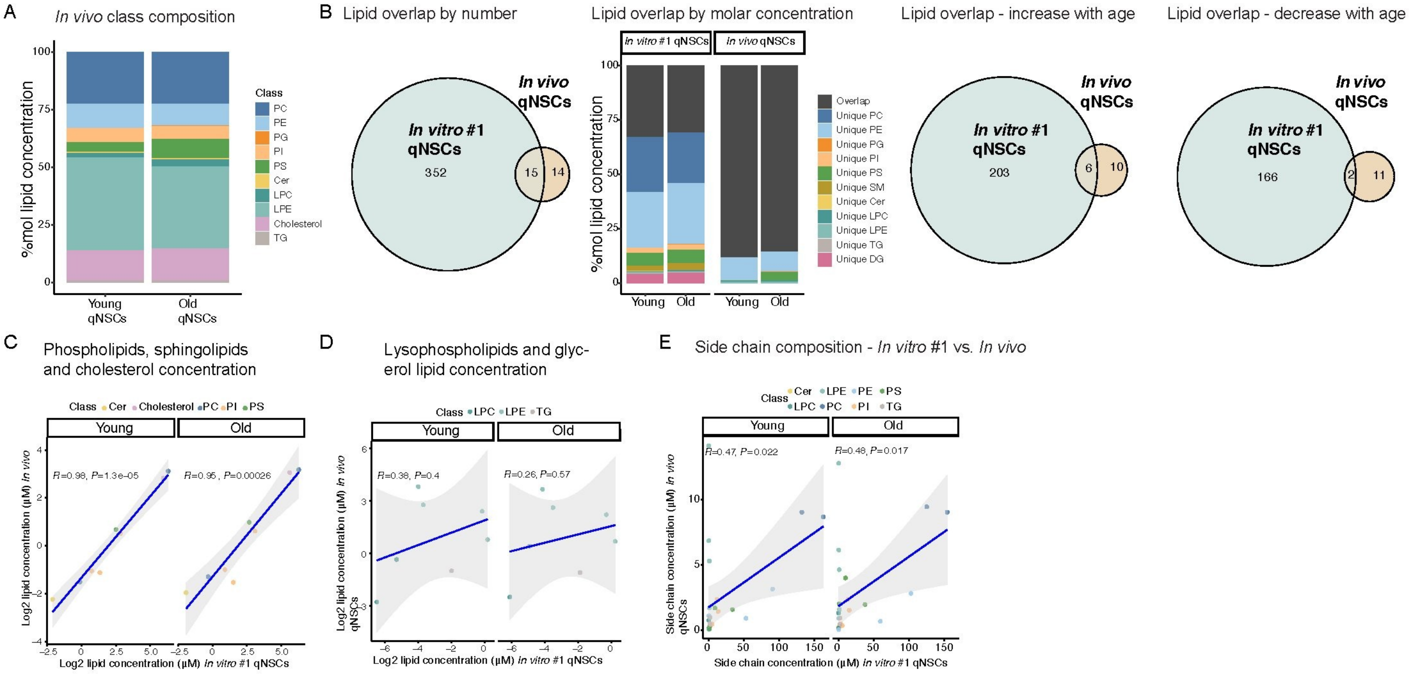
A
In vivo class composition
%mol lipid concentration
Class
PC
PE
PG
PI
PS
Cer
LPC
LPE
Cholesterol
TG
Young qNSCs
Old qNSCs
B
Lipid overlap by number
In vitro #1 qNSCs
In vivo qNSCs
352
15
14
Lipid overlap by molar concentration
In vitro #1 qNSCs
In vivo qNSCs
Overlap
Unique PC
Unique PE
Unique PG
Unique PI
Unique PS
Unique SM
Unique Cer
Unique LPC
Unique LPE
Unique TG
Unique DG
Young
Old
Lipid overlap - increase with age
203
6
10
Lipid overlap - decrease with age
166
2
11
C
Phospholipids, sphingolipids and cholesterol concentration
Class Cer Cholesterol PC PI PS
Young
Old
R=0.98, P=1.3e-05
R=0.95 , P=0.00026
Log2 lipid concentration (µM) In vivo
Log2 lipid concentration (µM) In vitro #1 qNSCs
D
Lysophospholipids and glycerol lipid concentration
Class LPC LPE TG
R=0.38, P=0.4
R=0.26, P=0.57
Log2 lipid concentration (µM) In vivo qNSCs
Log2 lipid concentration (µM) In vitro #1 qNSCs
E
Side chain composition - In vitro #1 vs. In vivo
Class Cer LPE PE PS LPC PC PI TG
R=0.47, P=0.022
R=0.48, P=0.017
Side chain concentration (µM) In vivo qNSCs
Side chain concentration (µM) In vitro #1 qNSCs

Fig. S6. Lipidomic changes of *in vivo* isolated qNSCs compared to *in vitro* qNSC primary cultures. **(A)** Concentration composition (%mol) of each lipid class on young and old samples from *in vivo* isolated qNSCs. **(B)** Shared lipids from qNSCs *in vitro* and *in vivo*. Venn diagram showing all overlapping lipids (left panel), lipids that increase with age (right-middle panel) and decrease with age (right panel) from *in vitro* #1 and *in vivo* lipidomics. Lipid overlap by molar concentration is presented (left-middle panel) in a stacked bar plot, with individual block corresponding to the concentration composition (%mol) of commonly detected lipids from *in vitro* #1 and *in vivo* (dark gray block) and uniquely detected lipids from each dataset (colored blocks). **(C to D)** Correlation between phospholipids, sphingolipids and cholesterol concentration (**C**) and lysophospholipids and glycerol lipid concentration (**D**) (μM) in young (left panel) and old (right panel) qNSCs from *in vitro* #1 and *in vivo*. Pearson correlation was performed on concentration of individual lipids (solid dots colored by lipid classes) from each dataset. *R* and *P*-values from Pearson correlation are shown. Linear regression line (blue line) with 95% confidence interval (gray shade) is shown. **(E)** Correlation between side chain composition in young (left panel) and old (right panel) qNSCs between *in vitro* #1 and *in vivo*. Pearson correlation was performed on side chain abundance (in molar concentration) of each lipid class/ side chain (solid dots colored by lipid classes) from each dataset. *R* and *P*-values from Pearson correlation are shown. Linear regression line (blue line) with 95% confidence interval (gray shade) is shown.

Figure S7

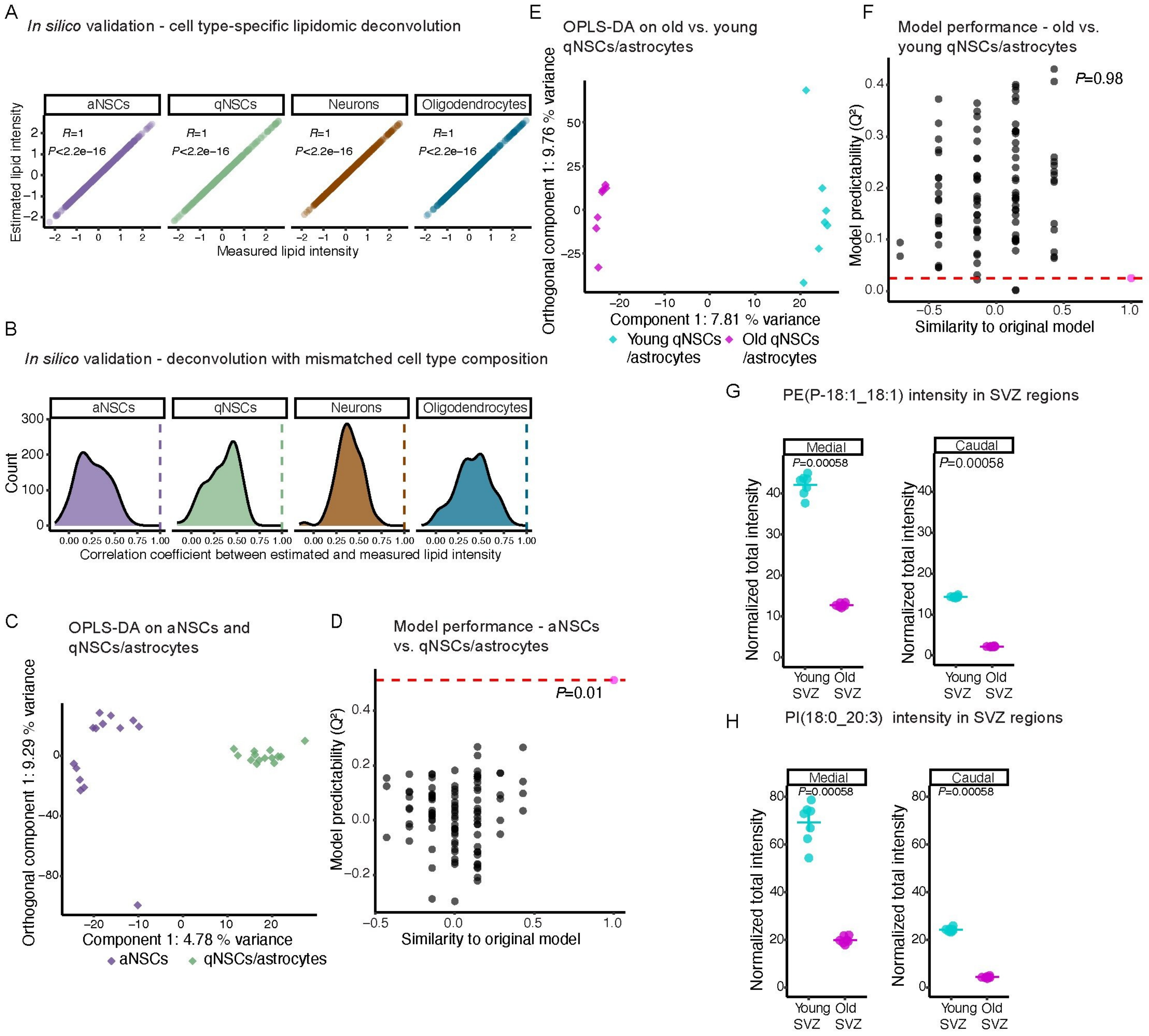
A
In silico validation - cell type-specific lipidomic deconvolution
aNSCs
qNSCs
Neurons
Oligodendrocytes
R=1
P<2.2e−16
Estimated lipid intensity
Measured lipid intensity
B
In silico validation - deconvolution with mismatched cell type composition
Count
Correlation coefficient between estimated and measured lipid intensity
C
OPLS-DA on aNSCs and qNSCs/astrocytes
Orthogonal component 1: 9.29 % variance
Component 1: 4.78 % variance
aNSCs
qNSCs/astrocytes
D
Model performance - aNSCs vs. qNSCs/astrocytes
P=0.01
Model predictability (Q²)
Similarity to original model
E
OPLS-DA on old vs. young qNSCs/astrocytes
Orthogonal component 1: 9.76 % variance
Component 1: 7.81 % variance
Young qNSCs /astrocytes
Old qNSCs /astrocytes
F
Model performance - old vs. young qNSCs/astrocytes
P=0.98
Model predictability (Q²)
Similarity to original model
G
PE(P-18:1_18:1) intensity in SVZ regions
Medial
Caudal
P=0.00058
Normalized total intensity
Young SVZ
Old SVZ
H
PI(18:0_20:3) intensity in SVZ regions
Medial
Caudal
P=0.00058
Normalized total intensity
Young SVZ
Old SVZ

Fig. S7. Metabolomic profiling on young and old subventricular zone neurogenic niches by DESI-MSI.
(A) Cell type-specific deconvolution from *in silico* mixture lipidomic data. Pearson correlation was performed between the Z score of measured lipid intensity and Z score of estimated lipid intensity of activated NSC (aNSC), quiescent NSC (qNSC), neurons and oligodendrocytes from a reconstituted mixture lipidomic dataset (see Methods for details). Each dot represents a lipid species detected from an individual culture of brain cell type. *R* and *P*-values from Pearson correlation are shown. **(B)** Deconvolution from *in silico* mixture lipidomic data with mismatched cell type proportions using randomly-generated cell type compositions (see Methods for details). Density plot shows the number of occurrence (Y axis) of the Pearson correlation coefficient (X axis) of each lipid species between measured and estimated lipid intensity for each individual cell type after deconvolution. Dashed line indicates the correlation coefficient obtained from deconvolution when the matching cell proportions were used (same as panel a). **(C and E)** Orthogonal partial least squares-discriminant analysis (OPLS-DA) on DESI-MSI metabolomic profiling of SVZ cells from n=7 young (3-5 months) and n=7 old (20-22 months) C57BL/6JN male mice. OPLS-DA was performed to obtain cell type-specific metabolic signatures between aNSCs and qNSCs/astrocytes (**C**), and age-related metabolic signatures between young and old qNSCs/astrocytes (**E**). Each data point represents a deconvolved metabolomic profile of a specific cell type from an individual young or old mouse. Data points are plotted based on the predictive score (Component 1, X axis) and the orthogonal predictive score (Orthogonal component 1, Y axis). **(D and F)** Predictability of OPLS-DA model was assessed by permutation test. Similarity to the original model (X axis) and model predictability index (Q^2) (Y axis) from the original model (pink dot on the right of the red dashed line) and individual permutation tests (black dots) are plotted for OPLS-DA model identifying cell type-specific metabolic signatures (**D**) and OPLS-DA model identifying age-related metabolic signatures between young and old qNSCs/astrocytes (**F**). *P*-values were calculated on Q^2 between the original and permutated models. Red dashed line represents the Q^2 index from the original model. **(G and H)** Quantification of the normalized lipid intensity in the medial (left) and caudal (right) region of the SVZ on PE(P-18:1_18:1) (**G**) and PI(18:0_20:3) (**H**). Dot and whisker plot with each individual dot representing the normalized intensity of specified lipids in the medial (left) and caudal (right) region of SVZ from an individual animal. Whisker: +/- s.e.m.. *P*-values from Wilcoxon rank-sum test.

Figure S8

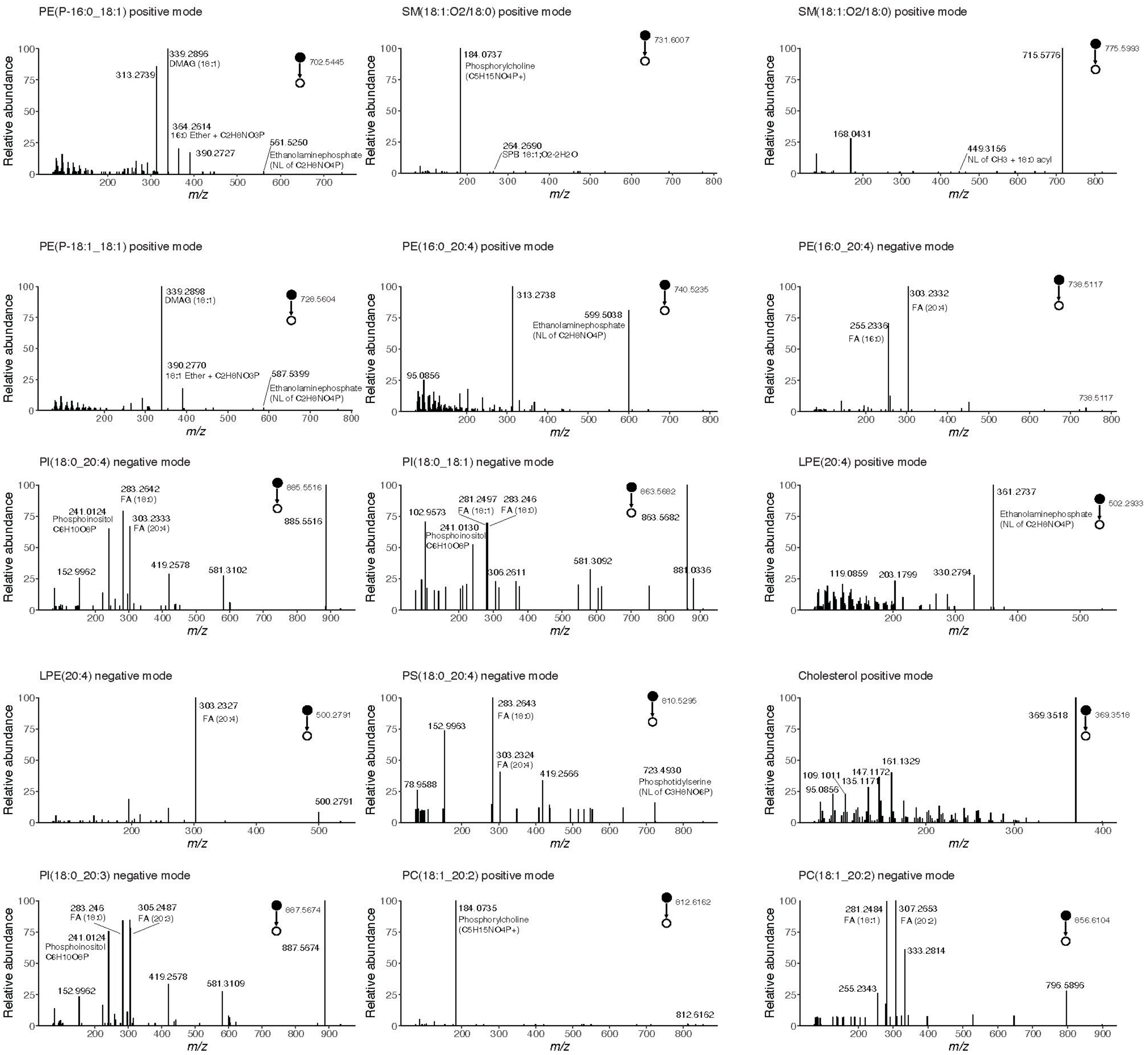
PE(P-16:0_18:1) positive mode
702.5445
339.2896
DMAG (18:1)
313.2739
364.2614
16:0 Ether + C2H8NO3P
390.2727
561.5250
Ethanolaminephosphate
(NL of C2H8NO4P)
SM(18:1:O2/18:0) positive mode
731.6007
184.0737
Phosphorylcholine
(C5H15NO4P+)
264.2690
SPB 18:1;O2-2H2O
SM(18:1:O2/18:0) positive mode
775.5993
715.5776
168.0431
449.3156
NL of CH3 + 18:0 acyl
PE(P-18:1_18:1) positive mode
728.5604
339.2898
DMAG (18:1)
390.2770
18:1 Ether + C2H8NO3P
587.5399
Ethanolaminephosphate
(NL of C2H8NO4P)
PE(16:0_20:4) positive mode
740.5235
313.2738
599.5038
Ethanolaminephosphate
(NL of C2H8NO4P)
95.0856
PE(16:0_20:4) negative mode
738.5117
303.2332
FA (20:4)
255.2336
FA (16:0)
738.5117
PI(18:0_20:4) negative mode
885.5516
283.2642
FA (18:0)
241.0124
Phosphoinositol
C6H10O8P
303.2333
FA (20:4)
885.5516
152.9962
419.2578
581.3102
PI(18:0_18:1) negative mode
863.5682
281.2497
FA (18:1)
283.246
FA (18:0)
102.9573
241.0130
Phosphoinositol
C6H10O8P
863.5582
581.3092
306.2611
881.0336
LPE(20:4) positive mode
502.2933
361.2737
Ethanolaminephosphate
(NL of C2H8NO4P)
119.0859
203.1799
330.2794
LPE(20:4) negative mode
500.2791
303.2327
FA (20:4)
500.2791
PS(18:0_20:4) negative mode
810.5295
283.2643
FA (18:0)
152.9963
303.2324
FA (20:4)
419.2566
723.4930
Phosphotidylserine
(NL of C3H8NO6P)
78.9588
Cholesterol positive mode
369.3518
369.3518
161.1329
109.1011
147.1172
95.0856
135.1171
PI(18:0_20:3) negative mode
887.5674
283.246
FA (18:0)
305.2487
FA (20:3)
241.0124
Phosphoinositol
C6H10O8P
887.5674
419.2578
581.3109
152.9962
PC(18:1_20:2) positive mode
812.6162
184.0735
Phosphorylcholine
(C5H15NO4P+)
812.6162
PC(18:1_20:2) negative mode
856.6104
281.2484
FA (18:1)
307.2653
FA (20:2)
333.2814
255.2343
796.5896
Relative abundance
m/z

Fig. S8. Spectra validation on identified lipids in DESI-MSI.

Tandem mass spectrometry validation following DESI-MSI. Validation of lipid annotation by tandem mass spectrometry for PE(P-16:0_18:1), SM(18:1;O2/18:0), PE(P-18:1_18:1), PE(16:0_20:4), PI(18:0_20:4), PI(18:0_18:1), LPE(20:4), PS(18:0_20:4), Cholesterol, PI(18:0_20:3) and PC(18:1_20:2). Solid dot in all spectra represents molecular mass used to identify each molecule (precursor ion).

Figure S9

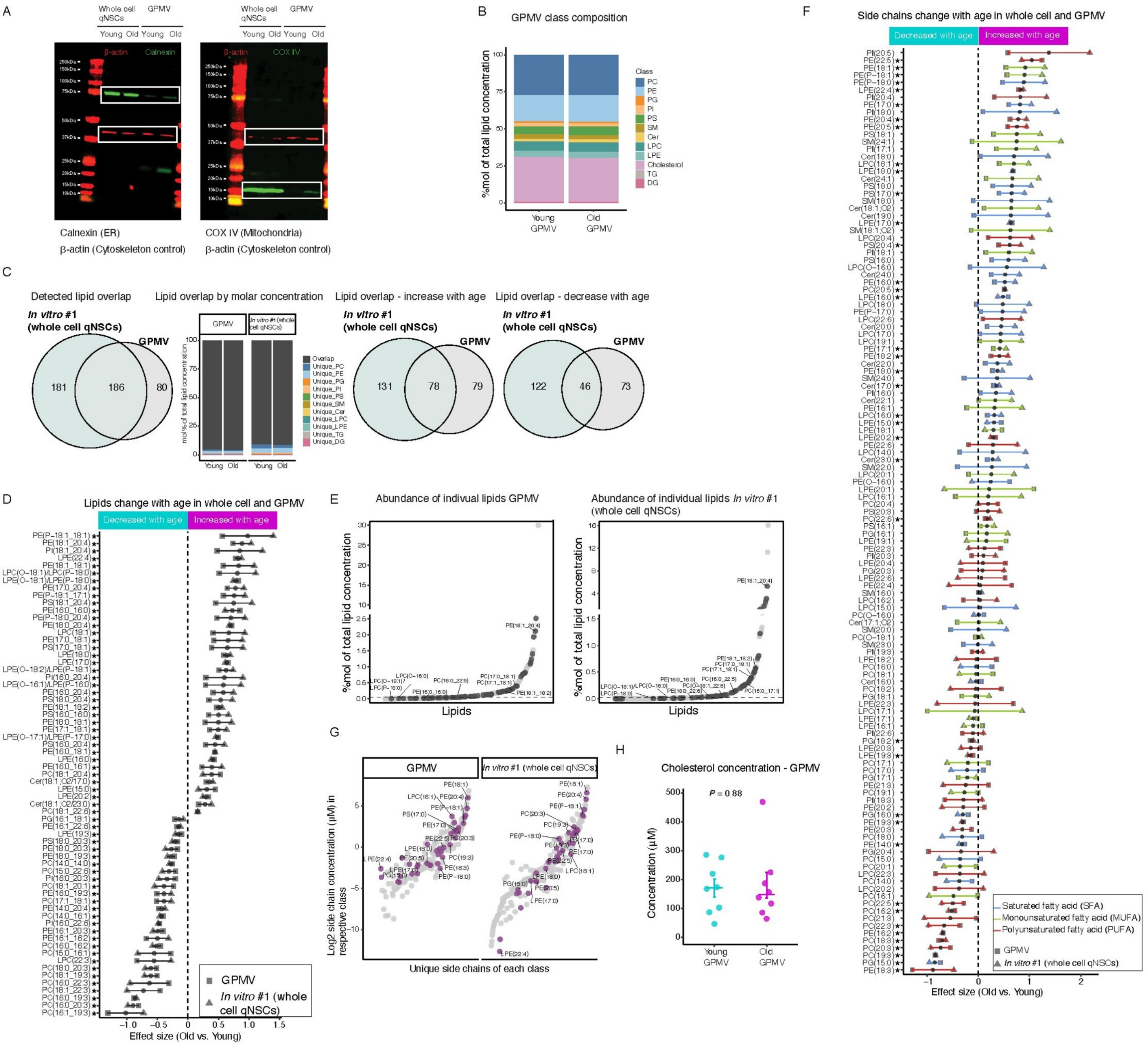

A
Whole cell qNSCs
GPMV
Young Old Young Old
β-actin
Calnexin
COX IV
Calnexin (ER)
β-actin (Cytoskeleton control)
COX IV (Mitochondria)
β-actin (Cytoskeleton control)
B
GPMV class composition
%mol of total lipid concentration
Class
PC
PE
PG
PI
PS
SM
Cer
LPC
LPE
Cholesterol
TG
DG
Young GPMV
Old GPMV
C
Detected lipid overlap
In vitro #1 (whole cell qNSCs)
GPMV
181
186
80
Lipid overlap by molar concentration
mol% of total lipid concentration
Overlap
Unique_PC
Unique_PE
Unique_PG
Unique_PI
Unique_PS
Unique_SM
Unique_Cer
Unique_LPC
Unique_LPE
Unique_TG
Unique_DG
Lipid overlap - increase with age
131
78
79
Lipid overlap - decrease with age
122
46
73
D
Lipids change with age in whole cell and GPMV
Decreased with age
Increased with age
GPMV
In vitro #1 (whole cell qNSCs)
Effect size (Old vs. Young)
E
Abundance of indivual lipids GPMV
Abundance of individual lipids In vitro #1 (whole cell qNSCs)
%mol of total lipid concentration
Lipids
G
GPMV
In vitro #1 (whole cell qNSCs)
Log2 side chain concentration (μM) in respective class
Unique side chains of each class
H
Cholesterol concentration - GPMV
P = 0.88
Concentration (μM)
Young GPMV
Old GPMV
F
Side chains change with age in whole cell and GPMV
Decreased with age
Increased with age
Saturated fatty acid (SFA)
Monounsaturated fatty acid (MUFA)
Polyunsaturated fatty acid (PUFA)
GPMV
In vitro #1 (whole cell qNSCs)
Effect size (Old vs. Young)

Fig. S9. Plasma membrane lipids change with age in quiescent neural stem cells.
(A) Western blot on protein extracts from whole cell lysates or giant plasma membrane vesicles (GPMVs) generated from primary qNSC cultures from young (3-5 months) and old (20-22 months) mice. Membranes were incubated with antibodies to cytoskeleton control β-actin (red), endoplasmic reticulum marker Calnexin (green), and mitochondria marker COX IV (green). **(B)** Concentration composition (%mol) of each lipid class on GPMVs from young and old primary qNSC cultures. **(C)** Shared lipids from qNSCs of GPMV and *In vitro* #1 lipidomics. Venn diagram showing all overlapping lipids (left panel), lipids that increase with age (right-middle panel) and decrease with age (right panel) in GPMV and *In vitro* #1. Lipid overlap by molar concentration is presented (left-middle panel) in a stacked bar plot, with individual block corresponding to the concentration composition (%mol) of commonly detected lipids in GPMV and *In vitro* #1 (dark grey block) and uniquely detected lipids from each dataset (colored blocks). **(D)** Lipids change with age in qNSCs of GPMV and *In vitro* #1. Significant lipids that change with age by effect size in GPMV and *In vitro* #1 are plotted. Effect size for each lipid from *In vitro* #1 (filled triangle) and GPMV (square) is shown together with mean effect size (black dot) and s.e.m. (error bar) from 2 studies. Significance is identified when FDR-adjusted $P<0.05$ (see Methods for details). Black asterisk indicates significance. **(E)** Abundance of individual lipids in GPMV and *In vitro* #1. Dot plot of all detected lipids in GPMV and *In vitro* #1 is ordered in %mol of total lipid concentration. Each dot represents an individual lipid species (light grey). Significant lipids from both studies (as listed in **D**) are highlighted in dark grey. Text label indicates lipids of interest that significantly change with age (**Fig. 1**) and with intervention (**Fig. 6, Fig. 7**). Dashed line indicates median lipid abundance of each dataset. **(F)** Side chain composition changes with age in whole cell and GPMV from qNSC primary cultures. Effect size on changes with age of side chain composition features from GPMV (square) and *In vitro* #1 (filled triangle) is plotted together with mean effect size (solid dot) in blue, green and red that represents saturated fatty acid (SFA), monounsaturated fatty acid (MUFA), and polyunsaturated fatty acid (PUFA), respectively. Error bar represents s.e.m. from GPMV and *In vitro* #1. Significance is identified when FDR-adjusted $P<0.05$ (see Methods for details). Black asterisk indicates significance. For statistical test of age-related effect sizes on lipids (**D**) and side chain compositions (**F**), statistical significance was assessed based on the *P*-value from test statistics using the 95% confidence interval of the data, and multiple hypothesis correction was done by FDR. **(G)** Abundance of side chain features with significant changes in GPMV and *In vitro* #1. Dot plot of side chain composition features detected in GPMV and *In vitro* #1 is ordered in molar concentration. Each dot represents a unique lipid class/side chain combination. Significant side chain composition features from both studies (as listed in **Fig. 4C**) are highlighted in purple. Labeled features indicate side chain features that exhibit the top 30% most significant effect size (either increase or decrease with age). **(H)** Cholesterol concentration from plasma membrane of young and old qNSCs. Cholesterol concentration from GPMV lipidomics of primary qNSC cultures of n=8 young (3-5 months) and n=8 old (20-22 months) mice. Each dot represents a GPMV sample, generated from a primary qNSC culture that was established with an individual mouse. Bar plot represents mean +/- s.e.m.. *P*-value from Wilcoxon rank-sum test.

Figure S10

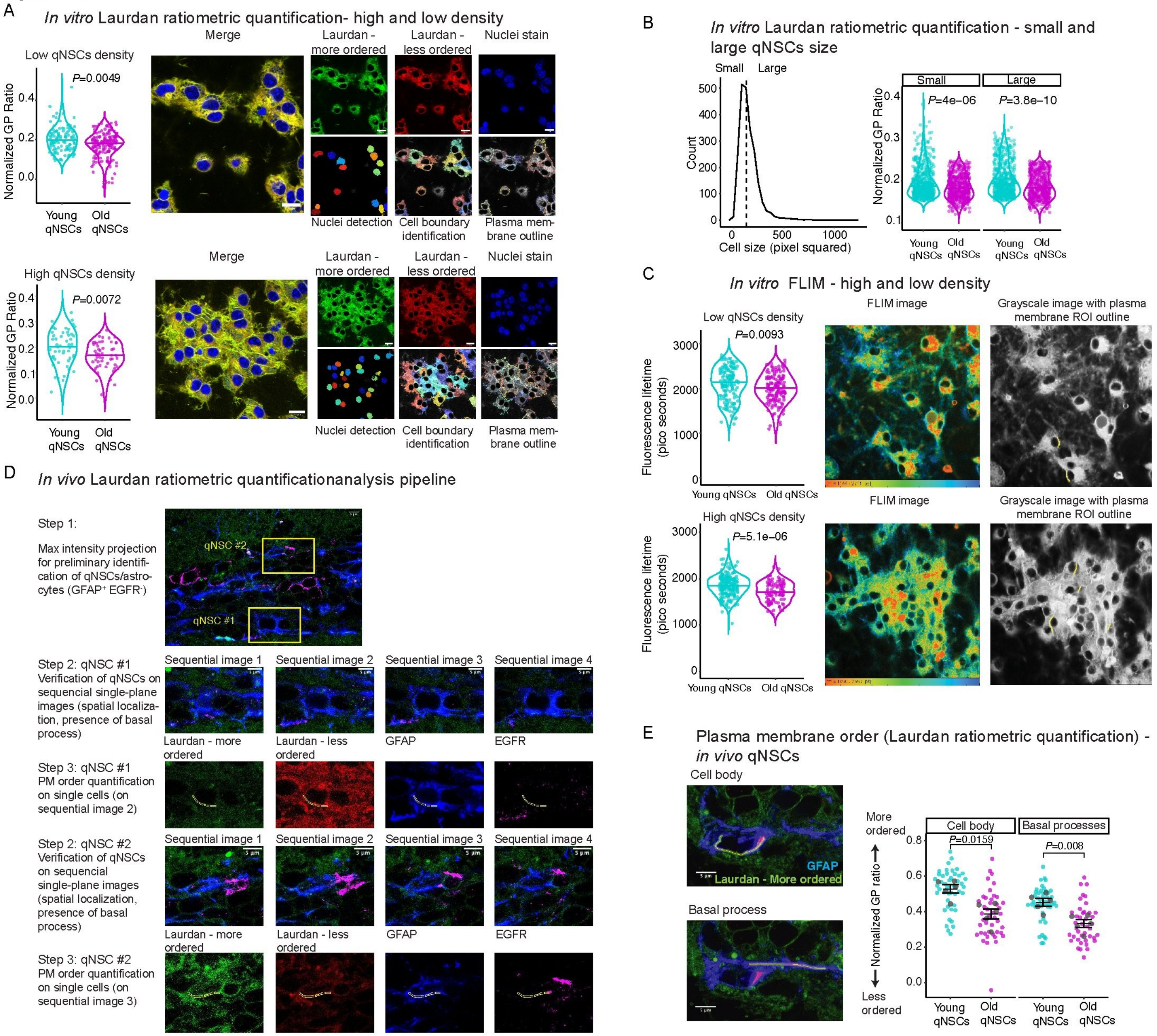
A
In vitro Laurdan ratiometric quantification- high and low density
Low qNSCs density
P=0.0049
Normalized GP Ratio
Young qNSCs
Old qNSCs
Merge
Laurdan - more ordered
Laurdan - less ordered
Nuclei stain
Nuclei detection
Cell boundary identification
Plasma membrane outline
High qNSCs density
P=0.0072
B
In vitro Laurdan ratiometric quantification - small and large qNSCs size
Small
Large
Count
Cell size (pixel squared)
P=4e−06
P=3.8e−10
C
In vitro FLIM - high and low density
Low qNSCs density
P=0.0093
Fluorescence lifetime (pico seconds)
FLIM image
Grayscale image with plasma membrane ROI outline
High qNSCs density
P=5.1e−06
D
In vivo Laurdan ratiometric quantificationanalysis pipeline
Step 1:
Max intensity projection for preliminary identification of qNSCs/astrocytes (GFAP+ EGFR-)
qNSC #2
qNSC #1
Step 2: qNSC #1
Verification of qNSCs on sequencial single-plane images (spatial localization, presence of basal process)
Sequential image 1
Sequential image 2
Sequential image 3
Sequential image 4
Step 3: qNSC #1
PM order quantification on single cells (on sequential image 2)
Laurdan - more ordered
Laurdan - less ordered
GFAP
EGFR
Step 2: qNSC #2
Verification of qNSCs on sequencial single-plane images (spatial localization, presence of basal process)
Step 3: qNSC #2
PM order quantification on single cells (on sequential image 3)
E
Plasma membrane order (Laurdan ratiometric quantification) - in vivo qNSCs
Cell body
GFAP
Laurdan - More ordered
Basal process
More ordered
Less ordered
Normalized GP ratio
Basal processes
P=0.0159
P=0.008

Fig. S10. Plasma membrane order change with age in qNSCs *in vitro* and *in vivo*.
(A) Plasma membrane order assessed *in vitro* by Laurdan staining in low cell density (top) and high cell density (bottom). Left: Plasma membrane order quantified by generalized polarization ratio (GP ratio) from Laurdan staining of young and old qNSCs. Violin plot showing data from individual cells (cyan/purple dots). Results from one independent experiment, n=4 primary cultures. *P*-value from Wilcoxon rank-sum test. Middle: Image from Laurdan rigid wavelength (more ordered, green) overlaid with Laurdan fluid wavelength (less ordered, red) and nuclei staining (blue) is shown. Right: Confocal images captured from individual wavelength (top) and steps of automated image analysis pipeline for plasma membrane order quantification (bottom). Scale bar represents 10μm. **(B)** Plasma membrane order assessed *in vitro* by Laurdan staining based on cell size. Left: Cell size distribution of individual qNSCs from primary young and old qNSC culture. Vertical dashed line indicates the median size of all cells quantified. Right: Plasma membrane order quantified by generalized polarization ratio (GP ratio) from Laurdan staining of young and old qNSCs. Violin plot showing data from individual cells (cyan/purple dots) that are either smaller or larger than the median cell size. Results from one independent experiment, n=4 primary cultures. *P*-values from Wilcoxon rank-sum test. **(C)** Plasma membrane order assessed *in vitro* by FLIM imaging in low cell density (top) and high cell density (bottom). Left: Plasma membrane order quantified by FLIM imaging of young and old qNSCs. Violin plots showing data from individual cells (cyan/purple dots). Results from two experiments combined, n=6 primary cultures. *P*-values from Wilcoxon rank-sum test. Middle: Representative FLIM image. Color scale corresponds to fluorescent lifetime throughout the cell. Right: Grayscale image with plasma membrane outlined in yellow for plasma membrane order quantification. **(D)** Additional examples of *in vivo* qNSC plasma membrane order quantification by Laurdan staining and ratiometric quantification. Row 1: Max intensity projection from a Z stack of confocal images showing Laurdan rigid wavelength (more ordered, green) overlaid with GFAP (NSC and astrocyte marker, blue) and EGFR (proliferation marker, purple) staining. Two individual qNSCs (qNSC #1 and #2), based on markers ($GFAP^{+}EGFR^{-}$), spatial localization (immediately adjacent to ependymal cells that line the lateral ventricles) and the presence of a basal process, are highlighted for the analysis pipeline below. Rows 2-5: Sequential single-plane images from the Z stack image enlarged to demonstrate the image analysis pipeline of plasma membrane order quantification on 2 single cells *in vivo* (rows 2 and 4) and images from individual wavelength from a single-plane (Z=2 for qNSC #1 and Z=3 for qNSC #2, rows 3 and 5) of the Z stack. Scale bar represents 10μm. **(E)** Plasma membrane lipid order assessed by Laurdan ratiometric quantification of different subcellular regions in qNSCs *in vivo* from n=5 young (3-5 months) and n=5 old (20-22 months) mice. Left: representative Laurdan staining on *in vivo* qNSCs from SVZ. Image taken from rigid wavelength (more ordered, green) is overlaid with GFAP (NSC and astrocyte marker, blue). Plasma membrane segment around the cell body as well as around the basal process is outlined in yellow. Right: Membrane order quantified by generalized polarization ratio (GP ratio) from Laurdan staining. SuperPlots showing data from individual cells (cyan/purple dots), as well as the mean of each mouse (gray dots) +/- s.e.m.. Results are from one independent experiment, n=5 mice. *P*-values from Wilcoxon rank-sum test. Results from a second independent experiment are in **Table S14**.

Figure S11

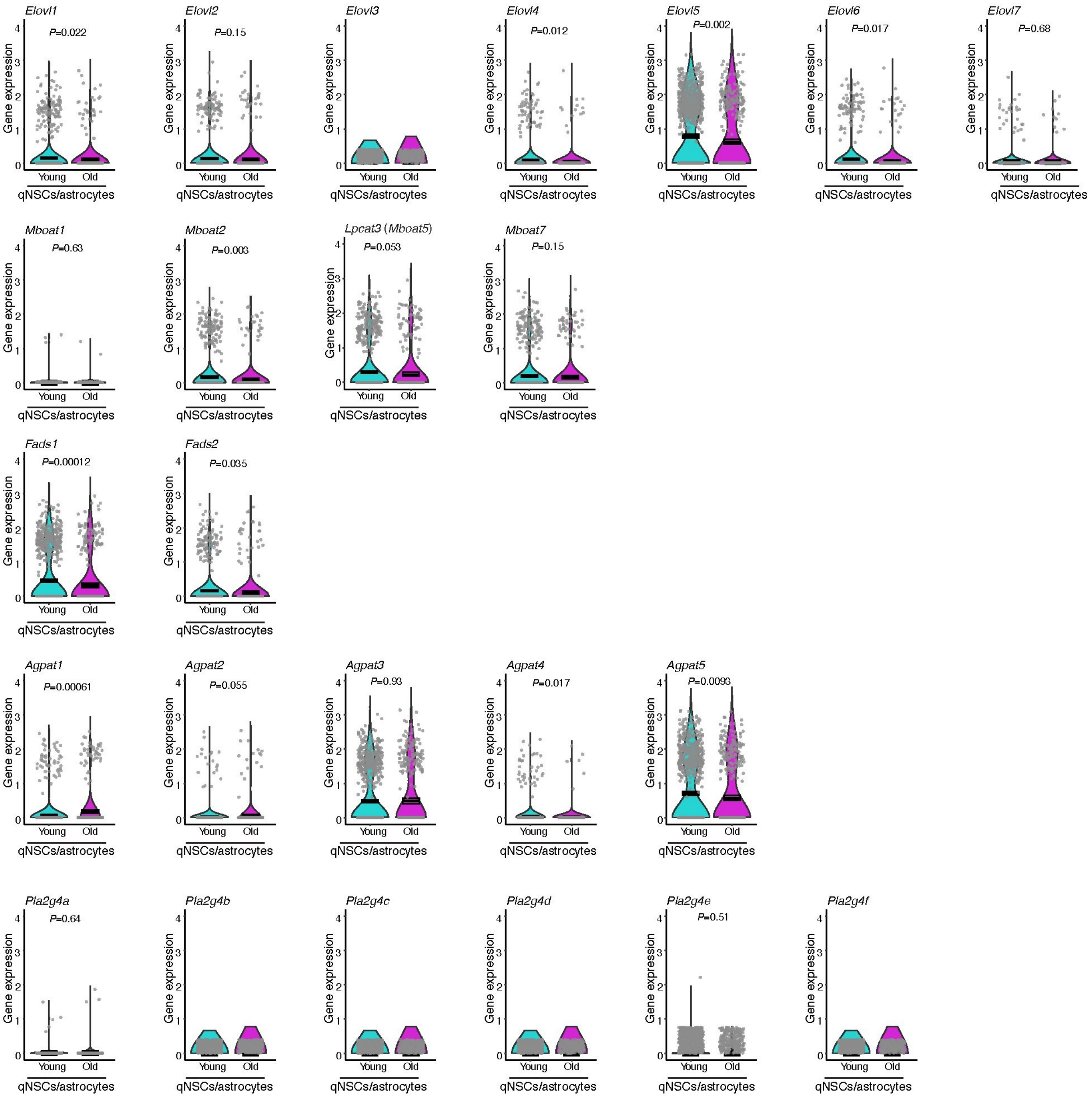
Elovl1
P=0.022
Elovl2
P=0.15
Elovl3
Elovl4
P=0.012
Elovl5
P=0.002
Elovl6
P=0.017
Elovl7
P=0.68
Mboat1
P=0.63
Mboat2
P=0.003
Lpcat3 (Mboat5)
P=0.053
Mboat7
P=0.15
Fads1
P=0.00012
Fads2
P=0.035
Agpat1
P=0.00061
Agpat2
P=0.055
Agpat3
P=0.93
Agpat4
P=0.017
Agpat5
P=0.0093
Pla2g4a
P=0.64
Pla2g4b
Pla2g4c
Pla2g4d
Pla2g4e
P=0.51
Pla2g4f
Gene expression
Young
Old
qNSCs/astrocytes

Fig. S11. ***In vivo*** **expression level of selected genes encoding lipid modification enzymes and other genes in the same enzyme family.**

Violin plots of gene expression in young and old qNSCs/astrocytes from single-cell RNA-seq datasets (see Methods for details). Five selected genes encoding lipid modification enzymes (*Elovl5*, *Mboat2*, *Fads2*, *Agpat3*, *Pla2g4e*), and other genes in the same enzyme family (*Elovl1*, *Elovl2*, *Elovl3*, *Elovl4*, *Elovl6*, *Elovl7*, *Mboat1*, *Lpcat3 (Mboat5)*, *Mboat7*, *Fads1, Agpat1*, *Agpat2*, *Agpat4*, *Agpat5*, *Pla2g4a*, *Pla2g4b*, *Pla2g4c*, *Pla2g4d*, *Pla2g4f*) are included. Boxplot represents mean expression level +/- s.e.m.. Each individual dot represents the normalized gene expression of specified gene in a single cell. Gray bar at the bottom represents cells with gene expression level below detection limit. *P*-values from Wilcoxon rank-sum test. No *P*-value is indicated if expression was not detected in any cells from young or old samples.

Figure S12

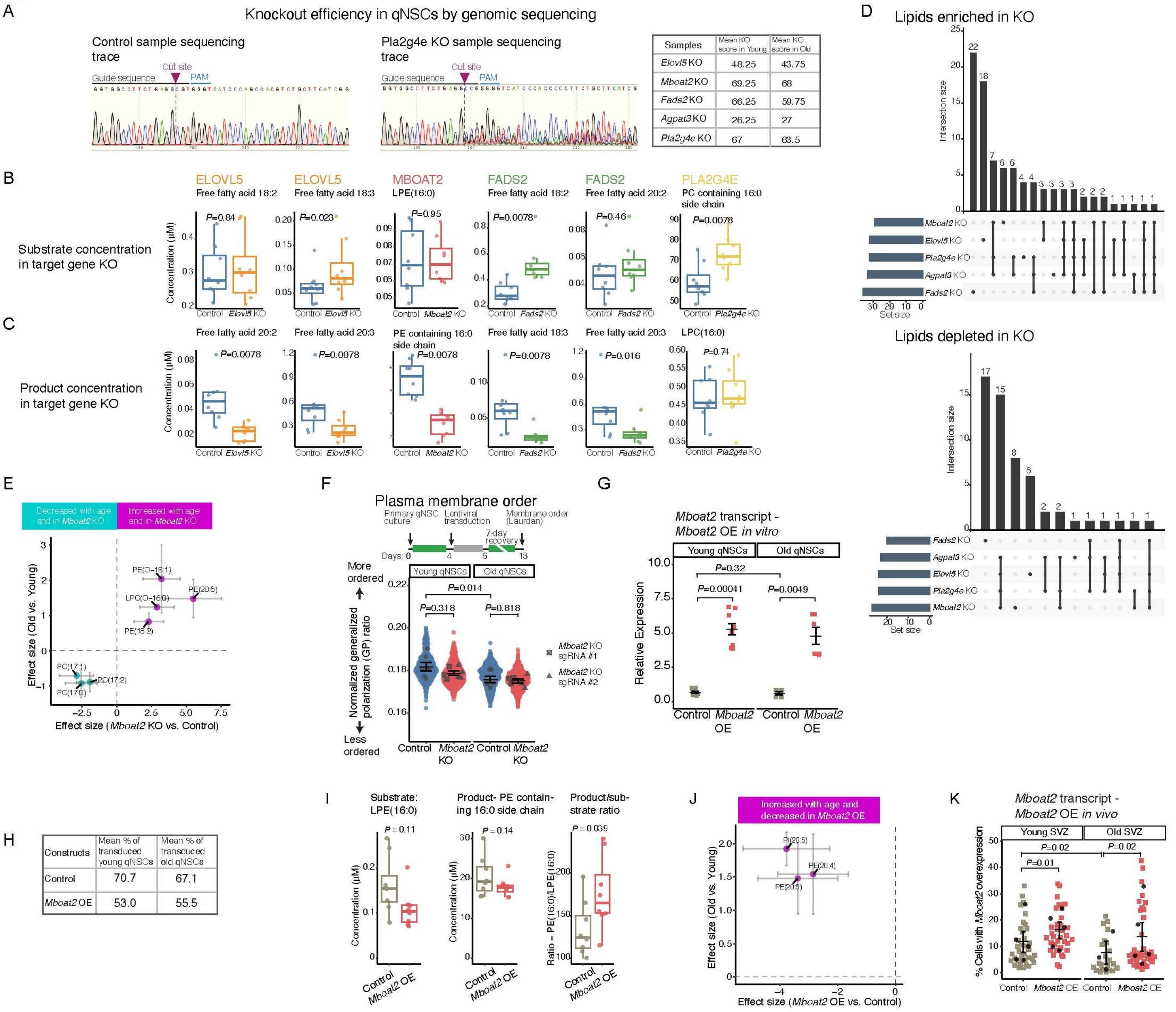

Samples	Mean KO score in Young	Mean KO score in Old
Elovl5 KO	48.25	43.75
Mboat2 KO	69.25	68
Fads2 KO	66.25	59.75
Agpat3 KO	26.25	27
Pla2g4e KO	67	63.5

Constructs	Mean % of transduced young qNSCs	Mean % of transduced old qNSCs
Control	70.7	67.1
Mboat2 OE	53.0	55.5

Fig. S12. Validation of *in vitro* genetic manipulations and their effects on the global lipidome of neural stem cells.

(A) Validation of knockout efficiency by genomic DNA sequencing. Representative sequencing traces from control and knockout (KO) samples of the *Pla2g4e* gene with CRISPR editing are shown (left). Mean KO scores in young and old qNSCs are obtained from Inference of CRISPR Edits (ICE) analysis (see Methods for details) (right). **(B to C)** Substrate (**B**) and product (**C**) level in control and knockout (KO) for each target genes in qNSCs. Data are obtained from untargeted lipidomic analysis. Each dot represents a primary culture from an individual young (3-5 months old) or old (20-22 months old) male mouse. Results are from 1 independent experiment, n=4 primary cultures. *P*-values from Wilcoxon rank-sum test. Substrates are: *Elovl5* KO: free fatty acid 18:2 and 18:3, *Mboat2* KO: LPE(16:0), *Fads2* KO: free fatty acid 18:2 and 20:2 and *Pla2g4e* KO: PC containing 16:0 side chain. Products are: *Elovl5* KO: free fatty acid 20:2 and 20:3, *Mboat2* KO: PE containing 16:0 side chain, *Fads2* KO: free fatty acid 18:3 and 20:3 and *Pla2g4e* KO: LPC(16:0). **(D)** Effects on global lipidome of individual gene knockouts. Sets of overlapping lipids and the number of lipids per set in individual knockouts are shown using UpSet plots. Lipids are divided into ones that are enriched in knockout (top) or depleted in knockout (bottom) when compared to control. **(E)** Side chain composition change with *Mboat2* knockout in old qNSCs. Significant side chain features that change with *Mboat2* knockout by effect size are plotted. X axis indicates the effect size of side chain features (individual dots) between *Mboat2* KO vs. control in old qNSCs. Y axis indicates the effect size between young and old qNSCs (from **Fig. 1E**). For each side chain feature, mean effect size is shown together with s.e.m. (error bar). Dashed lines indicate effect size of 0. **(F)** Plasma membrane order as assessed by Laurdan *in vitro* on young and old qNSCs with *Mboat2* knockout. Primary qNSC cultures from n=6-7 young (3-5 months) and n=6-7 old (20-22 months) mice were transduced with control lentivirus (sgRNAs targeting an unannotated region of the genome) or with sgRNAs targeting *Mboat2* (2 independent sgRNAs) for 2 consecutive days. Plasma membrane order was assessed at the end of a 7-day recovery period following genetic KO. Plasma membrane order was quantified by generalized polarization ratio (GP ratio) from Laurdan staining on young and old control (blue) or *Mboat2* (red) KO qNSCs. SuperPlots showing data from individual cells (colored dots), as well as the mean of each primary culture (gray dots) +/- s.e.m.. Results from 3 independent experiments, n=6-7 primary cultures. *P*-values from Wilcoxon rank-sum test on quantification of individual primary cultures. **(G)** Validation of *Mboat2* overexpression *in vitro* by RT-qPCR. Relative expression level of *Mboat2* by RT-qPCR is shown for young and old qNSCs with control (grey) or *Mboat2* (red) overexpression. Dot and whisker plot from 2 independent experiments, n=6-9 primary cultures. Horizontal bar: mean. Whisker: +/- s.e.m.. Each dot represents a primary culture from an individual mouse. *P*-values from Wilcoxon rank-sum test. **(H)** Lentiviral transduction efficiency of *Mboat2* overexpression *in vitro*. Mean percentage of transduced cells based on co-expressed reporters in young and old qNSCs are presented. **(I)** Validation of *Mboat2* overexpression *in vitro* by the substrate and product level from lipidomic analysis. Lipid concentration of LPE(16:0) (substrate of MBOAT2, left panel), PE containing 16:0 side chain (product of MBOAT2, middle panel), and the ratio between PE containing 16:0 side chain and LPE(16:0) (product to substrate ratio, right panel) are shown. Box and whisker plot from 1 independent experiment, n=4 primary qNSC cultures. Horizontal bar: median. Whisker: Distance between the first/third quartile to the highest/lowest value, respectively, or 1.5 times the distance between the first/third quartile, whichever is smaller. Each dot represents a primary culture from an individual mouse. *P*-values from Wilcoxon rank-sum test. **(J)** Side chain composition change with *Mboat2* overexpression in old qNSCs. Significant side chain features that change with *Mboat2* overexpression by effect size are plotted. X axis indicates the

effect size of side chain features (individual dots) between *Mboat2* OE vs. control in old qNSCs. Y axis indicates the effect size between young and old qNSCs (from **Fig. 1E**). For each side chain feature, mean effect size is shown together with s.e.m. (error bar). Dashed lines indicate effect size of 0. **(K)** Validation of *Mboat2* overexpression *in vivo* by *in situ* mRNA hybridization. Percentage of all SVZ cells containing positive staining of *Mboat2* transcripts is quantified in young and old mice transduced with control lentivirus (grey) or lentivirus overexpressing *Mboat2* (red). SuperPlots showing data from individual tiled images (colored legend), as well as the mean of each individual animal (gray dots) +/- s.e.m.. n=3-5 animals. *P*-values from Wilcoxon rank-sum test on quantification of individual animals.

Figure S13

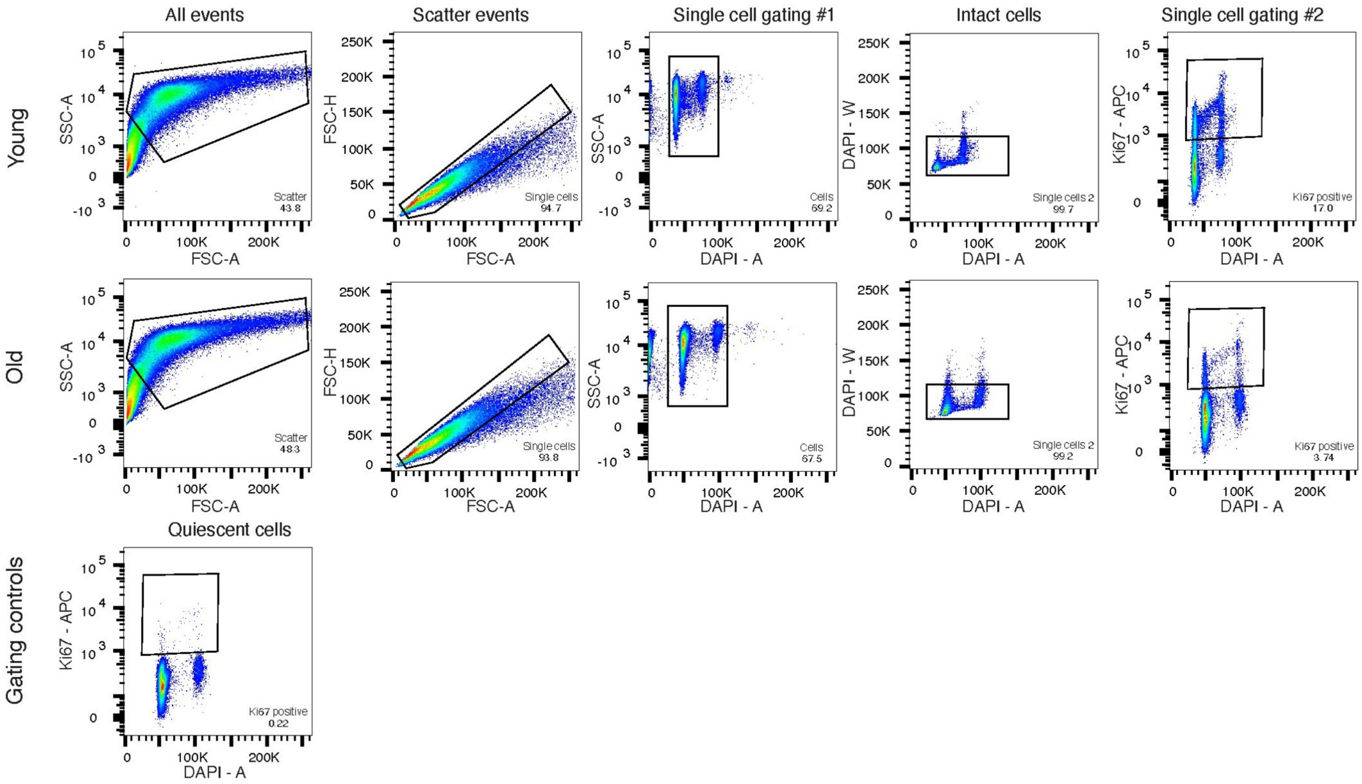
All events
Scatter events
Single cell gating #1
Intact cells
Single cell gating #2
Young
Old
Gating controls
Quiescent cells
SSC-A
FSC-A
FSC-H
DAPI - A
DAPI - W
Ki67 - APC
Scatter 43.8
Single cells 94.7
Cells 69.2
Single cells 2 99.7
Ki67 positive 17.0
Scatter 48.3
Single cells 93.8
Cells 67.5
Single cells 2 99.2
Ki67 positive 3.74
Ki67 positive 0.22

Fig. S13. FACS gating to assess qNSC activation *in vitro*.
FACS gating strategy to assess the ability of young and old quiescent NSCs to activate *in vitro*. Quiescent (non-proliferating) NSCs were used as negative control for the proliferation marker Ki67. The cell population on which each respective FACS gating was done is indicated above each plot.

Figure S14

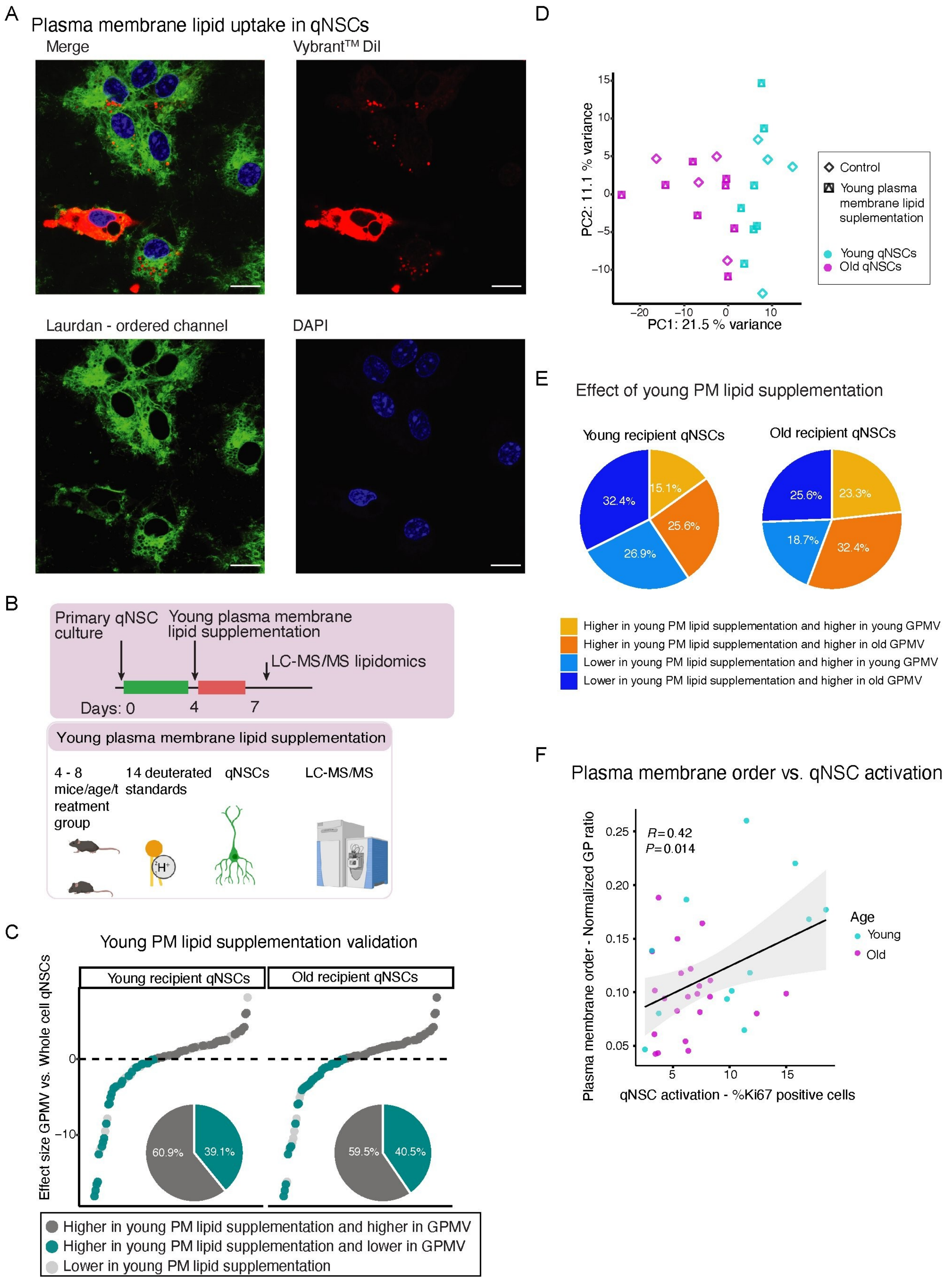
A
Plasma membrane lipid uptake in qNSCs
Merge
VybrantTM DiI
Laurdan - ordered channel
DAPI
B
Primary qNSC culture
Young plasma membrane lipid supplementation
LC-MS/MS lipidomics
Days: 0
4
7
Young plasma membrane lipid supplementation
4 - 8 mice/age/treatment group
14 deuterated standards
qNSCs
LC-MS/MS
C
Young PM lipid supplementation validation
Young recipient qNSCs
Old recipient qNSCs
Effect size GPMV vs. Whole cell qNSCs
60.9%
39.1%
59.5%
40.5%
Higher in young PM lipid supplementation and higher in GPMV
Higher in young PM lipid supplementation and lower in GPMV
Lower in young PM lipid supplementation
D
PC2: 11.1 % variance
PC1: 21.5 % variance
Control
Young plasma membrane lipid suplementation
Young qNSCs
Old qNSCs
E
Effect of young PM lipid supplementation
Young recipient qNSCs
Old recipient qNSCs
15.1%
25.6%
26.9%
32.4%
23.3%
32.4%
18.7%
25.6%
Higher in young PM lipid supplementation and higher in young GPMV
Higher in young PM lipid supplementation and higher in old GPMV
Lower in young PM lipid supplementation and higher in young GPMV
Lower in young PM lipid supplementation and higher in old GPMV
F
Plasma membrane order vs. qNSC activation
R=0.42
P=0.014
Plasma membrane order - Normalized GP ratio
qNSC activation - %Ki67 positive cells
Age
Young
Old

Fig. S14. Supplementation of plasma membrane lipids from young cells boosts the activation ability of old quiescent neural stem cells.

(A) Representative confocal fluorescence images of primary qNSC cultures after plasma membrane lipid supplementation for 72 hours. Cells were imaged with Laurdan rigid wavelength (green), lipophilic dye Vybrant^{TM} DiI for labelling lipid extract (red), and DAPI (blue). Scale bar: 10μm. Labelled supplemented lipids were found in puncta, indicating successful uptake by the cell through the endocytic system. Endocytosed lipids can then be used by different compartments, including plasma membranes. Excess intracellular Vybrant^{TM} DiI can diffuse laterally and stain the entire cell. **(B)** Experimental set up for lipidomics on plasma membrane lipid supplementation of recipient qNSCs. Primary qNSC cultures (recipient cells) from n=4-7 young (3-5 months) and n=4-7 old (20-22 months) mice were induced into quiescence for 4 days and then supplemented with young plasma membrane lipids for 3 consecutive days. Cells were then harvested for untargeted lipidomic analysis by LC-MS/MS. Number of animals used, number of internal deuterated lipidomic standards used and lipidomic platform (LC-MS/MS) are indicated. **(C)** Validation of plasma membrane lipid supplementation by lipidomic analysis. Individual lipids (individual dots) are ranked based on the effect size calculated between GPMV lipidomic dataset (**Fig. 4**) and whole cell lipidomic dataset in young and old qNSCs (*In vitro* #1, **Fig. 1**). Lipids are color-coded based on whether they have a lower abundance (light grey) or higher abundance in cells supplemented with young plasma membrane lipids. The latter group is further labelled based on whether they are more abundant (dark grey) or less abundant (dark green) in GPMV than whole cell extract. Pie chart summarizes the percentage of 2 categories of lipids that has a higher abundance in cells supplemented with young plasma membrane lipids. **(D)** Principal component analysis (PCA) on log2 transformed concentration of all lipids from young (cyan) and old (purple) cultures with (square with triangle inside) or without (diamond) young plasma membrane lipid supplementation. n=4-7 primary cultures. **(E)** Effect of plasma membrane lipid supplementation by lipid category. Pie chart summarizing the effects on lipids following young plasma membrane (PM) lipid supplementation. Percentage of lipids in the following 4 categories are shown: Higher in young PM lipid supplementation and higher in young GPMV, Higher in young PM lipid supplementation and higher in old GPMV, Lower in young PM lipid supplementation and higher in young GPMV, Lower in young PM lipid supplementation and higher in old GPMV. **(F)** Correlation between plasma membrane order and activation in young and old qNSCs upon plasma membrane lipid supplementation. Pearson correlation was performed on generalized polarization ratio (GP ratio) from Laurdan staining, and percentage of qNSCs that activate (% Ki67 positive) from each individual young qNSC culture (cyan dots) or old qNSC cultures (purple dots) with plasma membrane lipid supplementations. *R* and *P*-values from Pearson correlation are shown. Linear regression line (black line) with 95% confidence interval (gray shade) is shown.

Created by

Contents of Report https://lipidomicstandards.org, version v2.5.0

Separation Workflow 2
- Overall study design 2
- Lipid extraction 2
- Analytical platform 2
- Quality control 2
- Method qualification and validation 2
- Reporting 3

Sample Descriptions 3
- In vitro / Mouse / Cells 3
- In vitro Experiment #2 / Mouse / Cells 3
- In vivo isolated qNSCs / Mouse / Cells 3
- GPMV / Mouse / Cells 3
- "Mboat2 overexpression" and "Plasma membrane lipid supplementation" / Mouse / Cells 4

Lipid Class Descriptions 4
- 1) DG[M+NH4]+ / Lipid identification 4
- 1) DG[M+NH4]+ / Lipid quantification 4
- 2) TG[M+NH4]+ / Lipid identification 4
- 2) TG[M+NH4]+ / Lipid quantification 5
- 3) Cer[M+H-H2O]+ / Lipid identification 5
- 3) Cer[M+H-H2O]+ / Lipid quantification 6
- 4) Cer[M+HCOO]- / Lipid identification 6
- 4) Cer[M+HCOO]- / Lipid quantification 6
- 5) PC[M+H]+ / Lipid identification 6
- 5) PC[M+H]+ / Lipid quantification 7
- 6) PC[M+HCOO]- / Lipid identification 7
- 6) PC[M+HCOO]- / Lipid quantification 7
- 7) PC O[M+H]+ / Lipid identification 7
- 7) PC O[M+H]+ / Lipid quantification 8
- 8) PC O[M+HCOO]- / Lipid identification 8
- 8) PC O[M+HCOO]- / Lipid quantification 8
- 9) PE[M+H]+ / Lipid identification 9
- 9) PE[M+H]+ / Lipid quantification 9
- 10) PE[M-H]- / Lipid identification 9
- 10) PE[M-H]- / Lipid quantification 10
- 11) PE O[M+H]+ / Lipid identification 10
- 11) PE O[M+H]+ / Lipid quantification 10
- 12) PE O[M-H]- / Lipid identification 11
- 12) PE O[M-H]- / Lipid quantification 11
- 13) PE P[M+H]+ / Lipid identification 11
- 13) PE P[M+H]+ / Lipid quantification 11
- 14) LPC[M+H]+ / Lipid identification 12
- 14) LPC[M+H]+ / Lipid quantification 12
- 15) LPC[M+HCOO]- / Lipid identification 12
- 15) LPC[M+HCOO]- / Lipid quantification 13
- 16) EtherLPC-O (EtherLPC-P)[M+H]+ / Lipid identification 13
- 16) EtherLPC-O (EtherLPC-P)[M+H]+ / Lipid quantification 13
- 17) SM[M+H]+ / Lipid identification 14
- 17) SM[M+H]+ / Lipid quantification 14
- 18) SM[M+HCOO]- / Lipid identification 14
- 18) SM[M+HCOO]- / Lipid quantification 15
- 19) LPE[M+H]+ / Lipid identification 15
- 19) LPE[M+H]+ / Lipid quantification 15
- 20) LPE[M-H]- / Lipid identification 15
- 20) LPE[M-H]- / Lipid quantification 16
- 21) EtherLPE-O (EtherLPE-P)[M-H]- / Lipid identification 16
- 21) EtherLPE-O (EtherLPE-P)[M-H]- / Lipid quantification 16
- 22) PG[M-H]- / Lipid identification 16
- 22) PG[M-H]- / Lipid quantification 17
- 23) PI[M-H]- / Lipid identification 17
- 23) PI[M-H]- / Lipid quantification 18
- 24) PS[M-H]- / Lipid identification 18
- 24) PS[M-H]- / Lipid quantification 18
- 25) PG[M+NH4]+ / Lipid identification 19
- 25) PG[M+NH4]+ / Lipid quantification 19
- 26) PI[M+NH4]+ / Lipid identification 19
- 26) PI[M+NH4]+ / Lipid quantification 19

27) PS[M+H]+ / Lipid identification . . . 20
27) PS[M+H]+ / Lipid quantification . . . 20

Separation Workflow

Overall study design

Title of the study	Lipidomic profiling reveals age-dependent changes in complex plasma membrane lipids that regulate neural stem cell aging		
Document creation date	03/26/2026	Principal investigator	Xiaoai Zhao
Institution	Yale University	Corresponding Email	xiaoai.zhao@yale.edu
Is the workflow targeted or untargeted?	Untargeted	Clinical	No

Lipid extraction

Extraction method	2-phase system	pH adjustment	None
2-phase system	Folch	Special conditions	Sonication
Were internal standards added prior extraction?	Yes		

Analytical platform

Ionizaton additives	Ammonium acetate, Formic acid	Number of separation dimensions	One dimension
Separation type 1	LC	Separation mode 1 (liquid)	RP
Detector	Mass spectrometer	MS type	Orbitrap
MS vendor	Thermo	Ion source	ESI
MS Level	MS^1, MS^2	Mass resolution for detected ion at MS^1	High resolution
Resolution at m/z 200 at MS^1	70000	Mass accuracy in ppm at MS^1	10
Recording mode of raw data at MS^1	Centroid mode	Mass window for precursor ion isolation (in Da total isolation window)	1
Mass resolution for detected ion at MS^2	High resolution	Resolution at m/z 200 at MS^2	35000
Mass accuracy in ppm at MS^2	10	Recording mode of raw data at MS^2	Profile mode
Was/Were additional dimension/techniques used	No		

Quality control

Blanks	Yes	Type of Blanks	Extraction blank
Quality control	Yes	Type of QC sample	Sample pool

Method qualification and validation

Method validation	Yes	Lipid recovery	Yes
Dynamic quantification range	Yes	Limit of quantitation (LOQ)/Limit of detection (LOD)	Yes
Precision	Yes	Accuracy	Yes
Guidelines followed	None		

Reporting

Are reported raw data uploaded into repository?	Yes	Link to repository / ID to entry	Raw lipidomics data were uploaded to Metabolomicsworkbench.org under StudyID: ST002258, ST002259, ST002260, ST004116 and ST004117.
Are metadata available?	Yes	Summary data	Quantification and identification data
Raw data upload	Yes		

Sample Descriptions

In vitro / Mouse / Cells

Storage and collection conditions	Available	Provided preanalytical information	Time to freeze, Storage time (month), Freeze-thaw cycles, Preservation method
Temperature handling original sample	N2	Instant sample preparation	Yes
Time to freeze	between 5 and 10 min	Snap freezing in liquid N2	Yes
Storage temperature	-80 °C	Storage time (month)	0.5
Freeze-thaw cycles	1	Additives	None

In vitro Experiment #2 / Mouse / Cells

Storage and collection conditions	Available	Provided preanalytical information	Time to freeze, Storage time (month), Freeze-thaw cycles, Preservation method
Temperature handling original sample	N2	Instant sample preparation	No
Time to freeze	between 5 and 10 minutes	Snap freezing in liquid N2	Yes
Storage temperature	-80 °C	Storage time (month)	0.5
Freeze-thaw cycles	1	Additives	None

In vivo isolated qNSCs / Mouse / Cells

Storage and collection conditions	Available	Provided preanalytical information	Time to freeze, Storage time (month), Freeze-thaw cycles, Preservation method
Temperature handling original sample	N2	Instant sample preparation	No
Time to freeze	between 5 and 10 minutes	Snap freezing in liquid N2	Yes
Storage temperature	-80 °C	Storage time (month)	0.5
Freeze-thaw cycles	1	Additives	None

GPMV / Mouse / Cells

Storage and collection conditions	Available	Provided preanalytical information	Time to freeze, Storage time (month), Freeze-thaw cycles, Preservation method
Temperature handling original sample	N2	Instant sample preparation	No
Time to freeze	between 60 and 70 minutes	Snap freezing in liquid N2	Yes
Storage temperature	-80 °C	Storage time (month)	0.5
Freeze-thaw cycles	1	Additives	None

“Mboat2 overexpression” and “Plasma membrane lipid supplementation” / Mouse / Cells

Storage and collection conditions	Available	Provided preanalytical information	Time to freeze, Storage time (month), Freeze-thaw cycles, Preservation method
Temperature handling original sample	N2	Instant sample preparation	No
Time to freeze	between 5 and 10 minutes	Snap freezing in liquid N2	Yes
Storage temperature	-80 °C	Storage time (month)	0.5
Freeze-thaw cycles	1	Additives	None

Lipid Class Descriptions

1) DG[M+NH4]+ / Lipid identification

Lipid class	DG	MS Level for identification	MS^2
Identification level	Molecular species level	MS^2 adduct	[M+NH4]+
Fragments for identification			

Fragment name
NL of H2O
DMAG Side chain 1
DMAG Side chain 2

Isotope correction at MS^2	No	MS^2 verified by standard	Yes
Background check at MS^2	No	Did you presume assumptions for identification?	No
Check on:	Isomeric overlap, Isobaric overlap	Limit of detection	Signal threshold
RT verified by standard	Yes	Separation of isobaric/isomeric interferece confirmed	No
Model for separation prediction	No	Lipid Identification Software	MS-DIAL
Data manipulation	Smoothing, Centroiding, Manual integration	Nomenclature for intact lipid molecule	Yes
Nomenclature for fragment ions	Yes		

1) DG[M+NH4]+ / Lipid quantification

Quantitative	Yes	MS Level for quantification	MS^1
Internal lipid standard(s) MS^1			

Internal standard	Endogenous subclass
DG 15:0_18:1(d7)	DG

Type of quantification	Internal standard amount	Response correction	No
Type I isotope correction	No	Limit of quantification	Signal threshold
Normalization to reference	Yes	Lipid Quantification Software	MS-DIAL
Batch correction	No		

2) TG[M+NH4]+ / Lipid identification

Lipid class	TG	MS Level for identification	MS^2

Identification level	Molecular species level	MS^2 adduct	[M+NH4]+

Fragments for identification

Fragment name
NL of Acyl Side chain 1 + H2O
NL of Acyl Side chain 2 + H2O
NL of Acyl Side chain 3 + H2O

Isotope correction at MS^2	No	MS^2 verified by standard	Yes
Background check at MS^2	No	Did you presume assumptions for identification?	No
Check on:	Isomeric overlap, Isobaric overlap	Limit of detection	Signal threshold
RT verified by standard	Yes	Separation of isobaric/isomeric interferece confirmed	No
Model for separation prediction	No	Lipid Identification Software	MS-DIAL
Data manipulation	Smoothing, Centroiding, Manual integration	Nomenclature for intact lipid molecule	Yes
Nomenclature for fragment ions	Yes		

2) TG[M+NH4]+ / Lipid quantification

Quantitative	Yes	MS Level for quantification	MS^1

Internal lipid standard(s) MS^1

Internal standard	Endogenous subclass
TG 15:0_18:1(d7)_15:0	TG

Type of quantification	Internal standard amount	Response correction	No
Type I isotope correction	No	Limit of quantification	Signal threshold
Normalization to reference	Yes	Lipid Quantification Software	MS-DIAL
Batch correction	No		

3) Cer[M+H-H2O]+ / Lipid identification

Lipid class	Cer	MS Level for identification	MS^2
Identification level	Molecular species level	MS^2 adduct	[M+H-H2O]+

Fragments for identification

Fragment name
NL of H2O
SPB Side chain 1 - H2O

Isotope correction at MS^2	No	MS^2 verified by standard	Yes
Background check at MS^2	No	Did you presume assumptions for identification?	No
Check on:	Isomeric overlap, Isobaric overlap	Limit of detection	Signal threshold
RT verified by standard	Yes	Separation of isobaric/isomeric interferece confirmed	No
Model for separation prediction	No	Lipid Identification Software	MS-DIAL
Data manipulation	Smoothing, Centroiding, Manual Integration	Nomenclature for intact lipid molecule	Yes
Nomenclature for fragment ions	Yes		

3) Cer[M+H-H2O]+ / Lipid quantification

Quantitative	Yes	MS Level for quantification	MS^1

Internal lipid standard(s) MS^1

Internal standard	Endogenous subclass
Cer 18:1(d7);2O/15:0	Cer_NS

Type of quantification	Internal standard amount	Response correction	No
Type I isotope correction	No	Limit of quantification	Signal threshold
Normalization to reference	Yes	Lipid Quantification Software	MS-DIAL
Batch correction	No		

4) Cer[M+HCOO]- / Lipid identification

Lipid class	Cer	MS Level for identification	MS^2
Identification level	Molecular species level	MS^2 adduct	[M+HCOO]-

Fragments for identification

Fragment name
Acyl Side chain 1 + C2H3N

Isotope correction at MS^2	No	MS^2 verified by standard	Yes
Background check at MS^2	No	Did you presume assumptions for identification?	No
Check on:	Isomeric overlap, Isobaric overlap	Limit of detection	Signal threshold
RT verified by standard	Yes	Separation of isobaric/isomeric interferece confirmed	No
Model for separation prediction	No	Lipid Identification Software	MS-DIAL
Data manipulation	Smoothing, Centroiding, Manual Integration	Nomenclature for intact lipid molecule	Yes
Nomenclature for fragment ions	Yes		

4) Cer[M+HCOO]- / Lipid quantification

Quantitative	No	Normalization to reference	No
Batch correction	No		

5) PC[M+H]+ / Lipid identification

Lipid class	PC	MS Level for identification	MS^2
Identification level	Molecular species level	MS^2 adduct	[M+H]+

Fragments for identification

Fragment name
C5H15NO4P+ Phosphorylcholine

Isotope correction at MS^2	No	MS^2 verified by standard	Yes
Background check at MS^2	No	Did you presume assumptions for identification?	No
Check on:	Isomeric overlap, Isobaric overlap	Limit of detection	Signal threshold

RT verified by standard	No	Separation of isobaric/isomeric interferece confirmed	No
Model for separation prediction	No	Lipid Identification Software	MS-DIAL
Data manipulation	Smoothing, Centroiding, Manual Integration	Nomenclature for intact lipid molecule	Yes
Nomenclature for fragment ions	Yes		

5) PC[M+H]+ / Lipid quantification

Quantitative	Yes	MS Level for quantification	MS^1

Internal lipid standard(s) MS^1

Internal standard	Endogenous subclass
PC 15:0_18:1(d7)	PC

Type of quantification	Internal standard amount	Response correction	No
Type I isotope correction	No	Limit of quantification	Signal threshold
Normalization to reference	Yes	Lipid Quantification Software	MS-DIAL
Batch correction	No		

6) PC[M+HCOO]- / Lipid identification

Lipid class	PC	MS Level for identification	MS^2
Identification level	Molecular species level	MS^2 adduct	[M+HCOO]-

Fragments for identification

Fragment name
FA Side chain 1
FA Side chain 2

Isotope correction at MS^2	No	MS^2 verified by standard	Yes
Background check at MS^2	No	Did you presume assumptions for identification?	No
Check on:	Isomeric overlap, Isobaric overlap	Limit of detection	Signal threshold
RT verified by standard	Yes	Separation of isobaric/isomeric interferece confirmed	No
Model for separation prediction	No	Lipid Identification Software	MS-DIAL
Data manipulation	Smoothing, Centroiding, Manual Integration	Nomenclature for intact lipid molecule	Yes
Nomenclature for fragment ions	Yes		

6) PC[M+HCOO]- / Lipid quantification

Quantitative	No	Normalization to reference	No
Batch correction	No		

7) PC O[M+H]+ / Lipid identification

Lipid class	PC O	MS Level for identification	MS^2
Identification level	Molecular species level	MS^2 adduct	[M+H]+

Fragments for identification

Fragment name
C5H15NO4P+ Phosphorylcholine

Isotope correction at MS^2	No	MS^2 verified by standard	Yes
Background check at MS^2	No	Did you presume assumptions for identification?	No
Check on:	Isomeric overlap, Isobaric overlap	Limit of detection	Signal threshold
RT verified by standard	Yes	Separation of isobaric/isomeric interferece confirmed	No
Model for separation prediction	No	Lipid Identification Software	MS-DIAL
Data manipulation	Smoothing, Centroiding, Manual Integration	Nomenclature for intact lipid molecule	Yes
Nomenclature for fragment ions	Yes		

7) PC O[M+H]+ / Lipid quantification

Quantitative	Yes	MS Level for quantification	MS^1

Internal lipid standard(s) MS^1

Internal standard	Endogenous subclass
PC 15:0_18:1(d7)	EtherPC-O

Type of quantification	Internal standard amount	Response correction	No
Type I isotope correction	No	Limit of quantification	Signal threshold
Normalization to reference	Yes	Lipid Quantification Software	MS-DIAL
Batch correction	No		

8) PC O[M+HCOO]- / Lipid identification

Lipid class	PC O	MS Level for identification	MS^2
Identification level	Molecular species level	MS^2 adduct	[M+HCOO]-

Fragments for identification

Fragment name
LPC Ether-O Side chain 1 - CH3

Isotope correction at MS^2	No	MS^2 verified by standard	No
Background check at MS^2	No	Did you presume assumptions for identification?	No
Check on:	Isomeric overlap, Isobaric overlap	Limit of detection	Signal threshold
RT verified by standard	No	Separation of isobaric/isomeric interferece confirmed	No
Model for separation prediction	No	Lipid Identification Software	MS-DIAL
Data manipulation	Smoothing, Centroiding, Manual integration	Nomenclature for intact lipid molecule	Yes
Nomenclature for fragment ions	Yes		

8) PC O[M+HCOO]- / Lipid quantification

Quantitative	No	Normalization to reference	No
Batch correction	No		

9) PE[M+H]+ / Lipid identification

Lipid class	PE	MS Level for identification	MS^2
Identification level	Molecular species level	MS^2 adduct	[M+H]+

Fragments for identification

Fragment name
NL of C2H8NO4P Ethanolaminephosphate
Acyl Side chain 1
Acyl Side chain 2

Isotope correction at MS^2	No	MS^2 verified by standard	Yes
Background check at MS^2	No	Did you presume assumptions for identification?	No
Check on:	Isomeric overlap, Isobaric overlap	Limit of detection	Signal threshold
RT verified by standard	Yes	Separation of isobaric/isomeric interferece confirmed	No
Model for separation prediction	No	Lipid Identification Software	MS-DIAL
Data manipulation	Smoothing, Centroiding, Manual Integration	Nomenclature for intact lipid molecule	Yes
Nomenclature for fragment ions	Yes		

9) PE[M+H]+ / Lipid quantification

Quantitative	Yes	MS Level for quantification	MS^1

Internal lipid standard(s) MS^1

Internal standard	Endogenous subclass
PE 15:0_18:1(d7)	PE

Type of quantification	Internal standard amount	Response correction	No
Type I isotope correction	No	Limit of quantification	Signal threshold
Normalization to reference	Yes	Lipid Quantification Software	MS-DIAL
Batch correction	No		

10) PE[M-H]- / Lipid identification

Lipid class	PE	MS Level for identification	MS^2
Identification level	Molecular species level	MS^2 adduct	[M-H]-

Fragments for identification

Fragment name
C5H11NO5P- Characteristic fragment
FA Side chain 1
FA Side chain 2

Isotope correction at MS^2	No	MS^2 verified by standard	Yes

Background check at MS^2	No	Did you presume assumptions for identification?	No
Check on:	Isomeric overlap, Isobaric overlap	Limit of detection	Signal threshold
RT verified by standard	Yes	Separation of isobaric/isomeric interferece confirmed	No
Model for separation prediction	No	Lipid Identification Software	MS-DIAL
Data manipulation	Smoothing, Centroiding, Manual integration	Nomenclature for intact lipid molecule	Yes
Nomenclature for fragment ions	Yes		

10) PE[M-H]- / Lipid quantification

Quantitative	No	Normalization to reference	No
Batch correction	No		

11) PE O[M+H]+ / Lipid identification

Lipid class	PE O	MS Level for identification	MS^2
Identification level	Molecular species level	MS^2 adduct	[M+H]+

Fragments for identification

Fragment name
NL of C2H8NO4P Ethanolaminephosphate
Acyl Side chain 1

Isotope correction at MS^2	No	MS^2 verified by standard	No
Background check at MS^2	No	Did you presume assumptions for identification?	No
Check on:	Isomeric overlap, Isobaric overlap	Limit of detection	Signal threshold
RT verified by standard	No	Separation of isobaric/isomeric interferece confirmed	No
Model for separation prediction	No	Lipid Identification Software	MS-DIAL
Data manipulation	Smoothing, Centroiding, Manual Integration	Nomenclature for intact lipid molecule	Yes
Nomenclature for fragment ions	Yes		

11) PE O[M+H]+ / Lipid quantification

Quantitative	Yes	MS Level for quantification	MS^1

Internal lipid standard(s) MS^1

Internal standard	Endogenous subclass
PE 15:0_18:1(d7)	EtherPE-O

Type of quantification	Internal standard amount	Response correction	No
Type I isotope correction	No	Limit of quantification	Signal threshold
Normalization to reference	Yes	Lipid Quantification Software	MS-DIAL
Batch correction	No		

12) PE O[M-H]- / Lipid identification

Lipid class	PE O	MS Level for identification	MS^2
Identification level	Molecular species level	MS^2 adduct	[M-H]-

Fragments for identification

Fragment name
FA Side chain 1

Isotope correction at MS^2	No	MS^2 verified by standard	No
Background check at MS^2	No	Did you presume assumptions for identification?	No
Check on:	Isomeric overlap, Isobaric overlap	Limit of detection	Signal threshold
RT verified by standard	No	Separation of isobaric/isomeric interferece confirmed	No
Model for separation prediction	No	Lipid Identification Software	MS-DIAL
Data manipulation	Smoothing, Centroiding, Manual Integration	Nomenclature for intact lipid molecule	Yes
Nomenclature for fragment ions	Yes		

12) PE O[M-H]- / Lipid quantification

Quantitative	No	Normalization to reference	No
Batch correction	No		

13) PE P[M+H]+ / Lipid identification

Lipid class	PE P	MS Level for identification	MS^2
Identification level	Molecular species level	MS^2 adduct	[M+H]+

Fragments for identification

Fragment name
NL of C2H8NO4P Ethanolaminephosphate
Ether-P Side chain 1 + C2H8NO3P
DMAG Side chain 2

Isotope correction at MS^2	No	MS^2 verified by standard	No
Background check at MS^2	No	Did you presume assumptions for identification?	No
Check on:	Isomeric overlap, Isobaric overlap	Limit of detection	Signal threshold
RT verified by standard	No	Separation of isobaric/isomeric interferece confirmed	No
Model for separation prediction	No	Lipid Identification Software	MS-DIAL
Data manipulation	Smoothing, Centroiding, Manual Integration	Nomenclature for intact lipid molecule	Yes
Nomenclature for fragment ions	Yes		

13) PE P[M+H]+ / Lipid quantification

Quantitative	Yes	MS Level for quantification	MS^1

Internal lipid standard(s) MS^1

Internal standard	Endogenous subclass

PE 15:0_18:1(d7)	EtherPE-P

Type of quantification	Internal standard amount	Response correction	No
Type I isotope correction	No	Limit of quantification	Signal threshold
Normalization to reference	Yes	Lipid Quantification Software	MS-DIAL
Batch correction	No		

14) LPC[M+H]+ / Lipid identification

Lipid class	LPC	MS Level for identification	MS^2
Identification level	Molecular species level	MS^2 adduct	[M+H]+

Fragments for identification

Fragment name
C5H15NO4P+ Phosphorylcholine

Isotope correction at MS^2	No	MS^2 verified by standard	Yes
Background check at MS^2	No	Did you presume assumptions for identification?	No
Check on:	Isomeric overlap, Isobaric overlap	Limit of detection	Signal threshold
RT verified by standard	Yes	Separation of isobaric/isomeric interferece confirmed	No
Model for separation prediction	No	Lipid Identification Software	MS-DIAL
Data manipulation	Smoothing, Centroiding, Manual Integration	Nomenclature for intact lipid molecule	Yes
Nomenclature for fragment ions	Yes		

14) LPC[M+H]+ / Lipid quantification

Quantitative	Yes	MS Level for quantification	MS^1

Internal lipid standard(s) MS^1

Internal standard	Endogenous subclass
LPC 18:1(d7)	LPC

Type of quantification	Internal standard amount	Response correction	No
Type I isotope correction	No	Limit of quantification	Signal threshold
Normalization to reference	Yes	Lipid Quantification Software	MS-DIAL
Batch correction	No		

15) LPC[M+HCOO]- / Lipid identification

Lipid class	LPC	MS Level for identification	MS^2
Identification level	Molecular species level	MS^2 adduct	[M+HCOO]-

Fragments for identification

Fragment name
FA Side chain 1

Isotope correction at MS^2	No	MS^2 verified by standard	Yes

Background check at MS^2	No	Did you presume assumptions for identification?	No
Check on:	Isomeric overlap, Isobaric overlap	Limit of detection	Signal threshold
RT verified by standard	Yes	Separation of isobaric/isomeric interferece confirmed	No
Model for separation prediction	No	Lipid Identification Software	MS-DIAL
Data manipulation	Smoothing, Centroiding, Manual Integration	Nomenclature for intact lipid molecule	Yes
Nomenclature for fragment ions	Yes		

15) LPC[M+HCOO]- / Lipid quantification

Quantitative	No	Normalization to reference	No
Batch correction	No		

16) EtherLPC-O (EtherLPC-P)[M+H]+ / Lipid identification

Lipid class	EtherLPC-O (EtherLPC-P)	MS Level for identification	MS^2
Identification level	Molecular species level	MS^2 adduct	[M+H]+

Fragments for identification

Fragment name
C5H15NO4P+ Phosphorylcholine

Isotope correction at MS^2	No	MS^2 verified by standard	No
Background check at MS^2	No	Did you presume assumptions for identification?	No
Check on:	Isomeric overlap, Isobaric overlap	Limit of detection	Signal threshold
RT verified by standard	No	Separation of isobaric/isomeric interferece confirmed	No
Model for separation prediction	No	Lipid Identification Software	MS-DIAL
Data manipulation	Smoothing, Centroiding, Manual Integration	Nomenclature for intact lipid molecule	Yes
Nomenclature for fragment ions	Yes		

16) EtherLPC-O (EtherLPC-P)[M+H]+ / Lipid quantification

Quantitative	Yes	MS Level for quantification	MS^1

Internal lipid standard(s) MS^1

Internal standard	Endogenous subclass
LPC 18:1(d7)	EtherLPC-O (EtherLPC-P)

Type of quantification	Internal standard amount	Response correction	No
Type I isotope correction	No	Limit of quantification	Signal threshold
Normalization to reference	Yes	Lipid Quantification Software	MS-DIAL
Batch correction	No		

17) SM[M+H]+ / Lipid identification

Lipid class	SM	MS Level for identification	MS^2
Identification level	Molecular species level	MS^2 adduct	[M+H]+

Fragments for identification

Fragment name
C5H15NO4P+ Phosphorylcholine
SPB Side chain 1 -H2O

Isotope correction at MS^2	No	MS^2 verified by standard	Yes
Background check at MS^2	No	Did you presume assumptions for identification?	No
Check on:	Isomeric overlap, Isobaric overlap	Limit of detection	Signal threshold
RT verified by standard	Yes	Separation of isobaric/isomeric interferece confirmed	No
Model for separation prediction	No	Lipid Identification Software	MS-DIAL
Data manipulation	Smoothing, Centroiding, Manual Integration	Nomenclature for intact lipid molecule	Yes
Nomenclature for fragment ions	Yes		

17) SM[M+H]+ / Lipid quantification

Quantitative	Yes	MS Level for quantification	MS^1

Internal lipid standard(s) MS^1

Internal standard	Endogenous subclass
SM 18:1;2O/18:1(d9)	SM

Type of quantification	Internal standard amount	Response correction	No
Type I isotope correction	No	Limit of quantification	Signal threshold
Normalization to reference	Yes	Lipid Quantification Software	MS-DIAL
Batch correction	No		

18) SM[M+HCOO]- / Lipid identification

Lipid class	SM	MS Level for identification	MS^2
Identification level	Molecular species level	MS^2 adduct	[M+HCOO]-

Fragments for identification

Fragment name
NL of Acyl Side chain 2 + CH3

Isotope correction at MS^2	No	MS^2 verified by standard	Yes
Background check at MS^2	No	Did you presume assumptions for identification?	No
Check on:	Isomeric overlap, Isobaric overlap	Limit of detection	Signal threshold
RT verified by standard	Yes	Separation of isobaric/isomeric interferece confirmed	No
Model for separation prediction	No	Lipid Identification Software	MS-DIAL
Data manipulation	Smoothing, Centroiding, Manual integration	Nomenclature for intact lipid molecule	Yes
Nomenclature for fragment ions	Yes		

18) SM[M+HCOO]- / Lipid quantification

Quantitative	No	Normalization to reference	No
Batch correction	No		

19) LPE[M+H]+ / Lipid identification

Lipid class	LPE	MS Level for identification	MS^2
Identification level	Molecular species level	MS^2 adduct	[M+H]+
Fragments for identification			

Fragment name
NL of C2H8NO4P Ethanolaminephosphate

Isotope correction at MS^2	No	MS^2 verified by standard	Yes
Background check at MS^2	No	Did you presume assumptions for identification?	No
Check on:	Isomeric overlap, Isobaric overlap	Limit of detection	Signal threshold
RT verified by standard	Yes	Separation of isobaric/isomeric interferece confirmed	No
Model for separation prediction	No	Lipid Identification Software	MS-DIAL
Data manipulation	Smoothing, Centroiding, Manual integration	Nomenclature for intact lipid molecule	Yes
Nomenclature for fragment ions	Yes		

19) LPE[M+H]+ / Lipid quantification

Quantitative	No	Normalization to reference	No
Batch correction	No		

20) LPE[M-H]- / Lipid identification

Lipid class	LPE	MS Level for identification	MS^2
Identification level	Molecular species level	MS^2 adduct	[M-H]-
Fragments for identification			

Fragment name
FA Side chain 1

Isotope correction at MS^2	No	MS^2 verified by standard	Yes
Background check at MS^2	No	Did you presume assumptions for identification?	No
Check on:	Isomeric overlap, Isobaric overlap	Limit of detection	Signal threshold
RT verified by standard	Yes	Separation of isobaric/isomeric interferece confirmed	No
Model for separation prediction	No	Lipid Identification Software	MS-DIAL
Data manipulation	Smoothing, Centroiding, Manual integration	Nomenclature for intact lipid molecule	Yes
Nomenclature for fragment ions	Yes		

20) LPE[M-H]- / Lipid quantification

Quantitative	Yes	MS Level for quantification	MS^1

Internal lipid standard(s) MS^1

Internal standard	Endogenous subclass
LPE 18:1(d7)	LPE

Type of quantification	Internal standard amount	Response correction	No
Type I isotope correction	No	Limit of quantification	Signal threshold
Normalization to reference	Yes	Lipid Quantification Software	MS-DIAL
Batch correction	No		

21) EtherLPE-O (EtherLPE-P)[M-H]- / Lipid identification

Lipid class	EtherLPE-O (EtherLPE-P)	MS Level for identification	MS^2
Identification level	Molecular species level	MS^2 adduct	[M-H]-

Fragments for identification

Fragment name
C2H7NO4P- Phosphoethanolamine
Ether-O (Ether-P) Side chain 1

Isotope correction at MS^2	No	MS^2 verified by standard	No
Background check at MS^2	No	Did you presume assumptions for identification?	No
Check on:	Isomeric overlap, Isobaric overlap	Limit of detection	Signal threshold
RT verified by standard	No	Separation of isobaric/isomeric interferece confirmed	No
Model for separation prediction	No	Lipid Identification Software	MS-DIAL
Data manipulation	Smoothing, Centroiding, Manual Integration	Nomenclature for intact lipid molecule	Yes
Nomenclature for fragment ions	Yes		

21) EtherLPE-O (EtherLPE-P)[M-H]- / Lipid quantification

Quantitative	Yes	MS Level for quantification	MS^1

Internal lipid standard(s) MS^1

Internal standard	Endogenous subclass
LPE 18:1(d7)	EtherLPE-O (EtherLPE-P)

Type of quantification	Internal standard amount	Response correction	No
Type I isotope correction	No	Limit of quantification	Signal threshold
Normalization to reference	Yes	Lipid Quantification Software	MS-DIAL
Batch correction	No		

22) PG[M-H]- / Lipid identification

Lipid class	PG	MS Level for identification	MS^2
Identification level	Molecular species level	MS^2 adduct	[M-H]-

Fragments for identification

Fragment name
C3H6O5P- Phosphoglycerol
FA Side chain 1
FA Side chain 2

Isotope correction at MS^2	No	MS^2 verified by standard	Yes
Background check at MS^2	No	Did you presume assumptions for identification?	No
Check on:	Isomeric overlap, Isobaric overlap	Limit of detection	Signal threshold
RT verified by standard	Yes	Separation of isobaric/isomeric interferece confirmed	No
Model for separation prediction	No	Lipid Identification Software	MS-DIAL
Data manipulation	Smoothing, Centroiding, Manual Integration	Nomenclature for intact lipid molecule	Yes
Nomenclature for fragment ions	Yes		

22) PG[M-H]- / Lipid quantification

Quantitative	Yes	MS Level for quantification	MS^1

Internal lipid standard(s) MS^1

Internal standard	Endogenous subclass
PG 15:0_18:1(d7)	PG

Type of quantification	Internal standard amount	Response correction	No
Type I isotope correction	No	Limit of quantification	Signal threshold
Normalization to reference	Yes	Lipid Quantification Software	MS-DIAL
Batch correction	No		

23) PI[M-H]- / Lipid identification

Lipid class	PI	MS Level for identification	MS^2
Identification level	Molecular species level	MS^2 adduct	[M-H]-

Fragments for identification

Fragment name
C6H10O8P- Phosphoinositol
FA Side chain 1
FA Side chain 2

Isotope correction at MS^2	No	MS^2 verified by standard	Yes
Background check at MS^2	No	Did you presume assumptions for identification?	No
Check on:	Isomeric overlap, Isobaric overlap	Limit of detection	Signal threshold
RT verified by standard	Yes	Separation of isobaric/isomeric interferece confirmed	No
Model for separation prediction	No	Lipid Identification Software	MS-DIAL
Data manipulation	Smoothing, Centroiding, Manual Integration	Nomenclature for intact lipid molecule	Yes
Nomenclature for fragment ions	Yes		

23) PI[M-H]- / Lipid quantification

Quantitative	Yes	MS Level for quantification	MS^1
Internal lipid standard(s) MS^1			

Internal standard	Endogenous subclass
PI 15:0_18:1(d7)	PI

Type of quantification	Internal standard amount	Response correction	No
Type I isotope correction	No	Limit of quantification	Signal threshold
Normalization to reference	Yes	Lipid Quantification Software	MS-DIAL
Batch correction	No		

24) PS[M-H]- / Lipid identification

Lipid class	PS	MS Level for identification	MS^2
Identification level	Molecular species level	MS^2 adduct	[M-H]-
Fragments for identification			

Fragment name
NL of C3H6NO2 Phosphotidylserine
FA Side chain 1
FA Side chain 2

Isotope correction at MS^2	No	MS^2 verified by standard	Yes
Background check at MS^2	No	Did you presume assumptions for identification?	No
Check on:	Isomeric overlap, Isobaric overlap	Limit of detection	Signal threshold
RT verified by standard	Yes	Separation of isobaric/isomeric interferece confirmed	No
Model for separation prediction	No	Lipid Identification Software	MS-DIAL
Data manipulation	Smoothing, Centroiding, Manual Integration	Nomenclature for intact lipid molecule	Yes
Nomenclature for fragment ions	Yes		

24) PS[M-H]- / Lipid quantification

Quantitative	Yes	MS Level for quantification	MS^1
Internal lipid standard(s) MS^1			

Internal standard	Endogenous subclass
PS 15:0_18:1(d7)	PS

Type of quantification	Internal standard amount	Response correction	No
Type I isotope correction	No	Limit of quantification	Signal threshold
Normalization to reference	Yes	Lipid Quantification Software	MS-DIAL
Batch correction	No		

25) PG[M+NH4]+ / Lipid identification

Lipid class	PG	MS Level for identification	MS^2
Identification level	Molecular species level	MS^2 adduct	[M+NH4]+

Fragments for identification

Fragment name
NL of C3H906 P +NH3 Characteristic Fragment
DMAG Side chain 1
DMAG Side chain 2

Isotope correction at MS^2	No	MS^2 verified by standard	Yes
Background check at MS^2	No	Did you presume assumptions for identification?	No
Check on:	Isomeric overlap, Isobaric overlap	Limit of detection	Signal threshold
RT verified by standard	Yes	Separation of isobaric/isomeric interferece confirmed	No
Model for separation prediction	No	Lipid Identification Software	MS-DIAL
Data manipulation	Smoothing, Centroiding, Manual Integration	Nomenclature for intact lipid molecule	Yes
Nomenclature for fragment ions	Yes		

25) PG[M+NH4]+ / Lipid quantification

Quantitative	No	Normalization to reference	No
Batch correction	No		

26) PI[M+NH4]+ / Lipid identification

Lipid class	PI	MS Level for identification	MS^2
Identification level	Molecular species level	MS^2 adduct	[M+NH4]+

Fragments for identification

Fragment name
NL of C6H1409P+NH3 Phosphoinositol

Isotope correction at MS^2	No	MS^2 verified by standard	Yes
Background check at MS^2	No	Did you presume assumptions for identification?	No
Check on:	Isomeric overlap, Isobaric overlap	Limit of detection	Signal threshold
RT verified by standard	Yes	Separation of isobaric/isomeric interferece confirmed	No
Model for separation prediction	No	Lipid Identification Software	MS-DIAL
Data manipulation	Smoothing, Centroiding, Manual integration	Nomenclature for intact lipid molecule	Yes
Nomenclature for fragment ions	Yes		

26) PI[M+NH4]+ / Lipid quantification

Quantitative	No	Normalization to reference	No
Batch correction	No		

27) PS[M+H]+ / Lipid identification

Lipid class	PS	MS Level for identification	MS^2
Identification level	Molecular species level	MS^2 adduct	[M+H]+

Fragments for identification

Fragment name
NL of C3H8NO6P Phosphotidylserine

Isotope correction at MS^2	No	MS^2 verified by standard	Yes
Background check at MS^2	No	Did you presume assumptions for identification?	No
Check on:	Isomeric overlap, Isobaric overlap	Limit of detection	Signal threshold
RT verified by standard	Yes	Separation of isobaric/isomeric interferece confirmed	No
Model for separation prediction	No	Lipid Identification Software	MS-DIAL
Data manipulation	Smoothing, Centroiding, Manual Integration	Nomenclature for intact lipid molecule	Yes
Nomenclature for fragment ions	Yes		

27) PS[M+H]+ / Lipid quantification

Quantitative	No	Normalization to reference	No
Batch correction	No		

Table S2. (separate file)
List of diagnostic spectra ion for manual validation of lipid annotation

Table S3. (separate file)
In vitro #1 and *In vitro* #2 lipidomics - Lipid quantification and effect size with age

Table S4. (separate file)
Lipid overlap between *in vitro* datasets

Table S5. (separate file)
In vivo isolated qNSC lipidomics - Lipid quantification and effect size with age

Table S6. (separate file)
DESI-MSI lipidomics - Metabolite intensity and cell type composition

Table S7. (separate file)
GPMV lipidomics - Lipid quantification and effect size with age

Table S8. (separate file)
sgRNA sequences for *in vitro* knockout

Table S9. (separate file)
In vitro knockout lipidomics - Lipid quantification and effect size in each knockout compared to control

Table S10. (separate file)
Mboat2 HCR probes and primers used in qRT-PCR

Table S11. (separate file)
Complete sequence of MBOAT2 overexpression vector

Table S12. (separate file)
Mboat2 overexpression lipidomics - Lipid quantification

Table S13. (separate file)
Young plasma membrane lipid supplementation lipidomics - Lipid quantification

Table S14. (separate file)
Data and statistics
